# Revealing biases in insect observations: A comparative analysis between academic and citizen science data

**DOI:** 10.1371/journal.pone.0305757

**Published:** 2024-07-18

**Authors:** Joan Díaz-Calafat, Sebastià Jaume-Ramis, Karen Soacha, Ana Álvarez, Jaume Piera

**Affiliations:** 1 Southern Swedish Forest Research Centre, Swedish University of Agricultural Sciences, Lomma, Sweden; 2 Mediterranean Parasitology and Ecoepidemiology Research Group, Department of Biology, University of the Balearic Islands, Palma, Spain; 3 EMBIMOS Research Group, Institute of Marine Sciences (ICM), Spanish National Research Council (CSIC), Barcelona, Spain; 4 Doctorate Program in Information and Knowledge Society, Open University of Catalonia (UOC), Barcelona, Spain; USDA Forest Service Southern Research Station, UNITED STATES OF AMERICA

## Abstract

Citizen Science is a powerful tool for biodiversity research, as it facilitates data recording at large scales that would otherwise be impossible to cover by standard academic research. Despite its benefits, the accuracy of citizen science data remains a subject of concern among scientists, with varying results reported so far. Neither citizen science data nor academic records are immune to biases, which can significantly impact the quality and reliability of observations. Here, using insects in the Iberian Peninsula as a case study, we compare data collected by participatory platforms to those obtained through academic research projects, and assess their taxonomic, spatial, temporal, and environmental biases. Results show a prominent taxonomic bias in both academic and citizen science data, with certain insect orders receiving more attention than others. These taxonomic biases are conserved between different participatory platforms, as well as between groups of users with different levels of contribution performance. The biases captured by leading contributors in participatory platforms mirrored those of sporadic users and academic data. Citizen science data had higher spatial coverage and less spatial clustering than academic data, showing also clearer trends in temporal seasonality. Environmental coverage over time was more stable in citizen science than in academic records. User behaviour, preference, taxonomical expertise, data collection methodologies and external factors may contribute to these biases. This study shows the multifaceted nature of biases present in academic records and citizen science platforms. The insights gained from this analysis emphasize the need for careful consideration of these biases when making use of biodiversity data from different sources. Combining academic and citizen science data enhances our understanding of biodiversity, as their integration offers a more comprehensive perspective than relying solely on either dataset alone, especially since biases in these two types of data are not always the same.

## 1. Introduction

Recording biodiversity is crucial for species conservation. Over the past few decades, Citizen Science (herein CS) has emerged as a potent tool for biodiversity recording and ecological research [1–4]. By harnessing collective voluntary efforts, CS facilitates the collection and recording of species observations from a local to a global scale, contributing significantly to the monitoring of ecosystem structure and function [1]. The essence of CS lies in the active participation of individuals who engage in the collection and recording of biodiversity data out of personal interests and curiosity. This may reduce the financial and time costs associated with species biodiversity recording and may be particularly productive in environments frequently visited by volunteers [5], while at the same time providing reliable distributional data for underdocumented species of high conservation priority [6].

In response to the growing interest in CS, numerous platforms have been developed to facilitate the process of species recording, incorporating features such as mobile apps to facilitate the user experience to conveniently record species anytime, anywhere. Also, advancements in smartphone technologies, encompassing improved cameras, internet connectivity and GPS have not only enhanced the communication between volunteers and researchers but improved and ensured both data quality and storage [7–9].

Projects making use of CS for data recording are diverse, ranging from biodiversity recording *sensu lato* to the recording of targeted species, such as invasive species. As a result, CS-based projects have increased in popularity across different research areas, including biogeography and conservation biology [10, 11]. The mutual benefits derived from CS are noteworthy, as researchers gain access to valuable data on a potentially broad geographical extent that would have otherwise been impossible to sample by a reduced group of researchers, while volunteers acquire new knowledge, skills and attitudes such as species identification and a deeper understanding of nature and their surroundings [12, 13].

Despite the well-documented benefits of CS, several biases have been reported in the recorded data, such as geographical biases arising from uneven sampling in inaccessible areas, and biases related to species recording and identification, where certain species are easier to identify and to record than others. Consequently, it is imperative to acknowledge and address these biases, especially when using recorded data for statistical analyses [14]. Addressing these biases enhances the reliability of CS-based studies, ensuringresearchers work with more accurate and robust data. Moreover, understanding volunteers’ behaviour can support better use of the data. For instance, volunteers’ records exhibit a stronger correlation with readily accessible locations, particularly those associated with recreation and vacation properties [15].

Here, we focus on insects as they comprise a huge number of the described animal species [16]. We have also chosen the Iberian Peninsula as the geographical extent of this work, as it is considered a biodiversity hotspot, with almost 98% of its fauna represented by invertebrates [17]. Particularly, we focus on the comparison between academic and CS data. Our goal is to identify taxonomic, spatial, temporal and environmental biases and compare them in both types of data, contributing to a deeper understanding of the strengths and limitations between CS and academic biodiversity recording data.

## 2. Materials and methods

### 2.1 Data download: GBIF and “Natusfera_2022” datasets

All available occurrence data belonging to the class Insecta from the Iberian Peninsula (mainland Spain and Portugal) up to the end of 2022 were downloaded from the Global Biodiversity Information Facility (gbif.org) on the 3rd of June 2023. This resulted in 1,955,449 occurrence records from 387 datasets. Each dataset was sorted either as containing “academic records” (i.e., data coming from scientific studies, collections or monitoring programs) or “citizen science records” (i.e., data belonging to citizen science recording platforms or data collected without following a standardized protocol). In order to classify the occurrences in these categories, we used the variable “basisOfRecord” from the GBIF Darwin Core Archive, which portrays the specific nature of the data record. In our dataset, there were nine different levels for this variable: “HUMAN_OBSERVATION” (defined by the Darwin Core Archive as “an output of a human observation process, without physical evidence nor evidence captured with a machine”), “OBSERVATION” (i.e., “an occurrence record resulting from an observation process”), “PRESERVED_SPECIMEN” (i.e., “a specimen that has been preserved in a collection or a museum”), “MACHINE_OBSERVATION” (i.e., “an output of a machine observation process, such as a remote sensing image or an occurrence record based on telemetry”), “MATERIAL_SAMPLE” (i.e., “a physical result of a sampling or subsampling event, such as a whole organism preserved in a collection or a part of an organism isolated for some purpose”), “LITERATURE” (i.e., “records found in literature”), “FOSSIL_SPECIMEN” (i.e., “a preserved specimen that is a fossil”), “LIVING_SPECIMEN” (i.e., “a specimen that is alive, such as a living plant in a botanical garden or a living animal in a zoo”) and “UNKNOWN” (i.e., “occurrences that lacked any of the aforementioned categories”). Occurrences from preserved specimens, material samples and literature records were considered to be inherently “academic”. All entries labelled as “obtained through machine observation” belonged to the Department of Forest Sciences of the University of Helsinki, the *Museu de Ciències Naturals de Barcelona* and the Xeno-canto Foundation for Nature Sounds. The first two publishers were considered “academic”, as their observations came from standardized light trap monitoring, literature, or digitized collections. The last publisher is a participatory platform where users can upload nature sounds, and insect data consisted mainly of Orthoptera sound recordings, so it was considered “citizen science”. Occurrence records labelled as “human observation” or simply as “observations” were classified either as “academic” or “citizen science-based” according to the metadata available on the description of their datasets in the GBIF portal. Data exclusively obtained through scientific projects were considered "academic", whilst occurrences obtained through unstandardized sampling or belonging to participatory recording platforms were considered as "citizen science". See S1 Table for an overview of the number of observations for academic and CS records, for each category of “basisOfRecord” in the Darwin Archive Core. See also S2 Table for an overview of all the GBIF datasets and their categorization as either “academic” or “citizen science-based”.

Insect occurrences from “unknown” recording nature, “living specimens” or “fossil records” were dismissed and thus excluded from the database and subsequent analyses. This is because living specimens (as described by the Darwin Core Archive) were not considered to reflect actual wild biodiversity and fossil records were reckoned to be recorded mostly by “academic” records. Overall, these occurrences accounted for only 0.33% of the data, and considering the amount of data used in the analyses it is not likely that their removal influenced the results.

Similarly, occurrence records for the class Insecta were downloaded from Natusfera (www.natusfera.gbif.es). Natusfera was a citizen science platform that was active from 2017 to 2022. The initial community of this platform is currently migrating and integrating into two new communities, one linked to the MINKA platform [18] and the other to the Spanish iNaturalist community [19], which has inherited the name of the old platform. Given the transitory status of the communities reporting in Natusfera, their associated records are not yet included in the GBIF. In total, 30,602 insect occurrences recorded in Spain and Portugal were obtained, and are referred to as “Natusfera_2022” from here on. All these records were considered as CS records prior to merging with GBIF data. Minor changes in variable names and insect order names were made for consistency between both datasets before merging.

### 2.2 Data cleaning

Our analyses encompassed both non-spatial and spatial aspects. In light of this, the data underwent a cleaning process at two different levels: a general cleaning for the analyses with no spatial component, and a more meticulous cleaning for spatial analyses. This is because data with no coordinates could be used for some of the analyses (e.g., taxonomical bias), but needed to be discarded for spatial analyses. Thus, general data cleaning involved keeping occurrences with no coordinate data, while checking occurrences with coordinates using the *clean_coordinates* function in the ‘CoordinateCleaner’ package [20] in the R software [21]. This R package allows users to perform different tests on a set of coordinate records, flagging and removing problematic records. We ensured that all coordinates fell within the geographical boundaries of the country specified in the metadata, excluding any points located in the Atlantic Ocean or Mediterranean Sea. Additionally, occurrences that only recorded the presence of insects with no further taxonomic precision were removed (i.e., records with no taxonomic order data). Duplicated records were removed. This resulted in a total of 1,868,836 clean records. For the meticulous cleaning involving data used in spatial analyses, in addition to the aforementioned cleaning steps, occurrence records with no coordinates were removed, as were those with low (> 10 km) coordinate accuracy. Coordinates with no available coordinate accuracy data were considered as precise as their decimal places indicated. Besides this, further tests were implemented, including the removal of coordinates within a 1 km radius of country capitals and country centroids. Records within 100 m of a list of biodiversity institutions integrated into the ’CoordinateCleaner’ package were also excluded. These measures aimed to eliminate potentially erroneous spatial records, as poorly geo-referenced occurrence records are often erroneously geo-referenced to centroids [20]. After this step, 1,240,612 occurrences remained.

### 2.3 Statistical analyses

We conducted a set of analyses to assess different types of bias in academic and citizen science data. Namely, we addressed taxonomic, spatial, temporal and environmental biases.

#### 2.3.1 Taxonomic bias

We focused on three aspects of taxonomic bias: (i) variations in the ratio of observations and the count of species documented per insect order, (ii) the influence of user behaviour and preferences on these variations in observation ratios and species counts and (iii) the correlation between the proportion of observations for a species and their spatial distribution.

*2*.*3*.*1*.*1 Variations in the ratio of observations and the count of species documented per order*. All available data were standardized by calculating the percentage of observations and species per insect order. This was done separately for CS and academic records. Afterwards, a barplot was built to visualize the relative abundance of observations per insect species across CS and academic records to assess whether biases in data collection were preserved between CS and academic records. For clarity, insect orders that accumulated less than 0.5% of all the observations were excluded from this visualization.

To quantify and assess whether recording biases were consistent across different participatory platforms and academic records, the previously cleaned CS data from GBIF were subset based on some participatory portals (iNaturalist, Observation.org, Naturgucker.de, Biodiversity4All) and compared to the “Natusfera_2022” dataset and the GBIF academic records. This comparison was made based on the total number of observations per insect order. Data were log-transformed for better visualization and scatterplots with a regression line were built. Non-parametric Spearman correlations among all platforms were run.

*2*.*3*.*1*.*2 Influence of user behaviour and preferences on the variations in observation ratios and species counts in the “Natusfera_2022” dataset*. Different users may have different preferences when it comes to recording biodiversity in participatory platforms. Some might have a preference for a given insect group, and some others may record any type of insect indiscriminately. We visualized the user recording behaviour using the “Natusfera_2022” dataset as a case study by making a user matrix that summarized the percentage of records per insect order for each user. Only users with more than 10 insect observations were taken into account. Then, the matrix was sorted by the most popular order (Lepidoptera) for an easier representation of user preferences. We also assessed, through Pearson’s correlation tests, the relationship between the (log-transformed) number of users uploading observations in each insect order and the (log-transformed) number of observations per order, the number of users per order and Shannon’s diversity index per order, and Shannon’s index and the number of observations per order.

Furthermore, we segmented users in the “Natusfera_2022” dataset based on their contribution performance in observations on the platform. We ran an outlier test to find the most active users (“leading contributors” from now on) on the “Natusfera_2022” dataset (21 users out of 857, accounting for 82.41% of all observations). Likewise, we selected the least productive users (“sporadic users” from now on) as those with 10 or fewer observations (753 users accounting for 5.05% of all observations, of which 34.13% only recorded a single observation). Spearman’s correlation test was used to compare the number of observations in each insect order recorded by these two groups of users. This was done in order to assess whether the taxonomic biases obtained by leading contributors (i.e., more likely in well-established participatory platforms) were the same as those obtained through sporadic users (i.e., more common in young or short-lived participatory platforms). Moreover, Shannon’s diversity index was calculated for each insect order in each of these categories: data collected by leading contributors, data collected by sporadic users and academic record data. Then, Pearson’s correlation was used to assess the relationship between Shannon’s diversity captured by each set of users and academic data.

*2*.*3*.*1*.*3 Correlation between the proportion of observations for a species and its spatial distribution*. With the deep-cleaned data for spatial analysis, we calculated the degree of proportionality between the number of grid cells (at a 0.25 degrees of resolution) occupied by each insect species (i.e., a proxy for species’ range size) and the total number of records of each respective species. This was done for each period of 10 years between 1982 and 2022, calculating the species range independently for each period. A regression of the number of records per range size was run for each 10-year period, and the r^2^ value from each linear regression was used as an index of proportionality between range sizes and number of records per species. High values indicate that the species are being sampled proportionately to their range (i.e., no or low bias), and low values indicate that species are over- or undersampled. This analysis was done with the ‘occAssess’ R package [22].

#### 2.3.2 Spatial bias

The R package ‘occAssess’ [22] was used to assess spatial bias. Firstly, the log-transformed number of observations was plotted on a grid of 0.25 degrees of resolution. This was done for both the academic and the CS records. Following that, spatial bias was quantified by simulating a number of random points equal to each dataset (i.e., academic and CS) and calculating the average nearest neighbour distance across our data, divided by the nearest neighbour distance from the random sample. In total, 99 iterations of random points were used. If this index is lower than one, it means that data are more clustered than expected from a random sample of points. This was done independently for all 10-year periods of time between 1982 and 2022.

Additionally, we used the R package ‘sampbias’ [23] to assess the impact of geographical features related to human accessibility on the spatial bias of both academic and CS data. This package uses a Bayesian approach to estimate how occurrence frequency varies depending on the proximity of multiple spatial features. We selected roads, cities and natural areas as spatial features to be analysed. Road and city data were downloaded through the ‘rnaturalearth’ package [24], and protected natural areas were downloaded from the World Database on Protected Areas [25]. Analyses were conducted at 0.25 degrees of resolution.

#### 2.3.3 Temporal bias

Temporal patterns in the recording behaviour of users in participatory platforms were explored using the R package ‘forecast’ [26]. Academic and CS data from the last 10 years (2012–2022) were used for this analysis.

To assess trends and seasonality in the Natusfera and academic records, we used a contingency table of the dates in which observations were made by users (variable “eventDate” from the Darwin Core Archive). In addition to that, the seasonal components of the data were studied, namely patterns based on the day of the week in which observations were made and national holidays. National holidays considered for Spain and Portugal were: the Epiphany (6^th^ of January), Good Friday (between March and April, depending on the year), Freedom Day (25^th^ of April), Labour Day (1^st^ of May), Corpus Christi (between May and June, depending on the year), Portugal Day (10^th^ of June), *Asunción de la Virgen* (15^th^ of August), Implementation of the Portuguese Republic (5^th^ of October), Hispanity Day (12^th^ of October), All Saints’ Day (1^st^ of November), Restoration Day (1^st^ of December), Spanish Constitution Day (6^th^ of December), *La inmaculada Concepción* (8^th^ of December) and Christmas (25^th^ of December). When falling on a weekend (e.g., Easter), the holiday effect was not considered to avoid confounding it with weekend effects. Only official holidays were included in this analysis, and not variations that may differ regionally, e.g., when holidays on a weekend are extended to either Friday or Monday. Also, vacation time (generally in summer) was not considered, as this may vary in different jobs and by different users. New Year’s Eve had to be removed as a holiday from this analysis, as most occurrence records with no date default to the 1^st^ of January, making this date disproportionally frequent compared to other dates.

#### 2.3.4 Environmental bias

In a dataset that is not spatially biased there can still be environmental bias, and the other way around. To assess to what extent academic and CS records were biased across time periods in environmental space, we used 15 bioclimatic variables downloaded from CHELSA V.2.1 [27]. These variables are calculated from monthly temperature and precipitation data and correspond to the period 1981–2010. The variables used were: Bio1 (Annual Mean Temperature), Bio2 (Mean Diurnal Range), Bio3 (Isothermality), Bio4 (Temperature Seasonality), Bio5 (Max Temperature of Warmest Month), Bio6 (Min Temperature of Coldest Month), Bio7 (Temperature Annual Range), Bio10 (Mean Temperature of Warmest Quarter), Bio11 (Mean Temperature of Coldest Quarter), Bio12 (Annual Precipitation), Bio13 (Precipitation of Wettest Month), Bio14 (Precipitation of Driest Month), Bio15 (Precipitation Seasonality), Bio16 (Precipitation of Wettest Quarter) and Bio17 (Precipitation of Driest Quarter). The following variables were excluded, as they produced spatial artifacts that might affect model outputs [28]: Bio8 (Mean Temperature of Wettest Quarter), Bio9 (Mean Temperature of Driest Quarter), Bio18 (Precipitation of Warmest Quarter), Bio19 (Precipitation of Coldest Quarter). All the environmental data from these 15 variables were extracted for each coordinate in both the academic record dataset and the CS dataset. Then, a principal component analysis was performed on such data. A random set of 10000 environmental points were selected and used as background environmental data. It is in this environmental space that our occurrences were mapped. As environmental data were only available from 1981 on, records previous to that year were not included in the analysis. Data were then aggregated in periods of 10 years between 1982 and 2022.

### 2.4 Data and code availability

The data supporting this study and the code used for analyses can be found in Zenodo at https://doi.org/10.5281/zenodo.10045223. The GBIF data download can be accessed at https://doi.org/10.15468/dl.mm74qg [29].

For better reproduction of our results, we recommend using the R package ‘checkpoint’ [30] set to February 2024. This allows one to use the same version of the R packages that were applied when analysing our data.

## 3. Results

In total, academic records comprised 1,111,694 observations and 14,019 unique species. On the other hand, CS data accumulated 757,142 observations, accounting for 8,498 unique species.

### 3.1 Taxonomic bias

#### 3.1.1 Variations in the ratio of observations and the count of species documented per order

**Fig 1:**
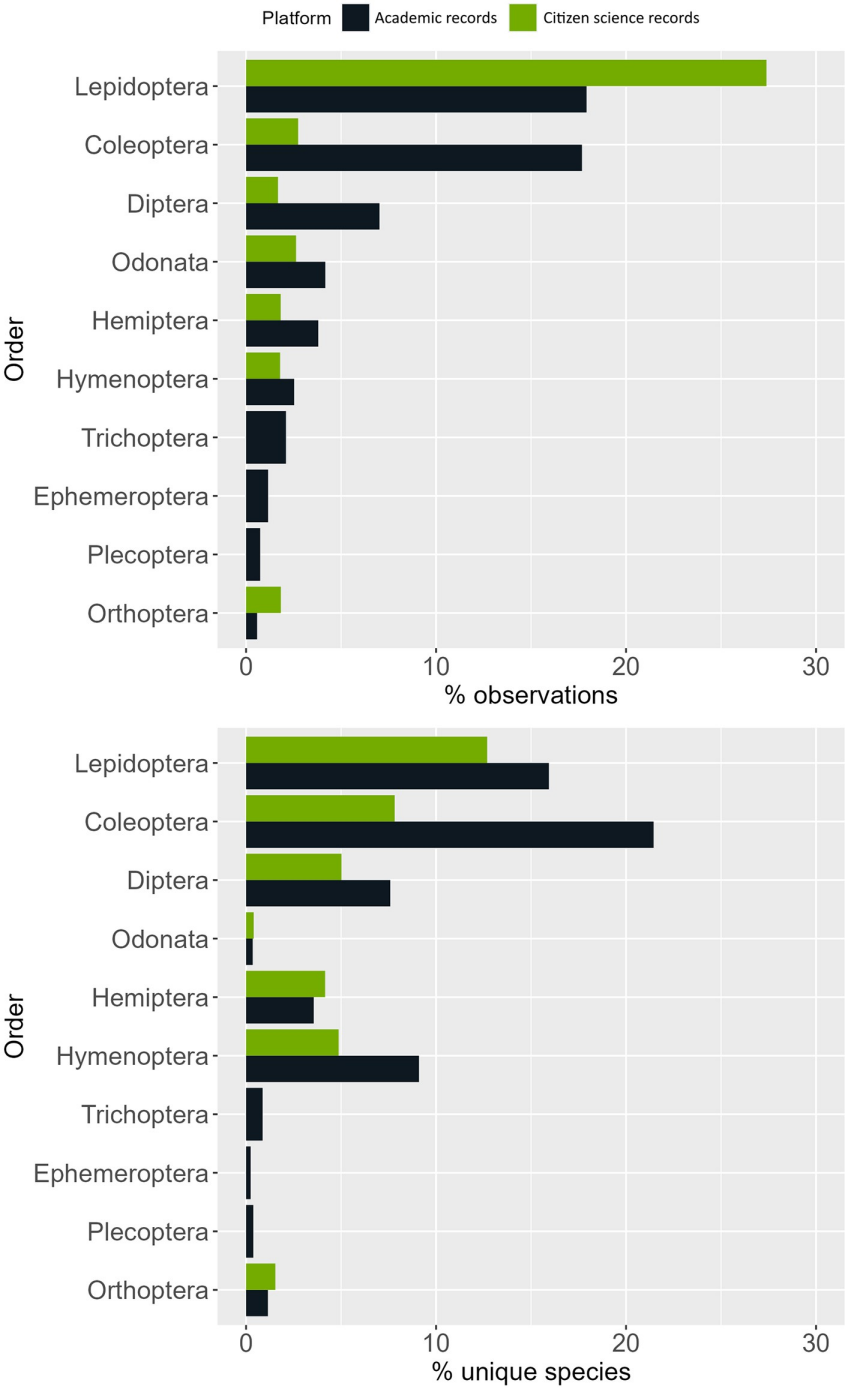
Percentage of observations (top) and unique species (bottom) for each insect order recorded through academic records (dark blue) or citizen science (green). Insect orders that accumulated less than 0.5% of all the observations were excluded from this analysis.

**Fig 2:**
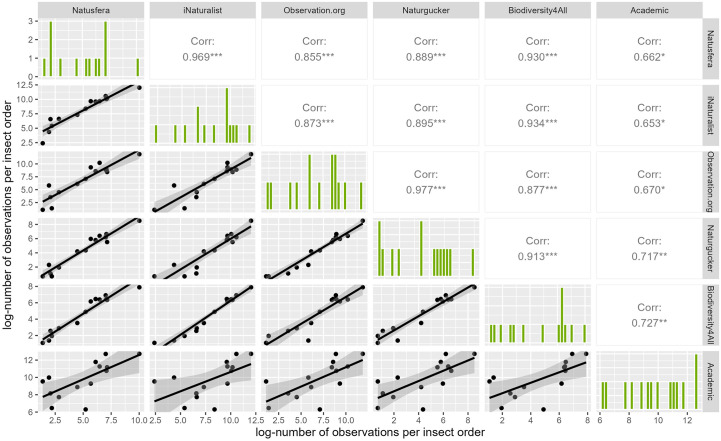
Spearman’s correlation between (log-transformed) number of observations per each insect order in popular participatory platforms and records considered as “academic”. Numbers in the grid correspond to Spearman correlation coefficients, and asterisks to statistical significance ([*] p < 0.05, [**] p < 0.01, [***] p < 0.001).

In all cases except for Lepidoptera, the academic records gathered more observations for insect order than CS records. Compared to other insect orders, Lepidoptera was the one with the most observations for both academic data and CS data. Specifically, this order corresponded to 17.92% of the academic data and 27.39% of CS, with 15.94% and 12.69% of the unique species, respectively. In CS data, all the other insect orders were up to 16 times lower in terms of observations and 32 times lower in terms of species than Lepidoptera. In academic records, Coleoptera represented almost the same number of observations as Lepidoptera (17.68%), but more unique species (21.45%). The other orders had up to 30 times fewer observations and up to 66 times lower percentage of unique species (Fig 1). Biases derived from user recording behaviour seem to be consistent across different participatory platforms (Fig 2). When running a Spearman correlation on the log-transformed number of observations per insect order among the “Natusfera_2022” dataset and popular platforms such as iNaturalist, Observation.org, Naturgucker.de and Biodiversity4All, all pair-cases were statistically significant (all p-values < 0.001) and highly correlated, with the lowest correlation coefficient being 0.86. The taxonomic biases in the academic records were also significantly correlated with those of all participatory platforms, but with a lower coefficient (0.65–0.72) and statistical significance (p-values < 0.05 and < 0.01).

#### 3.1.2 Influence of user behaviour and preferences on the variations in observation ratios and species counts in the “Natusfera_2022” dataset

**Fig 3:**
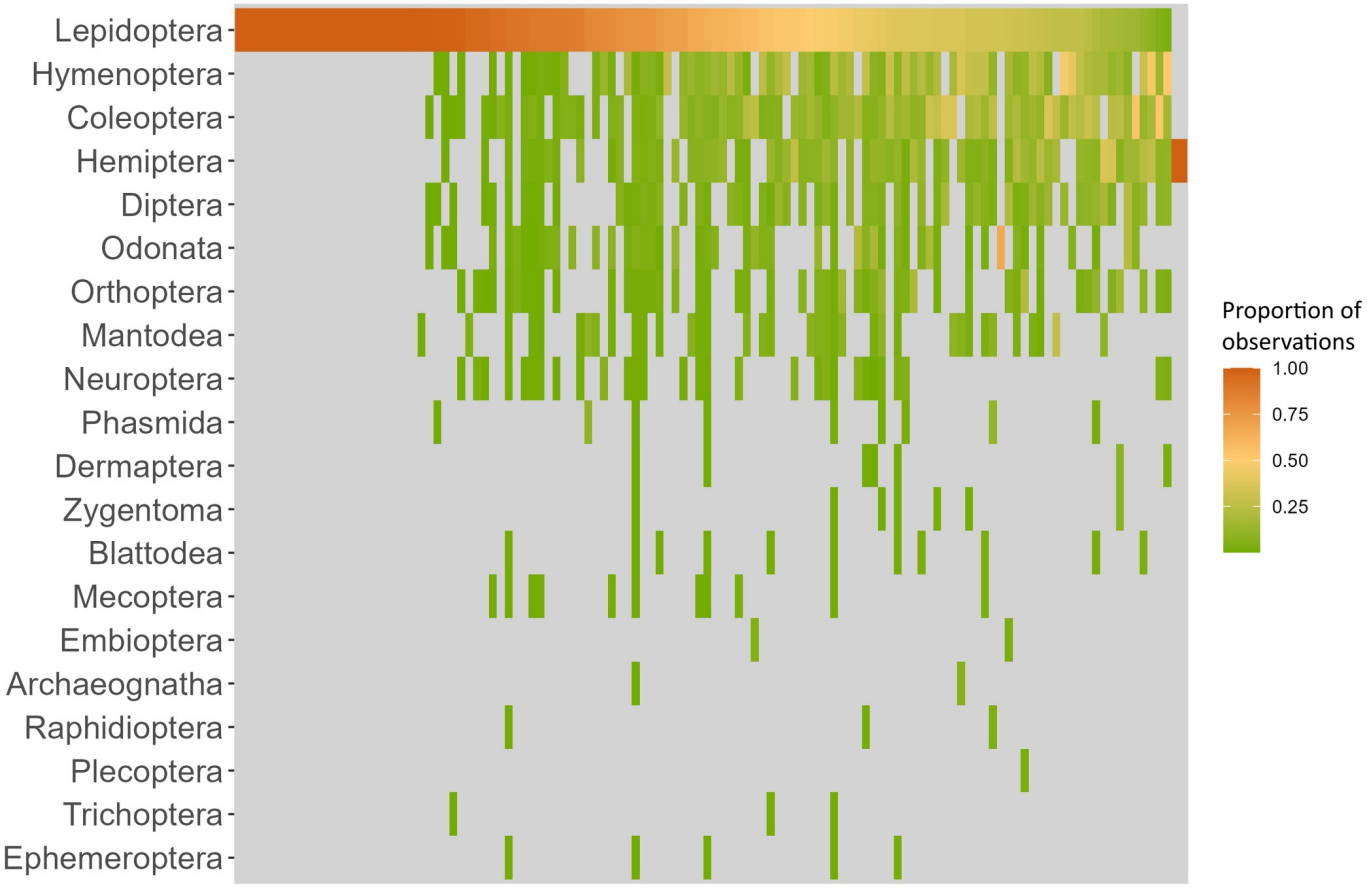
Proportion (0 to 1) of observations per user with more than 10 insect observations in the “Natusfera_2022” dataset. Each column corresponds to a single user, for which the percentage has been calculated across all their observations. Users have been sorted out regarding their relative percentage of observations belonging to the insect order Lepidoptera, as this was the most popular order recorded.

User behaviour is greatly responsible for the biases observed among the proportion of observations and unique species in each insect order. When assessing the percentage of observations uploaded per user onto each insect order, we saw that 19.17% of users with more than one observation (23 out of 120) exclusively recorded Lepidoptera, whilst only two users did not record any (Fig 3). These differences, caused by user preference when recording insects, are the ones driving the taxonomic bias observed in the “Natusfera_2022” dataset. In fact, a highly significant relationship was found between the (log-transformed) number of users uploading observations in each insect order and the (log-transformed) number of observations in each order (Pearson’s correlation test, r = 0.97, t = 17.26, df = 17, p-value < 0.001), as well as between the number of users per order and the Shannon’s diversity index of each order (Pearson’s correlation test, r = 0.96, t = 10.92, df = 11, p-value < 0.001) and the Shannon’s diversity index of each order and the total number of observations per order (Pearson’s correlation test, r = 0.94, t = 8.98, df = 11, p-value < 0.001).

Moreover, when dividing the users by their level of contribution performance, leading contributors recorded insects in the same proportion as sporadic users (Spearman’s correlation test, rho = 0.90, n = 20, S = 140.16, p-value < 0.001), showing that taxonomic bias is conserved regardless of the number of users in a participatory platform and their level of contribution performance. However, when considering Shannon’s diversity index, although the diversity captured by leading contributors was correlated with academic records (Pearson’s correlation, r = 0.86, p-value < 0.001), the same was not true for sporadic users (Pearson’s correlation, r = 0.51, p-value = 0.17).

#### 3.1.3 Correlation between the proportion of observations for a species and its spatial distribution

**Fig 4:**
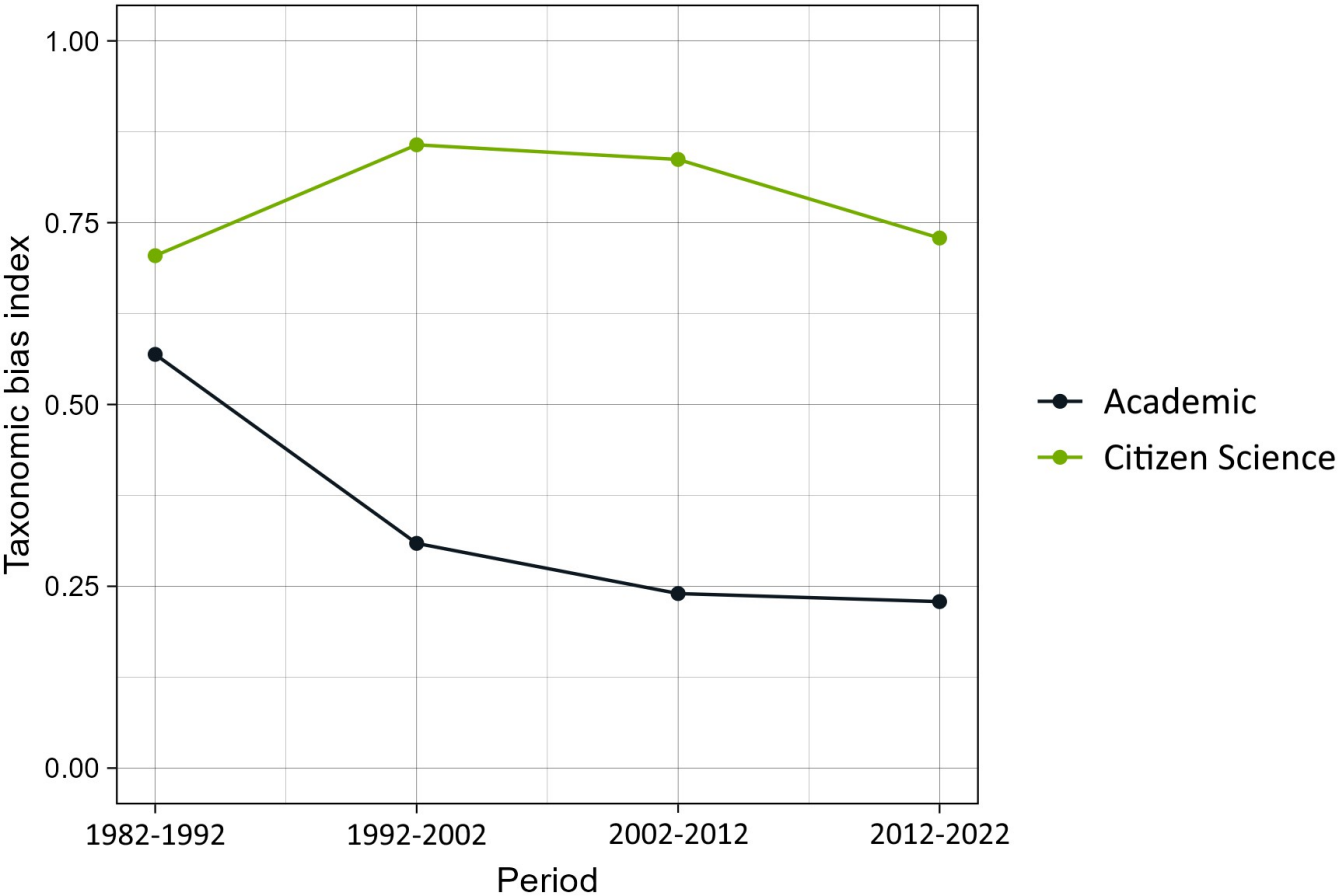
Taxonomic bias index calculated for each 10-year period between 1982 and 2022 for CS data and academic data. High values indicate that the species are being sampled proportionately to their range (i.e., no or low bias), and low values indicate that species are over- or undersampled.

Overall, the taxonomic bias index of academic records across all years (1982–2022) was 0.21, whilst for CS data was 0.72. This overall value is more similar to the index calculated for the last couple of decades (Fig 4), probably because this is when most data are available. Over time, the taxonomic index increased for CS data, and slightly decreased for academic data. This means that, at least in our dataset, CS data have less bias than academic data when taking into account the species’ distribution ranges, and that while CS data are increasingly reducing their biases by evenly covering the distribution of each species, academic data show an opposite trend, albeit much weaker.

### 3.2 Spatial bias

**Fig 5:**
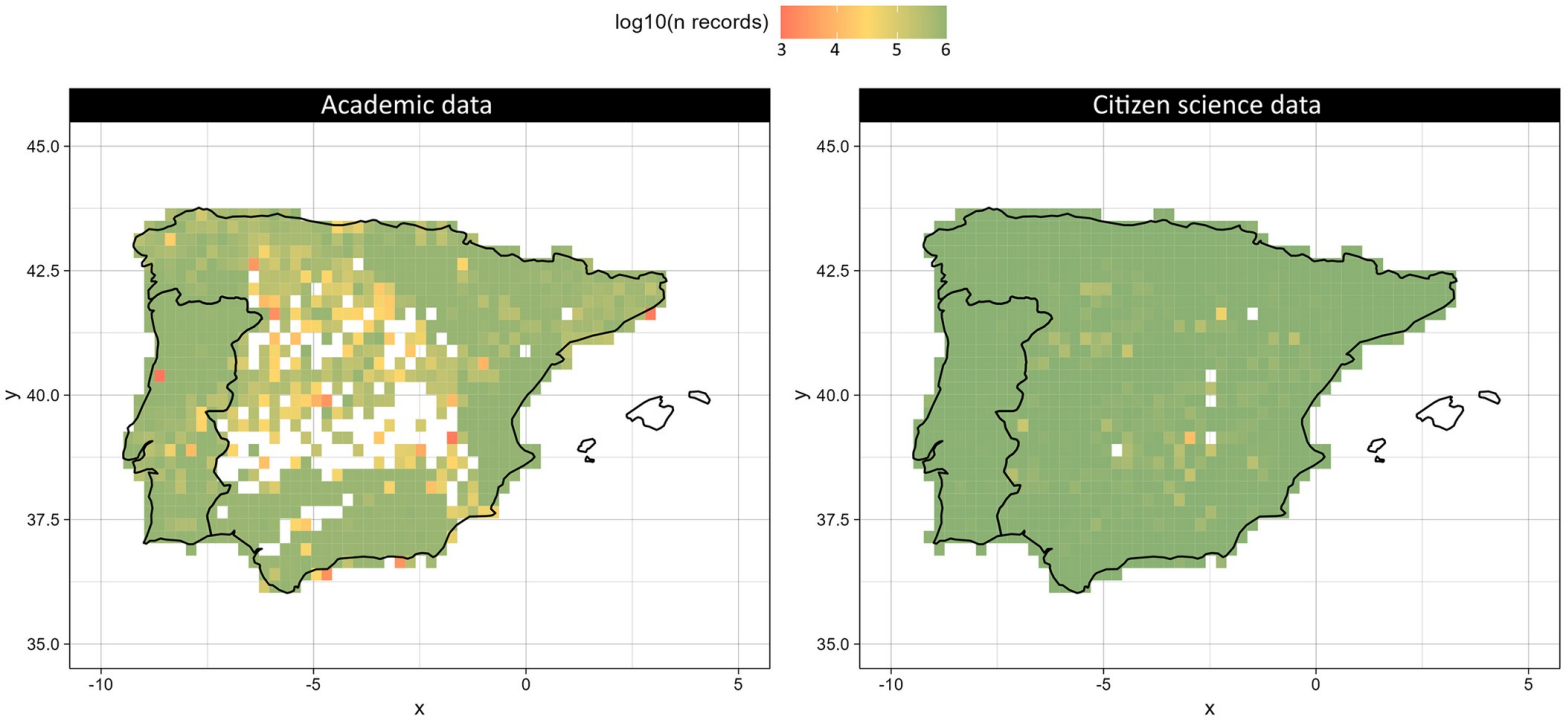
Representation of the log-transformed number of observations per grid cell at 0.25 degrees of resolution for (left) academic records and (right) citizen science records.

**Fig 6:**
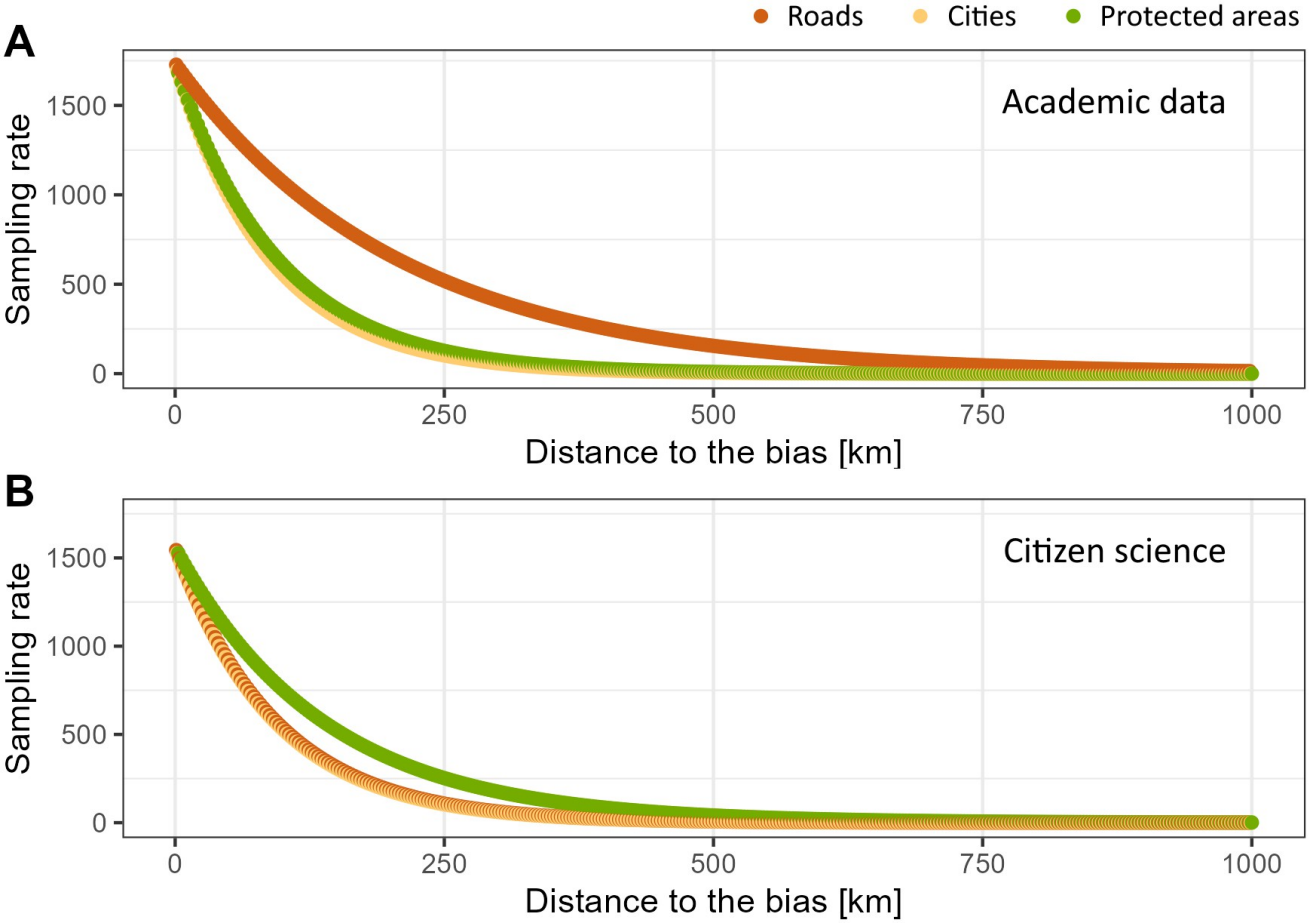
Relationship between data occurrence frequency and the proximity of roads (orange), cities (yellow) and protected natural areas (green) in academic data (top) and citizen science data (bottom).

Citizen Science data provide better coverage of the area of study than academic data (Fig 5). In the academic dataset, some areas remain unexplored (Fig 5A), whilst there are only five grid cells with no data when it comes to CS (Fig 5B). In addition to that, academic data seem to be biased towards protected natural areas and cities, with more records closer to these locations, whilst citizen science data are more biased towards cities and roads (Fig 6).

**Fig 7:**
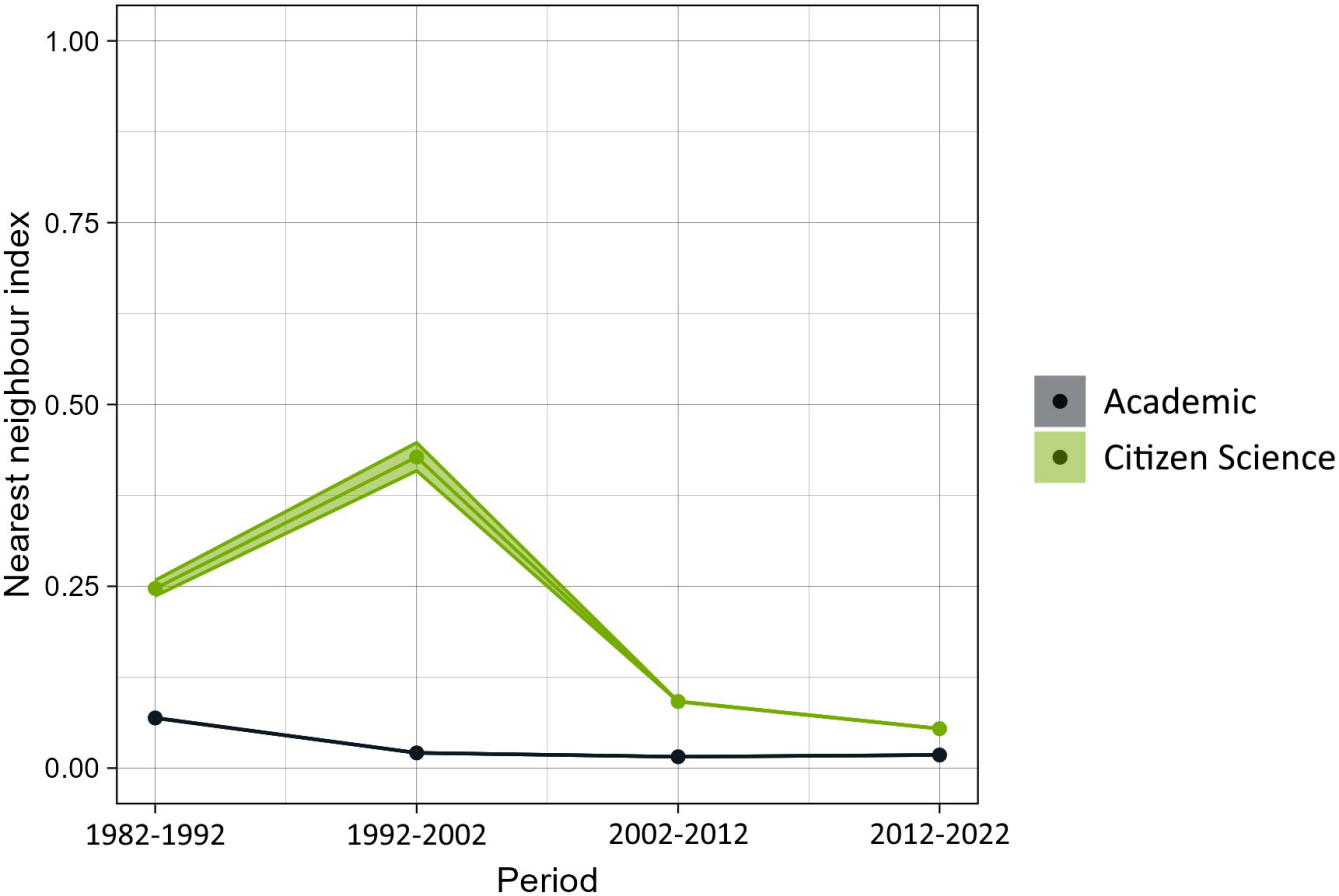
Spatial bias of academic records and citizen science records. Shaded regions indicate the 5th and the 95th percentile of the nearest neighbour index calculated over 99 iterations of random samples. The lower the index, the more clustered are the coordinates compared to a random sample of points. Each period corresponds to a 10-year timespan, starting in 1982 and ending in 2022. The first 10-year period for citizen science was excluded, as it had fewer than 100 data points.

Academic records have a consistently lower nearest neighbour index than CS records, meaning that they are more spatially clustered than CS records (Fig 7). Plus, this index does not change much across the different time periods for academic records, suggesting that spatial bias has been similar since 1982 in our dataset. On the other hand, CS data’s index has decreased since 1982, highlighting that the data have tended to cluster through time, slowly getting closer to the clustering level of academic data. Overall, the nearest neighbour index across all time periods is 0.01 for academic records and 0.05 for CS records.

### 3.3 Temporal bias

**Fig 8:**
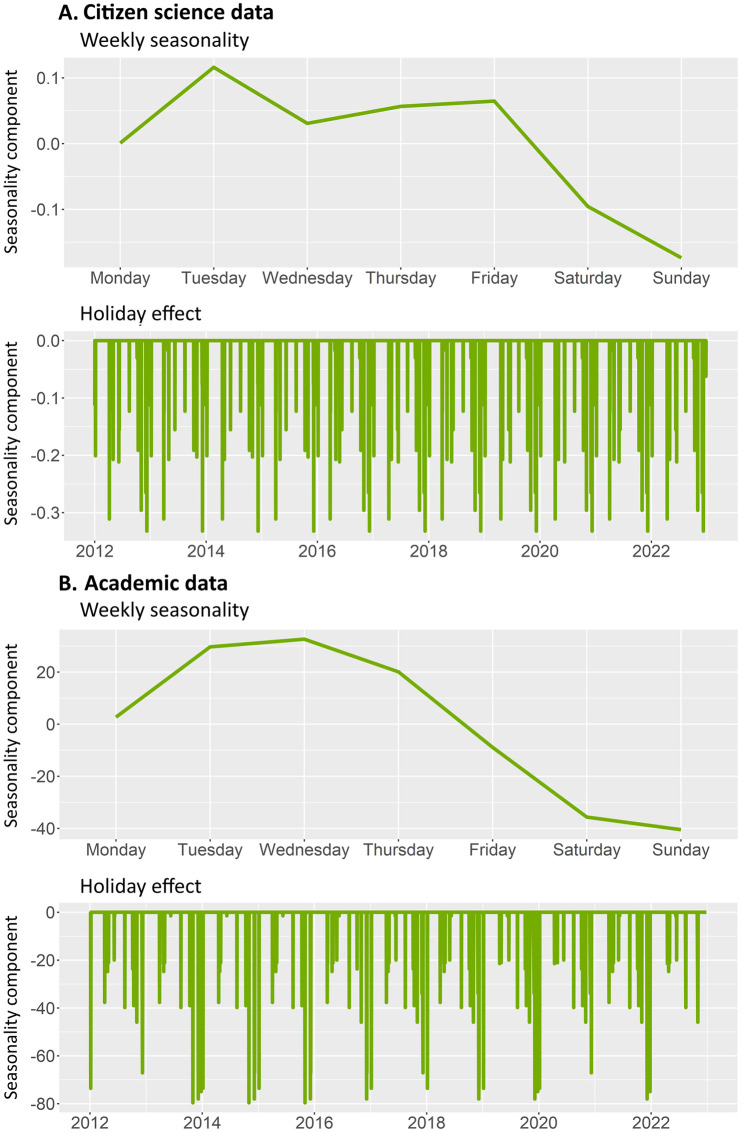
Weekly seasonality in data recording for academic data (A, top) and citizen science data (B, top), as well as how national holidays affect the temporal component of the data in Academic data (A, bottom) and citizen science data (B, bottom). Note that weekly seasonality had a bigger effect on academic data than on citizen science data (y axis on top graphs). The y axis represents the values that need to be added to or subtracted from the temporal trends on every specific day, owing to weekly seasonality.

Similar patterns were found for the weekly seasonality in academic and CS data (Fig 8). Although more observations are recorded during weekdays, progressively decreasing towards the weekend, CS data had a relatively higher recording rate on Fridays compared to academic data. Similarly, on Saturdays, observation rates were relatively higher in CS (Fig 8A), while in academic data both Saturdays and Sundays had the lowest recording rate (Fig 8B). Note that the axes on this figure have different scales. This is because the weekly seasonality component of academic data was higher than that of CS data. The y axis represents the values that need to be added to or subtracted from their respective temporal trends on every specific day, owing to weekly seasonality.

The holiday effect constantly repeats itself every year in both academic and CS data. In both datasets, all national holidays scored negatively against their respective temporal trends. This means that data are being collected at a lower rate during holidays than it would be expected by temporal trends. The days with the lowest observation rate were different in both datasets: All Saints’ Day (November), Spanish Constitution Day (December) and Christmas (December) for academic data, and *Inmaculada concepción* (December) and Good Friday (March-April) for CS data.

### 3.4 Environmental bias

**Fig 9:**
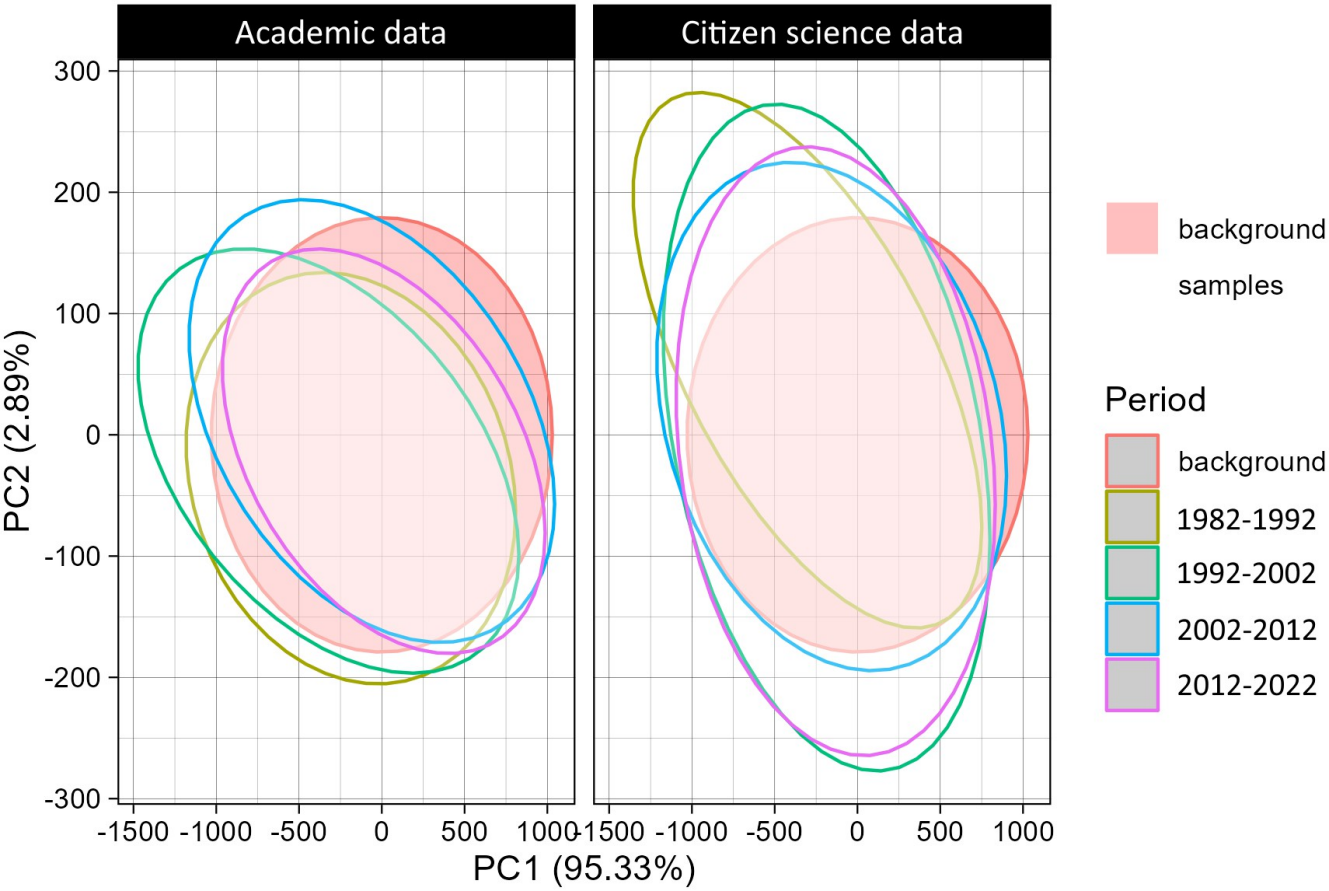
Bias in 2D environmental space for academic records (A) and citizen science data (B) across different 10-year time periods between 1982 and 2022. Background environmental space corresponds to 10,000 environmental points randomly selected across the study area.

The environmental space covered by academic records seems to shift more between 10-year periods than in the CS data. CS seems to be more constant across time periods (with the exception of 1982–1992, probably because there were no participatory platforms yet). These reduced differences indicate that the same environmental extent is covered regardless of the time period, suggesting a constant reduced environmental bias in CS data compared to academic records (Fig 9). In both CS and academic records, a similar extent of environmental space remains unexplored.

## 4. Discussion

The accuracy of citizen science data remains a subject of concern among scientists, with varying results reported so far. While some studies suggest variability in comparison to professionally collected data, others report comparable performance [31]. Nevertheless, it is crucial to approach the aggregation of citizen science quality assessments with caution, as this process often overlooks an evaluation of the quality of professional data and fails to consider variations within both citizen scientists and professionals [32].

The findings of this study offer valuable insights into taxonomic, spatial, temporal, and environmental biases present in both academic records and citizen science data related to insect observations. Neither citizen science data nor academic records are immune to biases, which can significantly impact the quality and reliability of observations [33]. Recent reviews caution against using professional data as the sole reference for evaluating citizen science data without concurrently assessing professional data quality. When precision assessments align for citizen scientists and professionals, they often indicate similar levels of precision. Additionally, some studies, while not directly assessing professional data quality, report anecdotal professional errors (e.g., [32]).

Despite the importance of recognizing these biases, quantitative comparisons between citizen science and academic approaches, like the one presented in this article, have been relatively rare in the literature [3]. Such comparisons offer detailed perspectives on the relative strengths and weaknesses of each data source, enabling researchers to make informed decisions about data selection for specific research objectives.

### 4.1 Taxonomic bias and user expertise and behaviour

The taxonomic bias observed in this study is largely attributed to variations in the distribution of observations and unique species among different insect orders. Lepidoptera is the most recorded insect group in both academic and citizen science data, showing a disproportionately high number of observations and species in both cases. For this insect group, CS data obtained a higher percentage of observations (27.58%), while academic records recorded more unique species (15.75%, Fig 1). Similarly, Coleoptera attracted considerable interest in academic records yet was not more popular than other orders in CS data. In fact, academic data recorded more beetle species than butterflies, probably because there are more described species of Coleoptera than Lepidoptera [34], or maybe because of the historical popularity of this group and their abundance in museum collections, where they may be more durable than butterflies. Conversely, other insect orders suffered from underrepresentation, particularly in citizen science data.

Lepidopterans are known for their charisma [35], and many academic and CS-based studies gathered in our dataset focused on this group, which explains the dominance of this insect order found in academic and CS data. Moreover, when recording moths, either at an academic or CS level, light traps are often used, yielding hundreds of records in a single night. This results in large datasets, which translate in an increase of the number of records for this insect order.

The potential biases introduced by the motivations and preferences of volunteer recorders are reported in previous works [14]. Volunteer recorders are often highly motivated by encounters with captivating wildlife, leading to a bias towards species that are easily observable and charismatic. This pattern is clearly reflected in our own findings, where Lepidoptera emerged as the most frequently recorded group, mirroring the observations made for iNaturalist [36]. Moreover, challenges in photographing and identifying very small organisms, such as some insects, can hinder their representation in records, as clear views of specific body structures or even dissection may be required for precise identification [36]. Nevertheless, although individual preferences for specific taxa may drive some of the taxonomic bias observed in participatory platforms, many volunteers are motivated by citizen science projects that direct them to make observations of a certain type of organisms. For instance, during the National Moth Week, people worldwide can participate by documenting moths in different habitats [37]. These factors collectively underscore the complex interplay between observer motivations, ease of observation, and resulting biases in citizen science and academic biodiversity datasets.

A potential reason why more unique species may be found in academic data compared to participatory platforms could be the participation of expert taxonomists that are able to assign precise identifications to difficult species groups. These challenging species may remain unidentified in participatory platforms, where experts for specific taxonomic groups are sometimes missing. Moreover, specimen collection, as required by most academic projects, make it possible to obtain identifications that may typically be impossible to get from sole pictures uploaded to a participatory platform. This would for instance be the case for cryptic species that require a stereomicroscope or even DNA barcoding, or those in which genitalia must be extracted and examined to get an accurate identification.

In CS, this taxonomic bias can in part be attributed to user recording behaviour. In Fig 3 we can see how certain users exhibit preferences for specific insect orders. Moreover, when pooling the data based on user engagement, leading contributors uploaded observations with patterns mirroring those of sporadic users and academic records, suggesting that the taxonomic bias is persistent regardless of user activity. This indicates that short-lived participatory platforms may be as successful as well-established platforms when it comes to recording biodiversity. However, when taking into consideration Shannon’s diversity, data gathered by sporadic users failed to reflect the taxonomic bias in academic records, which was contrastingly achieved in the data gathered by leading contributors. Nevertheless, short-lived participatory platforms focusing on a single insect order or species (e.g., invasive species) may be as efficient as well-established platforms where all biodiversity is recorded, although this merits more research. In any case, the strong correlation between the number of users and the number of observations in each order highlights the integral role of user behaviour in shaping the observed taxonomic biases. In fact, when comparing different platforms, taxonomic biases were conserved regardless of the amount of time these platforms had been running or their number of users (Fig 2).

The analysis of taxonomic bias based on species ranges revealed intriguing temporal trends. While citizen science data demonstrated an upward trend in reducing taxonomic bias and more equitably covering species’ distribution ranges, academic records exhibited a weakening trend. This suggests that despite academic data benefiting from a longer temporal span, their bias has not diminished as effectively as in citizen science data.

Regarding the expertise of the reporting users, the expertise bias could be one of the most critical and challenging to address. When it comes to experience in identification, many platforms do not just rely on the observer’s identification. They also incorporate an identification process to help verifying records, for instance, by considering the cumulative number of identifications by different users or by validating these records with experts. Due to the lack of consolidated theoretical framework and statistical tools, this type of bias has not been addressed in this paper, but we consider that it should be included in future studies. For citizen science, it is possible to develop taxon-specific reputation methods (e.g., [38]) that could provide the indicators to evaluate and correct this type of bias.

### 4.2 Spatial bias

Spatial bias is present in both academic and CS data, albeit in different ways. The overall nearest neighbour index across all time periods was 0.05 for CS and 0.01 for academic records. These values reflect more the situation in the last decades, for which there are substantially more data. Although the spatial clustering in CS data across time was lower than in academic data, this clustering tends to increase over time for CS. However, the use and popularity of CS has also been reported to increase [39]. CS data are prone to spatial biases [40], and these biases may increase together with the increase in popularity of participatory platforms. For instance, some participatory platforms organize events that focus user’s efforts in specific locations (e.g., Bioblitz). These events have gained popularity over the last decade, and may affect the spatial clustering of CS data to some extent. Another hypothesis for this increase in spatial clustering in CS data over time is that users in participatory platforms may upload old records retroactively, which may be fewer in number and more scattered spatially than what was being recorded back at that time in participatory platforms.

Citizen science data exhibited a higher spatial coverage than academic data, with fewer unexplored areas. Moreover, the spatial distribution of CS observations was less clustered (Fig 7). This may be because academic data tend to be focused on specific areas, especially when constant biodiversity monitoring is taking place. Contrastingly, citizen scientists can cover larger areas. The taxonomic bias index based on species distribution ranges is lower in academic data than in CS data (Fig 4), despite academic records having smaller spatial coverage than CS and potentially smaller species distribution areas. This suggests that academic data, besides covering less spatial range than CS data, are also over- or under sampling species.

Our analysis, consistent with prior research [4], underscores the presence of site-selection bias in biodiversity data, notably prominent in citizen science datasets. Volunteer recorders often gravitate towards locations close to their residences, protected areas, or regions with known species presence, potentially leading to skewed representations in the data. Although unstructured recording in biological and mass participation citizen science programs offers flexibility for expert naturalists, it carries the same susceptibility to selection bias as semi-structured recording [4]. Citizen science data are notably influenced by site-selection bias, driven by volunteers’ motivations to record diverse, threatened, or abundant species and hotspot areas [41]. Moreover, we recognize that site-selection bias is not unique to citizen science but also affects other major sources of biodiversity data, including museum collections, legacy sites, and resurveys [41]. This is consistent with our analysis on the impact of geographical features related to human accessibility. Academic data were biased towards protected natural areas, where sampling rates were higher and decreased faster with distance than in relation to other elements such as roads. Contrarily, in CS data, insect observations were more abundant closer to roads and cities.

In conclusion, our findings reveal different types of spatial biases in academic and CS data. We emphasize the potential of citizen science in providing broader and more evenly distributed spatial information, making it a valuable resource for biodiversity research. However, given the nature of the spatial biases in both data sources, it is important to develop strategies to mitigate their impact on ecological research. This can be achieved by employing the appropriate statistical methods, enhancing data collection protocols, and promoting targeted initiatives to gather data in underrepresented areas. By understanding and addressing these biases, researchers can more accurately assess biodiversity patterns and trends, ultimately informing conservation efforts and policy decisions. Combining academic and citizen science data may be the best alternative to achieve this, as their integration offers a more comprehensive perspective than relying solely on either dataset alone, especially since biases in these two types of data are not always the same. This integrative approach allows for a more complete picture of biodiversity, leveraging the strengths of both academic rigor and widespread public participation.

### 4.3 Temporal bias

Different temporal seasonalities were observed in both academic and CS data. The holiday effect and the day-of-the-week effect contributed significantly to temporal bias, shaping the timing of data collection by users. Although weekly seasonality was similar for both datasets, CS data showed a relatively higher recording rate on weekends compared to academic data, where recording rates were at their lowest. This may have implications for phenology studies that use CS data. For instance, the date of the first arrival of some migratory bird species is more likely to be reported over weekends than on weekdays [42], and thus it is recommended to include the day of the week in models that assess phenology using CS data. Holidays affected the temporal trends of both datasets negatively. In both cases, winter holidays scored the lowest recording rate. This is probably linked to a lower insect activity, besides also a lower user activity. The separation of these two confounding effects should be considered in further studies.

### 4.4 Environmental bias

There were differences between the coverage of environmental space across time between citizen science and academic records. Academic records exhibited greater shifts in environmental coverage across different time periods, while citizen science data remained relatively stable (Fig 9). There were also some environmental conditions that were unexplored for both datasets. These are probably some of the most inhospitable areas in the Iberian Peninsula. These results indicate the potential of citizen science data to provide more consistent and comprehensive coverage of environmental conditions. How climate change may create new environmental conditions and make others disappear, and how future academic and participatory data will cover such changes remains to be studied.

### 4.5 Future research and recommendations

In conclusion, this study shows the multifaceted nature of biases present in insect observation data collected from academic records and citizen science platforms in the Iberian Peninsula. User behaviour and preference, data collection methodologies, and external factors contribute to taxonomic, spatial, temporal, and environmental biases. The insights gained from this analysis emphasize the need for careful consideration of these biases when making use of biodiversity data from different sources. Moreover, the interplay between different types of bias (e.g., the correlation between environmental and spatial bias) should be explored further. We emphasize the importance of utilizing appropriate statistical approaches when analysing academic and citizen science datasets. This not only provides a more comprehensive understanding of data quality but also aligns with the recommendations made in previous research [4]. Additionally, the study highlights the potential of citizen science data to provide more comprehensive and evenly distributed species distribution data, making them a valuable resource for advancing biodiversity research and conservation efforts. Combining academic and citizen science data enhances our understanding of biodiversity, as their integration offers a more comprehensive perspective than relying solely on either dataset alone, especially since biases in these two types of data are not always the same.

## Supporting information

S1 Table Overview of the number of observations for academic and citizen science records, for each category of “basisOfRecord” in the Darwin Archive Core. (PDF)

S2 Table GBIF datasets which occurrence data contained entries labeled as “human observation” or “observation” according to the variable “basisOfRecord” from the GBIF Darwin Core Archive. Each dataset is sorted either as “academic” or “citizen science” in the Category column based on the nature of the data collection (i.e., data from literature or that was gathered following standardized protocols is considered “academic” and data collected without following any sampling protocol is considered “citizen science”). Note that some datasets were removed. Dataset descriptions were taken and/or adapted from their own metadata on GBIF. These datasets can be found in https://doi.org/10.15468/dl.mm74qg.
(PDF)
