# Supplementary material for: Revealing biases in insect observations: A comparative analysis between academic and citizen science data
Source: PLoS One. 2024 Jul 18;19(7):e0305757. doi: 10.1371/journal.pone.0305757 (PMC11257294; doi:10.1371/journal.pone.0305757)
Supplement: S1 Table — (PDF) [file pone.0305757.s001.pdf]

Supplementary material of: Revealing biases in insect observations: a comparative analysis between academic and citizen science data

Joan Díaz-Calafat[1], Sebastià Jaume-Ramis[2], Karen Soacha[3,4], Ana Álvarez[3], Jaume Piera[3]

[1] Southern Swedish Forest Research Centre, Swedish University of Agricultural Sciences, Box 190, 234 22 Lomma, Sweden.

[2] Mediterranean Parasitology and Ecoepidemiology research group. Department of Biology, University of the Balearic Islands, Palma, Spain.

[3] EMBIMOS research group, Institute of Marine Sciences (ICM), Spanish National Research Council (CSIC), Barcelona, Spain.

[4] Doctorate program in Information and Knowledge Society. Open University of Catalonia (UOC), Barcelona, Spain.

Abstract

Citizen Science is a powerful tool for biodiversity research, as it facilitates data recording at large scales that would otherwise be impossible to cover by standard academic research. Despite its benefits, the accuracy of citizen science data remains a subject of concern among scientists, with varying results reported so far. Neither citizen science data nor academic records are immune to biases, which can significantly impact the quality and reliability of observations. Here, using insects in the Iberian Peninsula as a case study, we compare data collected by participatory platforms to those obtained through academic research projects and assess their taxonomic, spatial, temporal, and environmental biases. Results show a prominent taxonomic bias in both academic and citizen science data, with certain insect orders receiving more attention than others. These taxonomic biases are conserved between different participatory platforms, as well as between groups of users with different levels of contribution performance. The biases captured by leading contributors in participatory platforms mirrored those of sporadic users and academic data. Citizen science data had a higher spatial coverage and less spatial clustering than academic data, showing also clearer trends in temporal seasonality. Environmental coverage over time was more stable in citizen science than in academic records. User behaviour, preference, taxonomical expertise, data collection methodologies and external factors may contribute to these biases. This study shows the multifaceted nature of biases present in academic records and citizen science platforms. The insights gained from this analysis emphasize the need for careful consideration of these biases when making use of biodiversity data from different sources. Combining academic and citizen science data enhances our understanding of biodiversity, as their integration offers a more comprehensive perspective than relying solely on either dataset alone, especially since biases in these two types of data are not always the same.

Key words: Participatory science, data quality, big data, biodiversity monitoring, sampling biases.

S1 Table. Overview of the number of observations for academic and citizen science records, for each category of "basisOfRecord" in the Darwin Archive Core.

	**Academic records**	**Citizen science records**
**Human observation**	527024	757124
**Machine observation**	2530	18
**Material citation**	842	0
**Material sample**	14976	0
**Observation**	178	0
**Occurrence**	11774	0
**Preserved specimen**	554370	0
