# Supplementary material for: Revealing biases in insect observations: A comparative analysis between academic and citizen science data
Source: PLoS One. 2024 Jul 18;19(7):e0305757. doi: 10.1371/journal.pone.0305757 (PMC11257294; doi:10.1371/journal.pone.0305757)
Supplement: S2 Table — Each dataset is sorted either as “academic” or “citizen science” in the Category column based on the nature of the data collection (i.e., data from literature or that was gathered following standardized protocols is considered “academic” and data collected without following any sampling protocol is considered “citizen science”). Note that some datasets were removed. Dataset descriptions were taken and/or adapted from their own metadata on GBIF. These datasets can be found in https://doi.org/10.15468/dl.mm74qg. (PDF) [file pone.0305757.s002.pdf]

Supplementary material of: Revealing biases in insect observations: a comparative analysis between academic and citizen science data

Joan Díaz-Calafat[1], Sebastià Jaume-Ramis[2], Karen Soacha[3,4], Ana Álvarez[3], Jaume Piera[3]

[1] Southern Swedish Forest Research Centre, Swedish University of Agricultural Sciences, Box 190, 234 22 Lomma, Sweden.

[2] Mediterranean Parasitology and Ecoepidemiology research group. Department of Biology, University of the Balearic Islands, Palma, Spain.

[3] EMBIMOS research group, Institute of Marine Sciences (ICM), Spanish National Research Council (CSIC), Barcelona, Spain.

[4] Doctorate program in Information and Knowledge Society. Open University of Catalonia (UOC), Barcelona, Spain.

Abstract

Citizen Science is a powerful tool for biodiversity research, as it facilitates data recording at large scales that would otherwise be impossible to cover by standard academic research. Despite its benefits, the accuracy of citizen science data remains a subject of concern among scientists, with varying results reported so far. Neither citizen science data nor academic records are immune to biases, which can significantly impact the quality and reliability of observations. Here, using insects in the Iberian Peninsula as a case study, we compare data collected by participatory platforms to those obtained through academic research projects and assess their taxonomic, spatial, temporal, and environmental biases. Results show a prominent taxonomic bias in both academic and citizen science data, with certain insect orders receiving more attention than others. These taxonomic biases are conserved between different participatory platforms, as well as between groups of users with different levels of contribution performance. The biases captured by leading contributors in participatory platforms mirrored those of sporadic users and academic data. Citizen science data had a higher spatial coverage and less spatial clustering than academic data, showing also clearer trends in temporal seasonality. Environmental coverage over time was more stable in citizen science than in academic records. User behaviour, preference, taxonomical expertise, data collection methodologies and external factors may contribute to these biases. This study shows the multifaceted nature of biases present in academic records and citizen science platforms. The insights gained from this analysis emphasize the need for careful consideration of these biases when making use of biodiversity data from different sources. Combining academic and citizen science data enhances our understanding of biodiversity, as their integration offers a more comprehensive perspective than relying solely on either dataset alone, especially since biases in these two types of data are not always the same.

Key words: Participatory science, data quality, big data, biodiversity monitoring, sampling biases.

S2 Table. GBIF datasets which occurrence data contained entries labeled as "human observation" or "observation" according to the variable "basisOfRecord" from the GBIF Darwin Core Archive. Each dataset is sorted either as "academic" or "citizen science" in the *Category* column based on the nature of the data collection (i.e. data from literature or that was gathered following standardized protocols is considered "academic" and data collected without following any sampling protocol is considered "citizen science"). Note that some datasets were removed. Dataset descriptions were taken and/or adapted from their own metadata on GBIF. These datasets can be found in https://doi.org/10.15468/dl.mm74qg.

Category	Dataset	Publisher	Description
Academic	Coleópteros del País Vasco - Sistema de Información de la Naturaleza de Euskadi	Basque Government	Compilation of the citations of beetles of the Cerambycidae, Lucanidae and Cetoniidae families for the Autonomous Community of the Basque Country existing in the scientific-technical literature and own records of the Aranzadi Society of Sciences.
Academic	Detección y distribución de coleópteros saproxílicos de interés comunitario en cuatro espacios de la red Natura 2000 del País Vasco	Basque Government	The objective of this work is to gradually add more information on the occurrence and distribution of saproxylic beetle species of community interest in RN2000 spaces that have been little or not prospected at all.
Academic	Programa de seguimiento de mariposas diurnas del País Vasco	Basque Government	Standardized transects of fixed length and width are conducted, noting down all butterfly species and their abundance. This methodology is repeated every two weeks during all the flying periods of butterflies.
Academic	Banco de Datos de la Biodiversidad de la Comunitat Valenciana	Biodiversity data bank of Generalitat Valenciana	This dataset aims to collect all the information on the location of species in the territory of the Valencian Community and its marine environment. The data comes from collections, bibliography and field work. All citations are georeferenced.
Academic		Biodiversity Data Journal	Compilation of biodiversity citations published in the Biodiversity Data Journal, hosting more than 600 datasets.
Citizen Science	Biodiversity4all Research-Grade Observations	BioDiversity4All	Observations from BioDiversity4All, an online social network of people sharing biodiversity information to help each other learn about nature. BioDiversity4All is maintained by Associação Biodiversidade Para Todos, and the Portuguese node of iNaturalist, a joint initiative of the California Academy of Sciences and the National Geographic Society.
Academic		Biologiezentrum Linz Oberoesterreich	Compilation of data from the museum Biologiezentrum Linz Oberoesterreich.

S2 Table. GBIF datasets which occurrence data contained entries labeled as "human observation" or "observation" according to the variable "basisOfRecord" from the GBIF Darwin Core Archive. Each dataset is sorted either as "academic" or "citizen science" in the *Category* column based on the nature of the data collection (i.e. data from literature or that was gathered following standardized protocols is considered "academic" and data collected without following any sampling protocol is considered "citizen science"). Note that some datasets were removed. Dataset descriptions were taken and/or adapted from their own metadata on GBIF. These datasets can be found in https://doi.org/10.15468/dl.mm74qg.

Category	Dataset	Publisher	Description
Academic		Centre d'estudis de la neu i de la muntanya d'Andorra (CENMA)	Compilation of biodiversity data collected from the CENMA, hosting 7 datasets.
Academic		CFE - Centre for Functional Ecology	Compilation of biodiversity data obtained from the CFE, hosting 2 datasets.
Academic	Portuguese Lepidoptera records compiled by Martin Corley's database	CIBIO (Research Center in Biodiversity and Genetic Resources) Portugal	This dataset consists of a collection of personal observations of the Lepidoptera expert, Martin Corley, in Portuguese territory. Contains information about the species observed, details of the methodology used, and the host where it was observed.
Academic	The Orthoptera of Castro Verde Special Protection Area (Southern Portugal)	CIBIO (Research Center in Biodiversity and Genetic Resources) Portugal	This dataset includes all the Orthoptera species recorded during a study performed in 2014 and 2015 mainly in Castro Verde Special Protection Area (SPA), southern Portugal, and is the first Orthoptera inventory conducted in the area. Orthopterans were visually recorded along transects placed in 61 sites.
Citizen Science	Mosquito Alert: Tiger Mosquito Dataset	CREAF - Centre de Recerca Ecològica i Aplicacions Forestals	Asian tiger mosquito (Aedes albopictus) observations done by citizen scientists using the Mosquito Alert app.
Academic		Czech University of Life Sciences Prague	Dataset from the Department of Forest Protection and Entomology at the Czech University of Life Sciences Prague
Academic		Danish Biodiversity Information Facility	Biodiversity datasets from the Danish Biodiversity Information Facility
Academic		Department of Forest Sciences	Biodiversity datasets obtained from the Department of Forest Science from the University of Helsinki.

S2 Table. GBIF datasets which occurrence data contained entries labeled as "human observation" or "observation" according to the variable "basisOfRecord" from the GBIF Darwin Core Archive. Each dataset is sorted either as "academic" or "citizen science" in the *Category* column based on the nature of the data collection (i.e. data from literature or that was gathered following standardized protocols is considered "academic" and data collected without following any sampling protocol is considered "citizen science"). Note that some datasets were removed. Dataset descriptions were taken and/or adapted from their own metadata on GBIF. These datasets can be found in https://doi.org/10.15468/dl.mm74qg.

Category	Dataset	Publisher	Description
Academic	DIBA-Parc Natural del Montseny	Diputació de Barcelona	Observations of fungi, lichens, flora and fauna from different sources in the Montseny Natural Park and Biosphere Reserve
Academic	EDP Foz Tua: Arthropoda – Environmental Impact Assessment [2006-2008]	EDP - Energias de Portugal	The dataset contains records of arthropods collected in the lower reaches of the Tua river, and included in the Environmental Impact Assessment of the Foz Tua Hydroelectric Dam, promoted by EDP – Energias de Portugal, S.A.
Academic	EDP Foz-Tua: Diurnal Butterflies (Lepidoptera) - Ecological Monitoring Program (2014-2017)	EDP - Energias de Portugal	The dataset contains records of Butterflies (Lepidoptera) collected in the lower valley of the River Tua, and included in the Ecological Monitoring Program (2011-2017) of the Foz Tua Hydroelectric Dam, promoted by EDP – Energias de Portugal, S.A. Data were collected between July 2014 and August 2017 during field visits to 33 sampling transects with favorable habitats on the area of the lower Tua valley.
Academic	EDP Foz-Tua: Dragonflies and Damselflies (Odonata) - Ecological Monitoring Program (2011-2017)	EDP - Energias de Portugal	The dataset contains records of Dragonflies and Damselflies (Odonata) collected in the lower valley of the River Tua, and included in the Ecological Monitoring Program (2011-2017) of the Foz Tua Hydroelectric Dam, promoted by EDP – Energias de Portugal, S.A. Data were collected between June 2011 and August 2017 during field visits to 33 sampling points with favorable habitats on the area of the lower Tua valley.
Academic	Long-term monitoring of roller dung beetles (Scarabaeinae) (abundance and distribution) in Doñana 2004-2012	Estación Biológica de Doñana (CSIC)	The monitoring of the roller dung-beetles (Scarabaeinidae) in Doñana, southwestern Spain, was initiated in 2004 as part of the Monitoring Program of Natural Resources and Processes. The aim was to obtain a temporal and continuous series of data in the abundance and distribution of two species (Scarabeus sacer and S. cicatricosus) present in the area. Data were recorded annually from 2004 to 2012 by members of the monitoring team which performed one sampling (between May and August) in different habitats (sand dunes, mediterranean shrublands, floodplain meadows, and marshlands).

S2 Table. GBIF datasets which occurrence data contained entries labeled as “human observation” or “observation” according to the variable “basisOfRecord” from the GBIF Darwin Core Archive. Each dataset is sorted either as “academic” or “citizen science” in the *Category* column based on the nature of the data collection (i.e. data from literature or that was gathered following standardized protocols is considered “academic” and data collected without following any sampling protocol is considered “citizen science”). Note that some datasets were removed. Dataset descriptions were taken and/or adapted from their own metadata on GBIF. These datasets can be found in https://doi.org/10.15468/dl.mm74qg.

Category	Dataset	Publisher	Description
Citizen Science	Estonian Naturalists' Society	Estonian Naturalists' Society	Citizen science biodiversity observations, various data from biodiversity monitoring projects, Atlas of Estonian Flora data etc
Academic	Database on Forest Disturbances in Europe	European Forest Institute	Historic information about forest damage caused by wind, fire, animals, and diseases. The DFDE has been elaborated by Alterra and European Forest Institute. The dataset shared here contains a subset of the data for occurrence of biotic agents only, i.e., mammals, insects and fungi.
Citizen Science		Field Study Group of the Dutch Mammal Society	Datasets of the Field Study Group, a subdivision of the Dutch Mammal Society, specializes in mammal surveys in the field throughout Europe. The study Group invests in new and innovative surveying methods.
Academic	Hatikka Observation Database	Finnish Biodiversity Information Facility	Digital field journal for Finnish amateur naturalists. It contains historical occurrence data for all taxon groups from Finland and several other countries.
Citizen Science		Fotografía y Biodiversidad	Datasets from citizen science as a source of information for the dissemination of knowledge and the conservation of nature.
Academic	Efectos del Cambio Ambiental en las comunidades de organismos de los ríos mediterráneos	Freshwater Ecology, Hydrology and Management (F.E.H.M.) Research Group - Universitat de Barcelona	Collection of aquatic macroinvertebrates of the studies of biodiversity monitoring and the ecological state of the rivers and streams. The study sites are within the protected areas of the Natural Parks of the Diputació de Barcelona and in other river reaches with reference conditions of the province of Barcelona. The main objective of the project is to examine whether global environmental changes are affecting the structure of these ecosystems.
Academic	Citacions biodiversitat Espais Naturals Protecció Especial	Generalitat de Catalunya	Set of biodiversity citations registered in the area of the Natural Areas of Special Protection of Catalonia, coming from the mobilization of citations generated in multiple works.
Academic	Haus der Natur Salzburg, Museum für Natur und Technik	Museum für Natur und Technik	Biodiversity datasets from the Haus der Natur, a natural history museum in the city of Salzburg.

S2 Table. GBIF datasets which occurrence data contained entries labeled as "human observation" or "observation" according to the variable "basisOfRecord" from the GBIF Darwin Core Archive. Each dataset is sorted either as "academic" or "citizen science" in the *Category* column based on the nature of the data collection (i.e. data from literature or that was gathered following standardized protocols is considered "academic" and data collected without following any sampling protocol is considered "citizen science"). Note that some datasets were removed. Dataset descriptions were taken and/or adapted from their own metadata on GBIF. These datasets can be found in https://doi.org/10.15468/dl.mm74qg.

**Category**	**Dataset**	**Publisher**	**Description**
Citizen Science	IASTracker. Invasive Alien Species database	IC5Team	IAS Tracker is a project to get locations of Invasive Alien Species (IAS) from common citizens.
Academic	Habitats Directive Portuguese Report: Species 2007-2012	ICNF - Instituto da Conservação da Natureza e das Florestas	This dataset is based on a National Report on the progress and implementation of the Habitats Directive in Portugal, concerning the 2007-2012 period. It originates from a project performed periodically (every 6 years) that aims to permit an adequate assessment of the progress, via the Natura 2000 Network, towards the goals of the Habitats Directive.
Academic	Habitats Directive Portuguese Report: Species 2013-2018	ICNF - Instituto da Conservação da Natureza e das Florestas	This dataset is based on a National Report on the progress and implementation of the Habitats Directive in Portugal, concerning the 2013-2018 period. It originates from a project performed periodically (every 6 years) that aims to permit an adequate assessment of the progress, via the Natura 2000 Network, towards the goals of the Habitats Directive.
Citizen Science	Data collected on citizen science web portal www.ornitho.cat	ICO-Institut Català d'Ornitologia (Catalan Ornithological Institute)	Collection of field observations shared by citizens. The observations are uploaded to GBIF at a resolution of 1x1 km, except for some species of conservation concern for which data are shown at 10x10 km resolution. Data refers to all vertebrates (except marine fishes), dragonflies, diurnal butterflies, crickets, grasshoppers, cicadas, saproxylic coleoptera, freshwater bivalves, decapods and orchids.
Citizen Science	iNaturalist Research-grade Observations	iNaturalist.org	Observations from iNaturalist.org, an online social network of people sharing biodiversity information to help each other learn about nature.
Academic		Leibniz Institute for the Analysis of Biodiversity Change (LIB)	More than 40 datasets from the Leibniz Institute for the Analysis of Biodiversity Change (LIB), formerly known as: Zoologisches Forschungsinstitut und Museum Alexander Koenig (ZFMK)
Academic		Marine Biology Laboratory	Dataset from the Marine Biology Laboratory

S2 Table. GBIF datasets which occurrence data contained entries labeled as "human observation" or "observation" according to the variable "basisOfRecord" from the GBIF Darwin Core Archive. Each dataset is sorted either as "academic" or "citizen science" in the *Category* column based on the nature of the data collection (i.e. data from literature or that was gathered following standardized protocols is considered "academic" and data collected without following any sampling protocol is considered "citizen science"). Note that some datasets were removed. Dataset descriptions were taken and/or adapted from their own metadata on GBIF. These datasets can be found in https://doi.org/10.15468/dl.mm74qg.

**Category**	**Dataset**	**Publisher**	**Description**
Academic		Museo Nacional de Ciencias Naturales (CSIC)	Dataset of the collection integrated by extant specimens mainly collected in Twentieth Century
Academic		Museum für Naturkunde Berlin	Datasets from the Museum für Naturkunde
Academic		National Museum of Natural History, Luxembourg	Datasets of occurrence data from the natural history collections and observation data managed by the National Museum of Natural History Luxembourg.
Citizen Science		naturgucker.de	Dataset from social network with several millions of observations of wild plants and animals
Academic		NeoBiota	Compilation of biodiversity articles published in NeoBiota journal.
Citizen Science		Observation.org	A citizen science organization collecting and presenting international plant, animal and other nature observations
Citizen Science		Odonata Central	This citizen science web site is focused on expanding our understanding of the distribution, biogeography, biodiversity, and identification of Odonata (dragonflies and damselflies) in the Western Hemisphere
Citizen Science	My naturesounds - nature observations with sound recordings	PlutoF	Sound-based taxon occurrences which have been provided by citizen scientists through PlutoF workbench and connected mobile application "Minu loodusheli" (My naturesound).
Citizen Science	Earth Guardians Weekly Feed	Questagame	QuestaGame is a free-to-play, mobile adventure game that gets players outdoors to discover, learn about and help protect biodiversity. Users can submit sightings of their local flora and fauna and receive points along with information about what they have found.

S2 Table. GBIF datasets which occurrence data contained entries labeled as “human observation” or “observation” according to the variable “basisOfRecord” from the GBIF Darwin Core Archive. Each dataset is sorted either as “academic” or “citizen science” in the *Category* column based on the nature of the data collection (i.e. data from literature or that was gathered following standardized protocols is considered “academic” and data collected without following any sampling protocol is considered “citizen science”). Note that some datasets were removed. Dataset descriptions were taken and/or adapted from their own metadata on GBIF. These datasets can be found in https://doi.org/10.15468/dl.mm74qg.

Category	Dataset	Publisher	Description
Academic	Dolichopodidae of Portugal - dataset 2009-2018	Research Institute for Nature and Forest (INBO)	A first comprehensive account on the dolichopodid fauna (Diptera: Dolichopodidae) of Portugal is presented here as the result of multiple surveys by primarily Portuguese researchers between 2009 and 2016.
Academic	Dung Beetles of the Western Palaearctic	Research Institute for Nature and Forest (INBO)	The dataset aggregates the results from a pan-European multi-site experiment, financially supported by the ALTER-Net consortium, Europe’s Ecosystem Research Network.
Academic	European stag beetle monitoring network: transect observations	Research Institute for Nature and Forest (INBO)	European stag beetle monitoring network: transect observations is an occurrence dataset published by the Research Institute of Nature and Forest (INBO). This dataset contains transect data for Lucanus cervus, collected using a standard protocol (European stag beetle monitoring network ESBMN).
Academic	Medetera species with multi-coloured eyes in southern Europe	Research Institute for Nature and Forest (INBO)	This dataset holds the records of 7 new soil-dwelling Medetera species with multi-coloured eyes in southern Europe.
Academic	Dataset of butterfly monitoring in Sierra Nevada (Spain)	Sierra Nevada Global Change Observatory. Andalusian Environmental Center	This data set comprises information collected in field about the monitoring of the butterfly communities of Sierra Nevada since 2008.
Academic		Sierra Nevada Global Change Observatory. Andalusian Environmental Center	Sierra Nevada Global Change Observatory is a long term monitoring programme to assess the effects of global change in the Sierra Nevada Biosphere Reserve (Spain).
Academic		Sociedade Galega de Historia Natural (SGHN)	Dataset compilation on biodiversity from Galicia, Spain.

S2 Table. GBIF datasets which occurrence data contained entries labeled as "human observation" or "observation" according to the variable "basisOfRecord" from the GBIF Darwin Core Archive. Each dataset is sorted either as "academic" or "citizen science" in the *Category* column based on the nature of the data collection (i.e. data from literature or that was gathered following standardized protocols is considered "academic" and data collected without following any sampling protocol is considered "citizen science"). Note that some datasets were removed. Dataset descriptions were taken and/or adapted from their own metadata on GBIF. These datasets can be found in https://doi.org/10.15468/dl.mm74qg.

Category	Dataset	Publisher	Description
Academic		Spanish Ministry for Ecological Transition and Demographic Challenge	Datasets from the Spanish Ministry for Ecological Transition and Demographic Challenge
Citizen Science		The Community Environmental Health Laboratory at MDI Biological Laboratory	The Community Environmental Health Laboratory at MDI Biological Laboratory in Bar Harbor, Maine engages community members as citizen scientists in collecting environmental data.
Academic	Tiroler Landesmuseum Ferdinandeum	Tiroler Landesmuseum Ferdinandeum	Not specified
Academic		UMS PatriNat (OFB-CNRS-MNHN), Paris	Biodiversity datasets of the UMS PatriNat (OFB-CNRS-MNHN), Paris
Academic	Azorean Biodiversity Portal	Universidade dos Açores	The Azorean Biodiversity Portal (ABP) is an e-infrastructure now associated with Portuguese PORBIOTA and LIFEWATCH.
Academic	Biodiversity data of lady beetles (Coleoptera: Coccinellidae) of the Azores archipelago (Portugal)	Universidade dos Açores	A recent review study from 2021 presents a comprehensive checklist of ladybirds of Portugal, including the Azores and Madeira Archipelagos.
Academic		Universitat de les Illes Balears	Several datasets obtained from biodiversity studies of the University of the Balearic Islands
Academic	Seguimiento y control científico de las capturas de Cerambyx cerdo en montes públicos de Mallorca	Universitat de les Illes Balears	Set of studies within the framework of a project called Monitoring and scientific control of the captures of Cerambyx cerdo in public oak forests of the Serra de Tramuntana Natural Area, Mallorca.

S2 Table. GBIF datasets which occurrence data contained entries labeled as “human observation” or “observation” according to the variable “basisOfRecord” from the GBIF Darwin Core Archive. Each dataset is sorted either as “academic” or “citizen science” in the *Category* column based on the nature of the data collection (i.e. data from literature or that was gathered following standardized protocols is considered “academic” and data collected without following any sampling protocol is considered “citizen science”). Note that some datasets were removed. Dataset descriptions were taken and/or adapted from their own metadata on GBIF. These datasets can be found in https://doi.org/10.15468/dl.mm74qg.

Category	Dataset	Publisher	Description
Citizen Science	eButterfly Surveys	Vermont Center for Ecostudies	Dataset of citizen observation of butterflies
Citizen Science	eButterfly Species List	Vermont Center for Ecostudies	Dataset of citizen observation of butterflies
Citizen Science		Xeno-canto Foundation for Nature Sounds	Datasets of all bird sounds, meaning all taxa, to subspecies level, their complete repertoire, all of the geographic variability, at all stages of development
Citizen Science	Portuguese Moth Recording Scheme	GBIF Portugal	This resource includes the records of macro-moths produced by the Portuguese Moth Recording Scheme called "Rede de Estações de Borboletas Nocturnas" (REBN).
Academic	The Albert J. Cook Arthropod Research Collection	A. J. Cook Arthropod Research Collection	Dataset of the arthropod collection of Professor Albert J. Cook
Academic		Adam Mickiewicz University in Poznan	Dataset of the museum collection of The Faculty of Biology, Adam Mickiewicz University in Poznań
Academic	Canadian National Collection of Insects, Arachnids, and Nematodes (CNC)	Agriculture and Agri-Food Canada	The Canadian National Collection of Insects, Arachnids and Nematodes (CNC) of Agriculture and Agri-Food Canada is one of the five largest collections of its kind in the world. It contains over 17 million specimens
Academic	Checklist of the Crambidae of the Region of Murcia (Spain) with new records (Lepidoptera: Pyraloidea	Animal Biology Section, Zoology and Phisic Antropology Department, Murcia University	A list of Crambidae moths (Lepidoptera: Pyraloidea) is synthesised based on an examination of museum specimens, published records and new samples.
Academic	Checklist of the Pyralidae of the Region of Murcia (Spain) with new records (Lepidoptera: Pyraloidea	Animal Biology Section, Zoology and Phisic Antropology Department, Murcia University	A list of Pyralidae moths (Lepidoptera: Pyraloidea) is synthesised based on an examination of museum specimens, published records and new samples.

S2 Table. GBIF datasets which occurrence data contained entries labeled as “human observation” or “observation” according to the variable “basisOfRecord” from the GBIF Darwin Core Archive. Each dataset is sorted either as “academic” or “citizen science” in the *Category* column based on the nature of the data collection (i.e. data from literature or that was gathered following standardized protocols is considered “academic” and data collected without following any sampling protocol is considered “citizen science”). Note that some datasets were removed. Dataset descriptions were taken and/or adapted from their own metadata on GBIF. These datasets can be found in https://doi.org/10.15468/dl.mm74qg.

Category	Dataset	Publisher	Description
Academic		Arizona State University Biocollections	The Arizona State University Biocollections comprise two sets of distinct collections: (1) the Arizona State University Natural History Collections - currently with nine collections focused on documenting Greater Sonoran and New World biodiversity; and (2) the NEON Biorepository at Arizona State University, with a unique constellation of organismal and environmental samples generated in the context of monitoring and forecasting long-term ecological change in the North American subcontinent, including Alaska, Hawaii, and Puerto Rico.
Academic		Australian Museum	Biodiversity datasets from the natural history collections available through the Atlas of Living Australia.
Academic	Coleópteros del País Vasco - Sistema de Información de la Naturaleza de Euskadi	Basque Government	Compilation of the citations of beetles of the Cerambycidae, Lucanidae and Cetoniidae families for the Autonomous Community of the Basque Country existing in the scientific-technical literature and own records of the Aranzadi Society of Sciences.
Academic		Belgian Biodiversity Platform	Dataset compilation from the Belgian Biodiversity Platform
Academic		Berkeley Natural History Museums	Berkeley's biodiversity repositories & informatics services
Academic	Biofokus	Biofokus	Biodiversity dataset that contributes to the knowledge-based management of Norwegian nature.
Academic		Botanic Garden and Botanical Museum Berlin	Biodiversity datasets from the Botanic Garden and Botanical Museum Berlin

S2 Table. GBIF datasets which occurrence data contained entries labeled as "human observation" or "observation" according to the variable "basisOfRecord" from the GBIF Darwin Core Archive. Each dataset is sorted either as "academic" or "citizen science" in the *Category* column based on the nature of the data collection (i.e. data from literature or that was gathered following standardized protocols is considered "academic" and data collected without following any sampling protocol is considered "citizen science"). Note that some datasets were removed. Dataset descriptions were taken and/or adapted from their own metadata on GBIF. These datasets can be found in https://doi.org/10.15468/dl.mm74qg.

Category	Dataset	Publisher	Description
Academic	Brigham Young University Arthropod Museum	Brigham Young University, Arthropod Collection	Dataset with approximately 2,000,000 specimens, mostly insects. Although worldwide in coverage, an emphasis is on western North America
Academic		California Academy of Sciences	Biodiversity datasets from the California Academy of Sciences
Academic	Laboratorio de Entomología y Control de Plagas del Instituto Cavanilles de Biodiversidad y Biología Evolutiva de la Universidad de Valencia: ENV	Cavanilles Institute of Biodiversity and Evolutionary Biology, University of Valencia	The data shown in this database are the property of the Entomology and Pest Control Laboratory of the University of Valencia. Each data item represents an arthropod or part thereof, belonging to one of the following orders: Hymenoptera, Coleoptera, Lepidoptera, Orthoptera, Hemiptera, Diptera, Neuroptera, Dyctioptera and Odonata. The collection is made up of specimens collected and/or worked on in the Laboratory itself and others from collections donated to the laboratory.
Academic		Centro de Estudios Avanzados de Blanes (CSIC)	Biodiversity data from marine and continental water ecosystems.
Academic	The InBIO Barcoding Initiative Database: DNA barcodes of Portuguese Diptera 02 - Limoniidae, Pediciidae and Tipulidae	CIBIO (Research Center in Biodiversity and Genetic Resources) Portugal	The dataset contains 412 records of Diptera species from the families Limoniidae, Pediciidae and Tipulidae, collected between 2003 to 2019 in Portugal, including the Azores and Madeira archipelagos (Ferreira et al., 2021; Oosterbroek et al., 2020; Starý, 2014).
Academic	The InBIO Barcoding Initiative Database: Diptera 03 - Further records on Portuguese	CIBIO (Research Center in Biodiversity and Genetic Resources) Portugal	The dataset contains 219 entries with 241 records of specimens from the families Limoniidae, Pediciidae and Tipulidae, collected between 1985 to 2019 in continental Portugal (Oosterbroek et al., 2020, part).

S2 Table. GBIF datasets which occurrence data contained entries labeled as "human observation" or "observation" according to the variable "basisOfRecord" from the GBIF Darwin Core Archive. Each dataset is sorted either as "academic" or "citizen science" in the *Category* column based on the nature of the data collection (i.e. data from literature or that was gathered following standardized protocols is considered "academic" and data collected without following any sampling protocol is considered "citizen science"). Note that some datasets were removed. Dataset descriptions were taken and/or adapted from their own metadata on GBIF. These datasets can be found in https://doi.org/10.15468/dl.mm74qg.

Category	Dataset	Publisher	Description
	Limoniidae, Pediciidae and Tipulidae		
Academic	The InBIO Barcoding Initiative Database: Blattodea 01	CIBIO (Research Center in Biodiversity and Genetic Resources) Portugal	The dataset contains 38 records of Blattodea collected from 2005 to 2019 in continental Portugal. Specimens were identified to species level, except for a single record identified to genus level. Of the eight species in the dataset, seven are cockroaches and one is a termite.
Academic	The InBIO Barcoding Initiative Database: Borkhausenia crimnodes Meyrick, 1912 (Lepidoptera, Oecophoridae), a southern hemisphere species resident in Portugal	CIBIO (Research Center in Biodiversity and Genetic Resources) Portugal	The dataset contains one record of the species Borkhausenia crimnodes (Lepidoptera, Oecophoridae) collected in continental Portugal in 2014.
Academic	The InBIO Barcoding Initiative Database: Taxonomic notes on Portuguese Microlepidoptera II. Cochylimorpha punctiferana (Ragonot, 1881)	CIBIO (Research Center in Biodiversity and Genetic Resources) Portugal	The dataset contains one record of the species Cochylimorpha punctiferana (Ragonot, 1881) (Lepidoptera, Tortricidae) collected in continental Portugal in 2016.
Academic	The InBIO Barcoding Initiative Database: Depressaria infernella Corley & Buchner, a new Iberian species of the Depressaria douglasella group	CIBIO (Research Center in Biodiversity and Genetic Resources) Portugal	The dataset contains two records of moth specimens (Lepidoptera, Depressariidae) collected in 2011 and 2015 in continental Portugal. The study of these specimens resulted in the description of a new Portuguese endemic species, Depressaria infernella Corley & Buchner, 2019 based on differences found in the male and female genitalia and DNA barcodes in Corley et al. (2019).

S2 Table. GBIF datasets which occurrence data contained entries labeled as "human observation" or "observation" according to the variable "basisOfRecord" from the GBIF Darwin Core Archive. Each dataset is sorted either as "academic" or "citizen science" in the *Category* column based on the nature of the data collection (i.e. data from literature or that was gathered following standardized protocols is considered "academic" and data collected without following any sampling protocol is considered "citizen science"). Note that some datasets were removed. Dataset descriptions were taken and/or adapted from their own metadata on GBIF. These datasets can be found in https://doi.org/10.15468/dl.mm74qg.

Category	Dataset	Publisher	Description
Academic	The InBIO Barcoding Initiative Database: Diptera 01	CIBIO (Research Center in Biodiversity and Genetic Resources) Portugal	The dataset contains 203 records of Diptera species collected from 2014 to 2018 in continental Portugal (Ferreira et al., 2020).
Academic	The InBIO Barcoding Initiative Database: Hemiptera 01	CIBIO (Research Center in Biodiversity and Genetic Resources) Portugal	The dataset contains 135 records of Hemiptera species collected from 2015 to 2019 in continental Portugal (Sousa et al., 2021).
Academic	The InBIO Barcoding Initiative Database: Portuguese Stag beetles (Coleoptera, Lucanidae)	CIBIO (Research Center in Biodiversity and Genetic Resources) Portugal	The dataset contains 11 records of Stag beetle (Lucanidae) species collected from 2015 to 2019 in continental Portugal.
Academic	The InBIO Barcoding Initiative Database: Portuguese Scorpionflies (Mecoptera, Panorpidae)	CIBIO (Research Center in Biodiversity and Genetic Resources) Portugal	The dataset contains 13 records of Scorpionflies (Panorpidae) species collected from 2008 to 2019 in continental Portugal.
Academic	The InBIO Barcoding Initiative Database: Lacewings (Neuroptera) 01	CIBIO (Research Center in Biodiversity and Genetic Resources) Portugal	The dataset contains 234 records of Lacewings (Neuroptera) species collected from 2006 to 2019 in continental Portugal (Oliveira et al., 2021).
Academic	The InBIO Barcoding Initiative Database: Lacewings (Neuroptera) 02	CIBIO (Research Center in Biodiversity and Genetic Resources) Portugal	The dataset contains 349 records of Lacewings (Neuroptera) species collected from 2006 to 2019 in continental Portugal.
Academic	The InBIO Barcoding Initiative Database: Wesmaelius (Kimninsia) nervosus (Neuroptera,	CIBIO (Research Center in Biodiversity and Genetic Resources) Portugal	The dataset contains one record of the species Wesmaelius subnebulosus (Stephens, 1836) (Neuroptera, Hemerobiidae), a brown lacewing species, collected

S2 Table. GBIF datasets which occurrence data contained entries labeled as "human observation" or "observation" according to the variable "basisOfRecord" from the GBIF Darwin Core Archive. Each dataset is sorted either as "academic" or "citizen science" in the *Category* column based on the nature of the data collection (i.e. data from literature or that was gathered following standardized protocols is considered "academic" and data collected without following any sampling protocol is considered "citizen science"). Note that some datasets were removed. Dataset descriptions were taken and/or adapted from their own metadata on GBIF. These datasets can be found in https://doi.org/10.15468/dl.mm74qg.

Category	Dataset	Publisher	Description
	Hemerobiidae): a new species of brown lacewing for the Portuguese fauna		in continental Portugal in 2017, and first recorded for the country in Oliveira and Ferreira (2020).
Academic	The InBIO Barcoding Initiative Database: Portuguese Snakeflies (Raphidioptera)	CIBIO (Research Center in Biodiversity and Genetic Resources) Portugal	The dataset contains 8 records of Snakeflies (Raphidioptera) species collected from 2016 to 2019 in continental Portugal.
Academic	The InBIO Barcoding Initiative Database: DNA barcodes of Iberian Trichoptera 01	CIBIO (Research Center in Biodiversity and Genetic Resources) Portugal	The dataset contains 438 records of Trichoptera species from 22 of the 23 families known from the Iberian Peninsula. Specimens were collected between 1975 to 2018 in Portugal, Spain and France (Paupério et al., 2023).
Academic	The InBIO Barcoding Initiative Database: A taxonomic revision of the Western Palaearctic genus Cacochroa Heinemann, 1870 (Lepidoptera, Depressariidae)	CIBIO (Research Center in Biodiversity and Genetic Resources) Portugal	The dataset contains 11 records of moth specimens (Lepidoptera, Depressariidae) collected from 1988 to 2016 in seven South European and North African countries.
Academic	Portuguese Lepidoptera records compiled by Martin Corley's database	CIBIO (Research Center in Biodiversity and Genetic Resources) Portugal	This dataset consists of a collection of personal observations of the Lepidoptera expert, Martin Corley, in Portuguese territory.
Academic	The Orthoptera of Castro Verde Special Protection Area (Southern Portugal)	CIBIO (Research Center in Biodiversity and Genetic Resources) Portugal	This dataset includes all the Orthoptera species recorded during a study performed in 2014 and 2015 mainly in Castro Verde Special Protection Area (SPA), southern Portugal, and is the first Orthoptera inventory conducted in the area.

S2 Table. GBIF datasets which occurrence data contained entries labeled as "human observation" or "observation" according to the variable "basisOfRecord" from the GBIF Darwin Core Archive. Each dataset is sorted either as "academic" or "citizen science" in the *Category* column based on the nature of the data collection (i.e. data from literature or that was gathered following standardized protocols is considered "academic" and data collected without following any sampling protocol is considered "citizen science"). Note that some datasets were removed. Dataset descriptions were taken and/or adapted from their own metadata on GBIF. These datasets can be found in https://doi.org/10.15468/dl.mm74qg.

Category	Dataset	Publisher	Description
Academic		Clemson University Arthropod Collection	The Collection consists of approximately 1.3 million specimens from Classes Insecta, Arachnida, Branchipoda, Copepoda, Diplopoda, and Chilopoda.
Academic		Colecciones naturales de la Asociación BIGA para el estudio del patrimonio natural de Galicia: FBIGA y ABIGA.	Dataset collection from Galicia, Spain
Academic		Colorado State University, C.P. Gillette Museum of Arthropod Diversity	Datasets of the C. P. Gillette Museum of Arthropod Diversity houses more than three and a half million specimens and has excellent representation of most orders of insects especially with a strong coverage of Rocky Mountain species, but also southwestern species.
Academic		Commonwealth Scientific and Industrial Research Organisation	Datasets of CSIRO, custodian of several collections of animal and plant specimens that contribute to national and international biological knowledge.
Academic	Museu Darder de Banyoles	Darder Natural History Museum of Banyoles	Dataset of the collection of the Darder Natural History Museum
Academic		Denver Museum of Nature & Science	Biodiversity datasets
Academic		Dep. Biology, Univ. Autónoma de Madrid	Biodiversity data from the Laboratory of Entomology of the Dept. Biology, Section Zoology, Univ. Autónoma de Madrid.
Academic		Department of Animal Biology, Faculty of	Dataset from the department of Animal Biology, University of La Laguna

S2 Table. GBIF datasets which occurrence data contained entries labeled as "human observation" or "observation" according to the variable "basisOfRecord" from the GBIF Darwin Core Archive. Each dataset is sorted either as "academic" or "citizen science" in the *Category* column based on the nature of the data collection (i.e. data from literature or that was gathered following standardized protocols is considered "academic" and data collected without following any sampling protocol is considered "citizen science"). Note that some datasets were removed. Dataset descriptions were taken and/or adapted from their own metadata on GBIF. These datasets can be found in https://doi.org/10.15468/dl.mm74qg.

Category	Dataset	Publisher	Description
		Biology, University of La Laguna	
Academic		Department of Forest Science	Datasets from the Department of Forest Sciences, University of Helsinki
Academic		Department of Organisms and Systems Biology. University of Oviedo	Datasets of the biodiversity collections (Herbarium FCO, Collection BOS) of the University of Oviedo, Spain.
Academic		Dept. of Zoology, Faculty of Science, University of Granada	Datasets from the Dept. of Zoology, Faculty of Science, University of Granada
Academic	Prey identification of free-ranging domestic cats (Felis catus) from rural and natural areas of Spain through scat analysis	Estación Biológica de Doñana (CSIC)	This dataset includes information on the prey consumed by free-ranging domestic cats with different care/nourishment conditions, obtained from the analysis of scats.
Academic		Estonian Museum of Natural History	Datasets of the collection of the Estonian Museum of Natural History
Academic		Estonian University of Life Sciences	Datasets of nature collections with ca 1,3 million specimen.

S2 Table. GBIF datasets which occurrence data contained entries labeled as “human observation” or “observation” according to the variable “basisOfRecord” from the GBIF Darwin Core Archive. Each dataset is sorted either as “academic” or “citizen science” in the *Category* column based on the nature of the data collection (i.e. data from literature or that was gathered following standardized protocols is considered “academic” and data collected without following any sampling protocol is considered “citizen science”). Note that some datasets were removed. Dataset descriptions were taken and/or adapted from their own metadata on GBIF. These datasets can be found in https://doi.org/10.15468/dl.mm74qg.

Category	Dataset	Publisher	Description
Academic		European Nucleotide Archive (EMBL-EBI)	The European Nucleotide Archive, maintained by the European Molecular Biology Laboratory’s European Bioinformatics Institute, captures and presents globally comprehensive sequence data as part of the International Nucleotide Sequence Database Collaboration. Data provided to GBIF include geotagged environmental sequences with user-provided taxonomic identifications.
Academic		Flanders Marine Institute	Datasets from the Flanders Marine Institute.
Academic		Illinois Natural History Survey	The INHS Insect Collection, which comprises ca. 7 million prepared specimens as well as non insect arthropods (e.g., arachnids and myriapods) and miscellaneous invertebrates (bryozoans), is one of the largest and oldest entomological collections in North America.
Academic	Insectarium de Montréal (IMQC)	Insectarium de Montréal	The Insectarium’s scientific collections are composed of more than 225,000 insect specimens and include major specialized collections for certain taxa.
Academic		Institute of Biodiversity, Animal Health and Comparative Medicine, College of Medical, Veterinary and Life Sciences, University of Glasgow	Datasets from the Institute of Biodiversity of the University of Gasgow.

S2 Table. GBIF datasets which occurrence data contained entries labeled as “human observation” or “observation” according to the variable “basisOfRecord” from the GBIF Darwin Core Archive. Each dataset is sorted either as “academic” or “citizen science” in the *Category* column based on the nature of the data collection (i.e. data from literature or that was gathered following standardized protocols is considered “academic” and data collected without following any sampling protocol is considered “citizen science”). Note that some datasets were removed. Dataset descriptions were taken and/or adapted from their own metadata on GBIF. These datasets can be found in https://doi.org/10.15468/dl.mm74qg.

**Category**	**Dataset**	**Publisher**	**Description**
Academic	IICT Tabanidae Collection	Instituto de Investigação Científica Tropical	The Instituto de Investigação Científica Tropical hosts a collection of horseflies (Order Diptera, Family Tabanidae) collected by J. A. Travassos Santos Dias and other researchers, mainly in Portugal and during several expeditions to Angola, Mozambique and São Tomé and Príncipe in the decades of 1940 to 1980. The collection consists of 1319 specimens and includes type specimens of species described by Travassos Santos Dias, as well as species with little distribution data available online.
Academic	Institut Mediterrani d'Estudis Avançats (CSIC-UIB): IMEDEA-INSECTA	Instituto Mediterráneo de Estudios Avanzados (CSIC)	IMEDEA-INSECTA includes more than 10.000 records of the insect collection that are found at the Institut Mediterrani d'Estudis Avançats (CSIC-UIB) located in Esporles (Mallorca, España). The database includes, on one hand, specimens that were collected during the PhD thesis of Dr. Miquel Palmer (all these in the order Coleoptera) and, on the other hand, specimens collected during different research projects on pollinaion at the Terrestrial Ecology Laboratory, of IMEDEA.
Academic		Jyväskylä University Museum - The Section of Natural Sciences	Datasets from the Jyväskylä University Museum
Academic	KwaZulu-Natal Museum-Entomology-collection	KwaZulu-Natal Museum	The collection of flies contains more than 1275 primary types and 5474 secondary types. It is housed in almost 1000 wooden insect drawers together with an alcohol and slide collection. The Entomology collection also includes several smaller collections including Heteroptera and Mecoptera.
Academic	Lyman Entomological Museum (LEMQ)	McGill University	Insect and arachnid specimens deposited in the Lyman Entomological Museum, McGill University.

S2 Table. GBIF datasets which occurrence data contained entries labeled as "human observation" or "observation" according to the variable "basisOfRecord" from the GBIF Darwin Core Archive. Each dataset is sorted either as "academic" or "citizen science" in the *Category* column based on the nature of the data collection (i.e. data from literature or that was gathered following standardized protocols is considered "academic" and data collected without following any sampling protocol is considered "citizen science"). Note that some datasets were removed. Dataset descriptions were taken and/or adapted from their own metadata on GBIF. These datasets can be found in https://doi.org/10.15468/dl.mm74qg.

**Category**	**Dataset**	**Publisher**	**Description**
Academic		MNHN - Museum national d'Histoire naturelle	Datasets of the French MNHN.
Academic	Dermaptera	Musée des Confluences	This dataset is the databased part of Dermaptera from the entomological collections of the Musée des Confluences (Lyon). It comprises 488 specimens belonging to different collections (Harold Labrique: missions and collections in Morocco, 2004-2018, René Pupier, Jean David and many others).
Academic	Phengaris	Musée des Confluences	This dataset is the databased part of Phengaris (ex Maculinea) from the entomological collections of the Musée des Confluences (Lyon). It comprises 1439 specimens belonging to 23 collections (Claude Dufay, André Primot, Jacques Baraud, Alain Crosson du Cormier, François Moulignier, René Martin, Georges Brunier, Pierre Réal, Etienne Berjot, Emile Roman etc.).
Academic	Museu de Ciències Naturals de Barcelona: MCNB-Art	Museu de Ciències Naturals de Barcelona	The Arthropod Collection is the most numerous and diverse of all the Museum's collections and houses over half the total number of its specimens. The exact number of specimens is difficult to estimate but lies somewhere in the range 1,750,000–1,900,000. Most of the orders of all the different subphylums (Chelicerata, Crustacea, Uniramia) that constitute the Arthropoda are represented, although the Hexapoda are the best represented group given the high number of insect specimens.
Academic	New records for crown wasps in Europe (Hymenoptera, Stephanidae)	Museu de Ciències Naturals de Barcelona	New occurrence records for two species of Hymenoptera Stephanidae are provided from several states of Europe.

S2 Table. GBIF datasets which occurrence data contained entries labeled as "human observation" or "observation" according to the variable "basisOfRecord" from the GBIF Darwin Core Archive. Each dataset is sorted either as "academic" or "citizen science" in the *Category* column based on the nature of the data collection (i.e. data from literature or that was gathered following standardized protocols is considered "academic" and data collected without following any sampling protocol is considered "citizen science"). Note that some datasets were removed. Dataset descriptions were taken and/or adapted from their own metadata on GBIF. These datasets can be found in https://doi.org/10.15468/dl.mm74qg.

Category	Dataset	Publisher	Description
Academic	Insect Collection from the Museu Nacional de História Natural e da Ciência, Universidade de Lisboa, Portugal	Museu Nacional de História Natural e da Ciência	The Entomological collection of the National Museum of Natural History and Science, of the University of Lisbon, Portugal, comprises over 40000 records, corresponding to about 70450 specimens.
Academic		Muséum d'histoire naturelle de la Ville de Genève - MHNG	Datasets from the MHNG, Switzerland.
Academic		Museum of Biological Diversity, The Ohio State University	Datasets from the Museum of Biological Diversity, The Ohio State University
Academic	Museum of Comparative Zoology, Harvard University	Museum of Comparative Zoology, Harvard University	The Museum of Comparative Zoology was founded in 1859 on the concept that collections are an integral and fundamental component of zoological research and teaching. This more than 150-year-old commitment remains a strong and proud tradition for the MCZ. The present-day MCZ contains over 21-million specimens in ten research collections which comprise one of the world's richest and most varied resources for studying the diversity of life.
Academic	Museums Victoria provider for OZCAM	Museums Victoria	Dataset from the Museums Victoria
Academic	Entomological Specimens of Museum of Nature and Human Activities, Hyogo Pref., Japan	National Institute of Genetics, ROIS	Dataset of Insect Specimens deposited in Museum of Nature and Human Activities, Hyogo Pref., Japan.

S2 Table. GBIF datasets which occurrence data contained entries labeled as "human observation" or "observation" according to the variable "basisOfRecord" from the GBIF Darwin Core Archive. Each dataset is sorted either as "academic" or "citizen science" in the *Category* column based on the nature of the data collection (i.e. data from literature or that was gathered following standardized protocols is considered "academic" and data collected without following any sampling protocol is considered "citizen science"). Note that some datasets were removed. Dataset descriptions were taken and/or adapted from their own metadata on GBIF. These datasets can be found in https://doi.org/10.15468/dl.mm74qg.

Category	Dataset	Publisher	Description
Academic	NMNH Extant Specimen Records (USNM, US)	National Museum of Natural History, Smithsonian Institution	Dataset of public records of accessioned specimens and observations curated by the National Museum of Natural History, Smithsonian Institution. These data are from the Departments of Botany, Entomology, Invertebrate Zoology and Vertebrate Zoology (Amphibians & Reptiles, Birds, Fishes, and Mammals) and include more than 270,000 primary type specimen records.
Academic	NMNH Material Samples (USNM)	National Museum of Natural History, Smithsonian Institution	Genetic Sample data for GGBN and other users.
Academic		National Museum of Nature and Science	Datasets of the National Museum of Nature and Science, Japan.
Academic		National Museums of Kenya	Datasets of the National Museums of Kenya
Academic	Natural History Museum (London) Collection Specimens	Natural History Museum	Natural History Museum (London) Collection Specimens
Academic	VIT-Coleopterotheca (The Natural History Museum of Álava)	Natural History Museum of Alava (Museo de Ciencias Naturales de Álava / Arabako Natura Zientzien Museoa)	The Coleopterotheca of the Natural History Museum of Álava began in 1974 and nowadays, it holds around 160.000 specimens, of which 133.700 are databased.
Academic	VIT - Lepidotheca (The Natural History Museum of Álava)	Natural History Museum of Alava (Museo de Ciencias Naturales de Álava / Arabako Natura Zientzien Museoa)	The Lepidotheca of the Natural Science Museum of Álava contains 80,000 specimens, 75,600 of them databased, belonging to 5,831 taxa.

S2 Table. GBIF datasets which occurrence data contained entries labeled as "human observation" or "observation" according to the variable "basisOfRecord" from the GBIF Darwin Core Archive. Each dataset is sorted either as "academic" or "citizen science" in the *Category* column based on the nature of the data collection (i.e. data from literature or that was gathered following standardized protocols is considered "academic" and data collected without following any sampling protocol is considered "citizen science"). Note that some datasets were removed. Dataset descriptions were taken and/or adapted from their own metadata on GBIF. These datasets can be found in https://doi.org/10.15468/dl.mm74qg.

Category	Dataset	Publisher	Description
Academic	LACM Entomology Collection	Natural History Museum of Los Angeles County	The Natural History Museum's entomology collection has more than 5.8 million specimens of insects and spiders.
Academic		Natural History Museum of Utah (UMNH)	Invertebrate datasets from the UMHN.
Academic	Natural History Museum Rotterdam - Specimens	Natural History Museum Rotterdam	Specimen database of the Natural History Museum Rotterdam. The total collection is estimated to hold 420.000 specimens, of which over 85% are digitized at the specimen level and contained in this dataset. Arthropods (56%; mostly European) and molluscs (30%; worldwide recent specimens, fossil specimens from North-West Europe) make up the bulk of the collection.
Academic	Texas Tech University - Invertebrate Zoology	Natural Science Research Laboratory, Museum of Texas Tech University (TTU)	The Invertebrate Zoology collection (TTU-Z) of the Museum of Texas Tech University is international in scope with an emphasis on arthropods from semi-arid and arid lands, especially from North America.
Academic		Naturalis Biodiversity Center	Naturalis Biodiversity Center is the result of cooperation between Amsterdam University (Amsterdam Zoological Museum), Leiden University and Wageningen University and Research Centre (National Herbarium Nederland) and the Dutch National Natural History Museum Naturalis in Leiden. Naturalis Biodiversity Center is an active research and education center and hosts one of the largest natural history collections in the world.

S2 Table. GBIF datasets which occurrence data contained entries labeled as "human observation" or "observation" according to the variable "basisOfRecord" from the GBIF Darwin Core Archive. Each dataset is sorted either as "academic" or "citizen science" in the *Category* column based on the nature of the data collection (i.e. data from literature or that was gathered following standardized protocols is considered "academic" and data collected without following any sampling protocol is considered "citizen science"). Note that some datasets were removed. Dataset descriptions were taken and/or adapted from their own metadata on GBIF. These datasets can be found in https://doi.org/10.15468/dl.mm74qg.

Category	Dataset	Publisher	Description
Academic	Swiss Psyllid (Hemiptera) Collections - Basel	Naturhistorisches Museum Basel - NMB	Database of jumping plant-lice of the families Calophyidae, Carsidaridae, Homotomidae, Phacopteronidae and Triozidae of the Naturhistorisches Museum Basel (NMB) and Muséum d'histoire naturelle Genève (MHNG) - Basel part.
Academic	Naturhistorisches Museum Mainz, Zoological Collection	Naturhistorisches Museum Mainz	The zoological collection of the Mainz Natural History Museum / State Collection of Natural History of Rhineland-Palatinate is the largest in Rhineland-Palatinate and holds approx. 750.000 preserved specimens.
Academic	Naturkundemuseum im Ottoneum Kassel, Entomological Collection	Naturkundemuseum im Ottoneum Kassel	Entomological dataset of the collection from the Naturkundemuseum im Ottoneum Kassel
Academic	Natuurmuseum Brabant, Tilburg - Invertebrates	Natuurmuseum Brabant	The invertebrate dataset contains all registered invertebrate specimens of the Natural History Museum Brabant (Tilburg, Netherlands).
Academic	New York State Museum Arthropod Collection	New York State Museum (NYSM)	The insect collection contains more than one million general specimens and approximately 1,000 type specimens (excluding the gall midge types), mainly from New York State. The last decade has seen rapid expansion both in number of specimens and area of coverage.
Academic		Norwegian University of Science and Technology	Compilation of Insecta datasets from the NTNU.
Academic		Plazi.org taxonomic treatments database	Dataset from Plazi.org

S2 Table. GBIF datasets which occurrence data contained entries labeled as "human observation" or "observation" according to the variable "basisOfRecord" from the GBIF Darwin Core Archive. Each dataset is sorted either as "academic" or "citizen science" in the *Category* column based on the nature of the data collection (i.e. data from literature or that was gathered following standardized protocols is considered "academic" and data collected without following any sampling protocol is considered "citizen science"). Note that some datasets were removed. Dataset descriptions were taken and/or adapted from their own metadata on GBIF. These datasets can be found in https://doi.org/10.15468/dl.mm74qg.

Category	Dataset	Publisher	Description
Academic	World flea collection of Slovenian Museum of Natural History (excluding Slovenia)	Prirodoslovni muzej Slovenije	Central Slovenian Scientific Collection of Siphonaptera includes 8,497 microscopic slides and some material preserved in ethanol.
Academic	Instituto de Investigación CIBIO, Universidad de Alicante: CEUA	Research Institute CIBIO (Centro Iberoamericano de la Biodiversidad), University of Alicante	The entomological collection of the University of Alicante (CEUA) is deposited and guarded in the Ibero-American Biodiversity Center (CIBIO), University Research Institute of the University of Alicante. The scientific collection houses more than 600,000 specimens and includes more than 3,500 different species of insects, which places it among one of the reference collections of insects in Spain.
Academic	Royal Belgian Institute of Natural Sciences Collection	Royal Belgian Institute of Natural Sciences	The Royal Belgian Institute of Natural Sciences houses a precious collection of zoological, anthropological, paleontological, mineralogical and geological materials and data. The renowned Iguanodons from Bernissart, ambassadors of the Belgian science institute in Brussels, represent a natural history collection currently estimated to hold over 37 million specimens.
Academic		Sam Noble Oklahoma Museum of Natural History	Datasets from the Sam Noble Oklahoma Museum of Natural History.
Academic	Colección Orthoptera y Dermaptera Antonio del Cerro (Museo de Zoología Ibérica Antonio Notario) UPM	School of Forestry Engineering. Technical University of Madrid	The Antonio del Cerro collection consists of about 7,000 specimens of Orthoptera and Dermaptera, among others, captured in the Sierra de Cazorla (Jaén) in the years 1975, 1976 and 1977. The collection is located in the collection of the Antonio Notario Museum of Iberian Zoology , belonging to the Department of Systems and Natural Resources of the ETSMFNM (UPM).
Academic		Senckenberg	Datasets from Senckenberg
Academic		South African National Biodiversity Institute	Dataset compilation from the South African National Biodiversity Institute

S2 Table. GBIF datasets which occurrence data contained entries labeled as "human observation" or "observation" according to the variable "basisOfRecord" from the GBIF Darwin Core Archive. Each dataset is sorted either as "academic" or "citizen science" in the *Category* column based on the nature of the data collection (i.e. data from literature or that was gathered following standardized protocols is considered "academic" and data collected without following any sampling protocol is considered "citizen science"). Note that some datasets were removed. Dataset descriptions were taken and/or adapted from their own metadata on GBIF. These datasets can be found in https://doi.org/10.15468/dl.mm74qg.

Category	Dataset	Publisher	Description
Academic		Swedish Museum of Natural History	Dataset compilation from the Swedish Museum of Natural History
Academic	Tasmanian Museum and Art Gallery provider for OZCAM	Tasmanian Museum and Art Gallery	Tasmanian Museum and Art Gallery provider for OZCAM
Academic	Texas A&M University Insect Collection	Texas A&M University Insect Collection	The Texas A&M University Insect Collection (TAMUIC) is a land-grant-university-based collection of insects and related arthropods whose primary holdings are focused on the regional faunas of the south-central and southwestern United States and the northern neotropics, especially Mexico.
Academic	International Barcode of Life project (iBOL)	The International Barcode of Life Consortium	Dataset from the International barcode of Life project.
Academic	Wasps from the UWIZM Land Arthropod collection	The University of the West Indies Zoology Museum (UWIZM)	This dataset contains the records of wasps belonging to UWIZM's land arthropod collection. There are 5248 wasp specimens spanning eight families. These specimens are all stored in the Land Arthropod Room of the UWIZM.
Academic	UWIZM International Lepidoptera Collection	The University of the West Indies Zoology Museum (UWIZM)	This dataset contains some of the records from the UWIZM Lepidoptera collection with specimens from across the world. Most are from UWIZM collections but some are also from CABI's (Centre for Agriculture and Bioscience International).
Academic		Universidad Central de Venezuela	Dataset compilation from the Universidad Central de Venezuela
Academic	Arthropods from Praia da Vitória (Terceira, Azores, Portugal)	Universidade dos Açores	Dataset with a total of 15,810 specimens belonging to 216 arthropod species and subspecies were collected.
Academic	Inventory of Arthropods of Azorean Urban Gardens	Universidade dos Açores	The data we present are part of the long-term project SLAM (Long Term Ecological Study of the Impacts of Climate Change in the natural forest of Azores) aiming to

S2 Table. GBIF datasets which occurrence data contained entries labeled as "human observation" or "observation" according to the variable "basisOfRecord" from the GBIF Darwin Core Archive. Each dataset is sorted either as "academic" or "citizen science" in the *Category* column based on the nature of the data collection (i.e. data from literature or that was gathered following standardized protocols is considered "academic" and data collected without following any sampling protocol is considered "citizen science"). Note that some datasets were removed. Dataset descriptions were taken and/or adapted from their own metadata on GBIF. These datasets can be found in https://doi.org/10.15468/dl.mm74qg.

Category	Dataset	Publisher	Description
			assess the impact of biodiversity erosion drivers on Azorean native biota, using long-term ecological data. Additionally to SLAM traps, nocturnal Active Aerial Searching and nocturnal Foliage Beating methods were used to sample, between 2017 and 2018, the arthropod biodiversity on two historical urban gardens of Azores, the "Jardim Botânico" of Faial Island, and "Jardim Duque da Terceira" of Terceira Island.
Academic	Inventory of the Azorean rove-beetles (Insecta, Coleoptera, Staphylinidae)	Universidade dos Açores	The data presented consists in a long-term inventory of Staphylinidae (Insecta, Coleoptera) collected from several standardized sampling campaigns and non-standardized observations, performed between 1990 and 2015 in several habitat types of eight islands of the Azores archipelago.
Academic	Biodiversity data of lady beetles (Coleoptera: Coccinellidae) of the Azores archipelago (Portugal)	Universidade dos Açores	A recent review study from 2021 presents a comprehensive checklist of ladybirds of Portugal, including the Azores and Madeira Archipelagos.
Academic	Monitoring ground arthropods in maize and pasture fields of São Miguel and São Jorge Islands: IPM-Popillia Project	Universidade dos Açores	The dataset presented here is the delivery of the European project "Integrated Pest Management of the Invasive Japanese Beetle, Popillia japonica (IPM-Popillia)". This project aims to address the challenge of a new risk to plant health in Europe, the invasion of the Japanese beetle, Popillia japonica, and to provide an environmentally friendly IPM-Toolbox to control the pest in infested areas, protecting the agricultural systems and control this pest populations current in expansion across Europe.
Academic	Diversity of Lepidoptera recorded in a forest nursery of Nordeste county on São Miguel Island (Azores)	Universidade dos Açores	The diversity of moth species (Insecta, Lepidoptera) recorded in the forest nursery of Nordeste county on São Miguel island (Azores) is given. Adults were sampled between March and December 2019 using three methods: (i) light trap to caught Noctuidae species, (ii) open-sided delta trap baited with a synthetic female sex pheromone lure to attract Epiphyas postvittana males and (iii) entomological net

S2 Table. GBIF datasets which occurrence data contained entries labeled as "human observation" or "observation" according to the variable "basisOfRecord" from the GBIF Darwin Core Archive. Each dataset is sorted either as "academic" or "citizen science" in the *Category* column based on the nature of the data collection (i.e. data from literature or that was gathered following standardized protocols is considered "academic" and data collected without following any sampling protocol is considered "citizen science"). Note that some datasets were removed. Dataset descriptions were taken and/or adapted from their own metadata on GBIF. These datasets can be found in https://doi.org/10.15468/dl.mm74qg.

Category	Dataset	Publisher	Description
			to identify microlepidopteran moths. A total of 10160 adults belonging to 33 lepidopteran species were recorded and listed by families.
Academic	Coleção Entomológica Padre Jesus Santiago Moure (DZUP) - Coleoptera	Universidade Federal do Paraná	Dataset of the collection of Padre Jesus Santiago Moure, with approximately seven million insects representing mainly the orders Coleoptera, Diptera, Hemiptera, Hymenoptera and Lepidoptera.
Academic	Coleção Entomológica Padre Jesus Santiago Moure (DZUP) - Diptera	Universidade Federal do Paraná	Dataset of the collection of Padre Jesus Santiago Moure, with approximately seven million insects representing mainly the orders Coleoptera, Diptera, Hemiptera, Hymenoptera and Lepidoptera.
Academic	Ouellet-Robert Entomological Collection (QMOR) - Specimens	Université de Montréal Biodiversity Centre	The Ouellet-Robert entomological collection (QMOR) is one of the larger Canadian university entomological collections and the second largest in Quebec. It comprises 1.5 million specimens, covering more than 20,000 species.
Academic		University of Alaska Museum of the North	Datasets from the University of Alaska Museum of the North
Academic	University of Alberta E. H. Strickland Entomological Museum (UASM)	University of Alberta Museums	The E. H. Strickland Entomological Museum houses approximately one million specimens. The research collection includes principally Nearctic insects, representing most orders and the major families thereof.
Academic		University of Bergen	Dataset compilation from the University of Bergen
Academic	Snow Entomological Museum Collection	University of Kansas Biodiversity Institute	SEMC (Snow Entomological Museum Collection) comprises nearly 5 million pinned insect specimens. Our strengths are Apoidea, Neotropical Coleoptera (especially Staphylinidae, Hydrophiloidea, and Chrysomoloidea), Mecoptera, and fossil insects.

S2 Table. GBIF datasets which occurrence data contained entries labeled as "human observation" or "observation" according to the variable "basisOfRecord" from the GBIF Darwin Core Archive. Each dataset is sorted either as "academic" or "citizen science" in the *Category* column based on the nature of the data collection (i.e. data from literature or that was gathered following standardized protocols is considered "academic" and data collected without following any sampling protocol is considered "citizen science"). Note that some datasets were removed. Dataset descriptions were taken and/or adapted from their own metadata on GBIF. These datasets can be found in https://doi.org/10.15468/dl.mm74qg.

Category	Dataset	Publisher	Description
Academic	Hymenoptera Institute Collection	University of Kentucky	The Hymenoptera Institute Collection is a collection of largely parasitic Hymenoptera housed in Michael Sharkey's Lab in the Entomology Department (Agricultural Science Center North) at the University of Kentucky. The museum houses over 350 drawers of Hymenoptera focusing on Braconidae, with the primary focus on the braconid subfamily Agathidinae.
Academic	University of Michigan Museum of Zoology, Division of Insects	University of Michigan Museum of Zoology	This collection contains catalog records from the University of Michigan Museum of Zoology's Insect Division's specimen collection. Many specimen records include a specimen and label data image. The database currently contains about 300,000 specimen records out of the estimated 3 million estimated specimens in the collection.
Academic	Freshwater samples in MZNA-INV-FRW: Macroinvertebrate samples from the water quality monitoring network along the Ebro Basin	University of Navarra – Department of Environmental Biology	This dataset gathers information about the macroinvertebrate samples collected on rivers and streams of the Ebro River Basin (NE Iberian Peninsula), the second largest catchment in the Iberian Peninsula.
Academic		University of Nebraska State Museum	Dataset compilation from the University of Nebraska State Museum.
Academic		University of Oslo	Insect compilation dataset from the University of Oslo.
Academic		University of Oulu	Dataset compilation from the University of Oulu
Academic		University of Tartu, Natural History Museum and Botanical Garden	Dataset compilation from theUniversity of Tartu, Natural History Museum and Botanical Garden

S2 Table. GBIF datasets which occurrence data contained entries labeled as “human observation” or “observation” according to the variable “basisOfRecord” from the GBIF Darwin Core Archive. Each dataset is sorted either as “academic” or “citizen science” in the *Category* column based on the nature of the data collection (i.e. data from literature or that was gathered following standardized protocols is considered “academic” and data collected without following any sampling protocol is considered “citizen science”). Note that some datasets were removed. Dataset descriptions were taken and/or adapted from their own metadata on GBIF. These datasets can be found in https://doi.org/10.15468/dl.mm74qg.

**Category**	**Dataset**	**Publisher**	**Description**
Academic		University of Tennessee - Chattanooga (UTC)	Insect dataset compilation from the University of Tennessee - Chattanooga (UTC)
Academic		University of Texas at Austin, Biodiversity Collections	Dataset compilation from the University of Texas at Austin, Biodiversity Collections
Academic		University of Texas at El Paso Biodiversity Collections	Dataset compilation from the El Paso biodiversity Collections.
Academic		University of Wisconsin - Madison	The Wisconsin collection is the result of nearly 170 years of collecting by amateur and professional entomologists. Early collecting efforts focused on increasing holdings of Coleoptera, Diptera, and Lepidoptera, while the current research strengths of the WIRC include Coleoptera, Hymenoptera, Diptera, Lepidoptera, and Hemiptera.
Academic		USDA-ARS Pollinating Insect-Biology, Management, Systematics Research	Dataset compilation
Academic		Virginia Tech Insect Collection	The Virginia Tech Insect Collection, founded in 1888, is the oldest and largest entomological collection in the Commonwealth. Its nearly half-million specimens represent the rich insect diversity of the eastern United States with emphasis on the Appalachian region. This collection is an exceptional repository of pollinators, endangered insects, and many native species, once common but now disappearing from habitat loss.

S2 Table. GBIF datasets which occurrence data contained entries labeled as "human observation" or "observation" according to the variable "basisOfRecord" from the GBIF Darwin Core Archive. Each dataset is sorted either as "academic" or "citizen science" in the *Category* column based on the nature of the data collection (i.e. data from literature or that was gathered following standardized protocols is considered "academic" and data collected without following any sampling protocol is considered "citizen science"). Note that some datasets were removed. Dataset descriptions were taken and/or adapted from their own metadata on GBIF. These datasets can be found in https://doi.org/10.15468/dl.mm74qg.

Category	Dataset	Publisher	Description
Academic		Wrocław University, Museum of Natural History	Dataset compilation from the Museum of Natural History
Academic		Yale University Peabody Museum	Data compilation from the Yale University Peabody Museum
Academic		ZooKeys	Data compilation from the ZooKeys journal.
Academic		Zoological collections of the University of Leon (CZULE), University of Leon	Datasets of the Zoological collections of the University of Leon (CZULE), University of Leon
Academic		Staatliche Naturwissenschaftliche Sammlungen Bayerns	Dataset compilation from the Staatliche Naturwissenschaftliche Sammlungen Bayerns

References from the GBIF datasets:

A.J. Cook Arthropod Research Collection (2023). The Albert J. Cook Arthropod Research Collection. Occurrence dataset https://doi.org/10.15468/mtagq6 accessed via GBIF.org on 2023-06-03.

Abbott J (2023). Odonata Central. Version 1.576. Odonata Central. Occurrence dataset https://doi.org/10.15468/vbpgex accessed via GBIF.org on 2023-06-03.

Adam Mickiewicz University in Poznań. Natural History Collections of the Faculty of Biology AMU. Occurrence dataset https://doi.org/10.15468/54hgbz accessed via GBIF.org on 2023-06-03.

Aguado-Aranda P, Ricarte A, Nedeljković Z, Marcos-García M Á, felipe (2022). An overlooked case for a century: taxonomy and systematics of a new Iberian species of Eumerus Meigen, 1822 (Diptera, Syrphidae). Plazi.org taxonomic treatments database. Checklist dataset https://doi.org/10.15468/c5sb9n accessed via GBIF.org on 2023-06-03.

Alaman A, @abellerol, Pérez T, Anecdata.org Contributors, Disney J, Bailey C, Taylor A, Garretson A (2022). Lechuzas l'Horta Nord - Powered by Anecdata.org. The Community Environmental Health Laboratory at MDI Biological Laboratory. Occurrence dataset https://doi.org/10.15468/x9x2de accessed via GBIF.org on 2023-06-03.

Anton M (2023). Data collected on citizen science web portal www.ornitho.cat. Version 2.27. ICO-Institut Català d'Ornitologia (Catalan Ornithological Institute). Occurrence dataset https://doi.org/10.15470/dijq3a accessed via GBIF.org on 2023-06-03.

Araújo G, Neves T (2021). Habitats Directive Portuguese Report: Species 2007-2012. ICNF - Instituto da Conservação da Natureza e das Florestas. Occurrence dataset https://doi.org/10.15468/46bfcv accessed via GBIF.org on 2023-06-03.

Araújo G, Neves T (2021). Habitats Directive Portuguese Report: Species 2013-2018. ICNF - Instituto da Conservação da Natureza e das Florestas. Occurrence dataset https://doi.org/10.15468/asr26t accessed via GBIF.org on 2023-06-03.

Arizona State University Biocollections (2023). Arizona State University Hasbrouck Insect Collection. Occurrence dataset https://doi.org/10.15468/vv6k58 accessed via GBIF.org on 2023-06-03.

Assing V, carolina (2012). On the taxonomy and zoogeography of some Oxypoda species of the West Palaearctic region (Coleoptera: Staphylinidae: Aleocharinae). Plazi.org taxonomic treatments database. Checklist dataset https://doi.org/10.15468/q2eeve accessed via GBIF.org on 2023-06-03.

Assing V, carolina (2018). A revision of Medon. XI. Five new species, additional records, and the first confirmed records from the Oriental region (Coleoptera: Staphylinidae: Paederinae). Plazi.org taxonomic treatments database. Checklist dataset https://doi.org/10.15468/u2tbnz accessed via GBIF.org on 2023-06-03.

Assing V, carolina (2021). On the Cephennium fauna of the Iberian Peninsula and the Atlantic Islands (Coleoptera, Staphylinidae, Scydmaeninae). Plazi.org taxonomic treatments database. Checklist dataset https://doi.org/10.15468/zea9kt accessed via GBIF.org on 2023-06-03.

Assing V, felipe (2015). A new species of Phloeocharis MANNERHEIM from Spain, with a note on P. brachyptera SHARP (Coleoptera: Staphylinidae: Phloeocharinae). Plazi.org taxonomic treatments database. Checklist dataset https://doi.org/10.5281/zenodo.4526324 accessed via GBIF.org on 2023-06-03.

Assing V, felipe (2015). A revision of Sunius XV. Six new species from Kyrgyzstan and the Himalaya, and additional records (Coleoptera: Staphylinidae: Paederinae). Plazi.org taxonomic treatments database. Checklist dataset https://doi.org/10.15468/vyqgmp accessed via GBIF.org on 2023-06-03.

Assing V, felipe (2016). Revision of the Anaulacaspis species of the Palaearctic region (Coleoptera: Staphylinidae: Aleocharinae). Plazi.org taxonomic treatments database. Checklist dataset https://doi.org/10.15468/q59utb accessed via GBIF.org on 2023-06-03.

Assing V, marcus (2013). On the Palaearctic and Oriental species of Scymbalium and Micrillus (Coleoptera: Staphylinidae: Paederinae). Plazi.org taxonomic treatments database. Checklist dataset https://doi.org/10.15468/t6gmv8 accessed via GBIF.org on 2023-06-03.

Assing V, marcus (2016). On some Acanthoglossa and Hypomedon species II. Two new species, a new synonymy, and additional records (Coleoptera: Staphylinidae: Paederinae). Plazi.org taxonomic treatments database. Checklist dataset https://doi.org/10.15468/ejf7d4 accessed via GBIF.org on 2023-06-03.

Assing V, marcus (2017). A revision of Leptobium CASEY. VIII. A new species from Iran, a new synonymy, and additional records (Coleoptera: Staphylinidae: Paederinae). Plazi.org taxonomic treatments database. Checklist dataset https://doi.org/10.15468/8cgvsd accessed via GBIF.org on 2023-06-03.

Assing V, Vogel J, valdenar (2019). The mother of synonyms: on the Meotica species of the Palaearctic Region (Coleoptera, Staphylinidae, Aleocharinae, Oxypodini). Plazi.org taxonomic treatments database. Checklist dataset https://doi.org/10.5281/zenodo.3738506 accessed via GBIF.org on 2023-06-03.

Audibert C, Flye Sainte Marie M (2019). Dermaptera. Version 1.11. Musée des Confluences. Occurrence dataset https://doi.org/10.15468/ejmxzy accessed via GBIF.org on 2023-06-03.

Audibert C, Flye Sainte Marie M (2021). Phengaris. Musée des Confluences. Occurrence dataset https://doi.org/10.15468/tgkatp accessed via GBIF.org on 2023-06-03.

Australian Museum (2021). Australian Museum provider for OZCAM. Occurrence dataset https://doi.org/10.15468/e7susi accessed via GBIF.org on 2023-06-03.

Bakker F, Creuwels J (2023). Naturalis Biodiversity Center (NL) - Hymenoptera. Naturalis Biodiversity Center. Occurrence dataset https://doi.org/10.15468/jgywgc accessed via GBIF.org on 2023-06-03.

Barea-Azcón J M (2021). Dataset of butterfly monitoring in Sierra Nevada (Spain). Version 1.2. Sierra Nevada Global Change Observatory. Andalusian Environmental Center, University of Granada, Regional Government of Andalusia. Sampling event dataset https://doi.org/10.15470/tc3gdq accessed via GBIF.org on 2023-06-03.

Barták M, Kubík Š, plazi (2016). New species and new synonyms in European Platypalpus (Diptera: Hybotidae). Plazi.org taxonomic treatments database. Checklist dataset https://doi.org/10.11646/zootaxa.4175.2.3 accessed via GBIF.org on 2023-06-03.

Bartsch D, Pühringer F, Milla L, Lingenhöle A, Kallies A, plazi (2021). A molecular phylogeny and revision of the genus Pyropteron Newman, 1832 (Lepidoptera, Sesiidae) reveals unexpected diversity and frequent hostplant switch as a driver of speciation. Plazi.org taxonomic treatments database. Checklist dataset https://doi.org/10.11646/zootaxa.4972.1.1 accessed via GBIF.org on 2023-06-03.

Beja P, Figueira R, Corley M, Grosso-Silva J M, Ferreira S, Sousa P (2018). EDP Foz Tua: Arthropoda – Environmental Impact Assessment [2006-2008]. Version 1.6. EDP - Energias de Portugal. Occurrence dataset https://doi.org/10.15468/jtdrhm accessed via GBIF.org on 2023-06-03.

Bentley A, Thomas J (2023). Snow Entomological Museum Collection. Version 26.71. University of Kansas Biodiversity Institute. Occurrence dataset https://doi.org/10.15468/fhntpy accessed via GBIF.org on 2023-06-03.

Bidzilya O V, Budashkin Y I, Gaedike R, plazi (2016). A revision of the Eudarcia glaseri - species group (Lepidoptera, Meessiidae) with description of two new species from Greece and Crimea. Plazi.org taxonomic treatments database. Checklist dataset https://doi.org/10.11646/zootaxa.4179.3.9 accessed via GBIF.org on 2023-06-03.

Biological Collections Division (2022). Canadian National Collection of Insects, Arachnids, and Nematodes (CNC). Version 1.1. Agriculture and Agri-Food Canada. Occurrence dataset https://doi.org/10.5886/l7s5yj accessed via GBIF.org on 2023-06-03.

Biologiezentrum Linz Oberoesterreich. Biologiezentrum Linz. Occurrence dataset https://doi.org/10.15468/ynjblx accessed via GBIF.org on 2023-06-03.

Blindheim T (2023). Biofokus. Version 1.2376. Biofokus. Occurrence dataset https://doi.org/10.15468/jxbhqx accessed via GBIF.org on 2023-06-03.

Boieiro M, Antunes S, Figueiredo H, Soares A, Lopes A, Monteiro E, Garcia Pereira P, Rego C, Conde J, Borges P A V, Serrano A R M (2023). Standardised sampling of lepidopterans (Lepidoptera) in Serra da Estrela (Portugal) - 2013 and 2014. Version 1.6. Universidade dos Açores. Sampling event dataset https://doi.org/10.15468/uvnv9j accessed via GBIF.org on 2023-06-03.

Boieiro M, Antunes S, Figueiredo H, Soares A, Lopes A, Monteiro E, Garcia Pereira P, Rego C, Conde J, Borges P A V, Serrano A R M (2023). Standardised sampling of odonates (Odonata) in Serra da Estrela (Portugal) - 2013 and 2014. Version 1.6. Universidade dos Açores. Sampling event dataset https://doi.org/10.15468/6zz3yp accessed via GBIF.org on 2023-06-03.

Borges P A V, Gabriel R, Arroz A M, Costa A, Cunha R, Silva L, Pereira E, Martins A F, Reis F, Cardoso P (2023). Azorean Biodiversity Portal. Version 1.2. Universidade dos Açores. Occurrence dataset https://doi.org/10.15468/j0iceo accessed via GBIF.org on 2023-06-03.

Borges P A V, Gabriel R, M. Pimentel C M, Brito M R, Serrano A R M, Crespo L C F, Assing V, Stüben P, Fattorini S, Pereira E P, Nogueira E, Soares A O (2023). Arthropods from Praia da Vitória (Terceira, Azores, Portugal). Version 1.5. Universidade dos Açores. Sampling event dataset https://doi.org/10.15468/mjf3rt accessed via GBIF.org on 2023-06-03.

Borges P A V, Lamelas-López L (2023). Inventory of Arthropods of Azorean Urban Gardens. Version 1.6. Universidade dos Açores. Sampling event dataset https://doi.org/10.15468/bkynkp accessed via GBIF.org on 2023-06-03.

Borges P A V, Lamelas-López L (2023). Monthly monitoring of Azorean forest arthropods testing for edge effects (Terceira Island, Azores, Portugal). Version 1.5. Universidade dos Açores. Sampling event dataset https://doi.org/10.15468/k84m4e accessed via GBIF.org on 2023-06-03.

Borges P A V, Lamelas-López L, Assing V, Schülke M (2022). Inventory of the Azorean rove-beetles (Insecta, Coleoptera, Staphylinidae). Version 1.6. Universidade dos Açores. Sampling event dataset https://doi.org/10.15468/fh6qar accessed via GBIF.org on 2023-06-03.

Borges P A V, Lamelas-López L, Nunes R, Monjardino P, Lopes D H, Soares A O, Ferrante M (2022). Monitoring Arthropods in Azorean Agroecosystems: the project AGRO-ECOSERVICES. Version 1.12. Universidade dos Açores. Sampling event dataset https://doi.org/10.15468/mvtmyx accessed via GBIF.org on 2023-06-03.

Borges P A V, Lamelas-López L, Ros-Prieto A (2023). A survey of exotic arthropods in disturbed Azorean forest habitats using SLAM traps. Version 1.8. Universidade dos Açores. Sampling event dataset https://doi.org/10.15468/ucfehv accessed via GBIF.org on 2023-06-03.

Borges P A V, Lhoumeau S (2023). Long-term monitoring of Azorean forest arthropods. Version 1.5. Universidade dos Açores. Sampling event dataset https://doi.org/10.15468/3r5xw6 accessed via GBIF.org on 2023-06-03.

Botanic Garden and Botanical Museum Berlin. Harpactus Shuckard. Occurrence dataset https://doi.org/10.15468/yxylrc accessed via GBIF.org on 2023-06-03.

Botey B, Cernocky T, Tardà A, Ju A, Pizarro M (2018). IASTracker. Invasive Alien Species database. Version 2.2. IC5Team. Occurrence dataset https://doi.org/10.15470/tlnehc accessed via GBIF.org on 2023-06-03.

Braun J, King P (2022). Recent Invertebrates Specimens. Version 101.172. Sam Noble Oklahoma Museum of Natural History. Occurrence dataset https://doi.org/10.15468/glxcep accessed via GBIF.org on 2023-06-03.

Brigham Young University, Arthropod Collection (2023). Brigham Young University Arthropod Museum. Occurrence dataset https://doi.org/10.15468/gqf6no accessed via GBIF.org on 2023-06-03.

Caballero López B, Agulló Villaronga J (2023). Museu de Ciències Naturals de Barcelona: MCNB-Art. Museu de Ciències Naturals de Barcelona. Occurrence dataset https://doi.org/10.15468/pewzzr accessed via GBIF.org on 2023-06-03.

Caritg R, Martínez de la Riva S (2016). Artròpodes d'Andorra. Version 1.3. Centre d'estudis de la neu i de la muntanya d'Andorra (CENMA), Institut d'Estudis Andorrans. Occurrence dataset https://doi.org/10.15468/d9uwhe accessed via GBIF.org on 2023-06-03.

Carrera D (2018). DIBA-Parc Natural del Montseny. Version 1.2. Diputació de Barcelona. Occurrence dataset https://doi.org/10.15470/ug15ii accessed via GBIF.org on 2023-06-03.

Caspers M, Creuwels J (2023). Naturalis Biodiversity Center (NL) - Hemiptera. Naturalis Biodiversity Center. Occurrence dataset https://doi.org/10.15468/aw4q6q accessed via GBIF.org on 2023-06-03.

Caspers M, Creuwels J (2023). Naturalis Biodiversity Center (NL) - Odonata. Naturalis Biodiversity Center. Occurrence dataset https://doi.org/10.15468/r2rwih accessed via GBIF.org on 2023-06-03.

Ceccolini F (2022). New records for crown wasps in Europe (Hymenoptera, Stephanidae). Version 1.3. Museu de Ciències Naturals de Barcelona. Occurrence dataset https://doi.org/10.15470/baost7 accessed via GBIF.org on 2023-06-03.

Christoph Germann, Carlo Braunert, plazi (2016). Orthochaetes estrelanus sp. n., a new species from northern Portugal and new records of other Styphlini (Coleoptera, Curculionidae).. Plazi.org taxonomic treatments database. Checklist dataset https://doi.org/10.5281/zenodo.269456 accessed via GBIF.org on 2023-06-03.

Ciliberti P, Creuwels J (2023). Naturalis Biodiversity Center (NL) - Diptera. Naturalis Biodiversity Center. Occurrence dataset https://doi.org/10.15468/cl6p3l accessed via GBIF.org on 2023-06-03.

Citizen Science Observation Dataset B, Tiago P (2020). Biodiversity4all Research-Grade Observations. BioDiversity4All. Occurrence dataset https://doi.org/10.15468/njmmp7 accessed via GBIF.org on 2023-06-03.

Clemson University Arthropod Collection (2023). Clemson University Arthropod Collection. Occurrence dataset https://doi.org/10.15468/fvigdc accessed via GBIF.org on 2023-06-03.

Colorado State University, C.P. Gillette Museum of Arthropod Diversity (2023). C.P. Gillette Museum of Arthropod Diversity. Occurrence dataset https://doi.org/10.15468/k0aett accessed via GBIF.org on 2023-06-03.

Commonwealth Scientific and Industrial Research Organisation (2021). Australian National Insect Collection. Occurrence dataset https://doi.org/10.15468/s1hm8r accessed via GBIF.org on 2023-06-03.

Conejero Ortega N, Jiménez Peydró R, Garzón García J (2020). Laboratorio de Entomología y Control de Plagas del Instituto Cavanilles de Biodiversidad y Biología Evolutiva de la Universidad de Valencia: ENV. Cavanilles Institute of Biodiversity and Evolutionary Biology, University of Valencia. Occurrence dataset https://doi.org/10.15468/inhesf accessed via GBIF.org on 2023-06-03.

Corley M, Afonso B (2021). Portuguese Lepidoptera records compiled by Martin Corley's database. Version 1.5. CIBIO (Research Center in Biodiversity and Genetic Resources) Portugal. Occurrence dataset https://doi.org/10.15468/ca4xt8 accessed via GBIF.org on 2023-06-03.

Corley M, Ferreira S, Buchner P, Sousa P, Beja P (2021). The InBIO Barcoding Initiative Database: Depressaria infernella Corley & Buchner, a new Iberian species of the Depressaria douglasella group. Version 1.2. CIBIO (Research Center in Biodiversity and Genetic Resources) Portugal. Occurrence dataset https://doi.org/10.15468/gcg7ny accessed via GBIF.org on 2023-06-03.

Corley M, Ferreira S, Lvovsky A L, Rosete J, Sousa P, Beja P (2020). The InBIO Barcoding Initiative Database: Borkhausenia crimnodes Meyrick, 1912 (Lepidoptera, Oecophoridae), a southern hemisphere species resident in Portugal. Version 1.1. CIBIO (Research Center in Biodiversity and Genetic Resources) Portugal. Occurrence dataset https://doi.org/10.15468/8yge73 accessed via GBIF.org on 2023-06-03.

Corley M, Ferreira S, Mata V A, Sousa P, Beja P (2021). The InBIO Barcoding Initiative Database: Ypsolopha rhinolophi sp. nov. (Lepidoptera: Ypsolophidae), a new species from Portugal and France unveiled by bats. Version 1.2. CIBIO (Research Center in Biodiversity and Genetic Resources) Portugal. Occurrence dataset https://doi.org/10.15468/zbu2w8 accessed via GBIF.org on 2023-06-03.

Corley M, Ferreira S, Rosete J, Sousa P, Beja P (2021). The InBIO Barcoding Initiative Database: Mondeguina, a new genus for Apatetris mediterranella Nel & Varenne, 2012, with description of a new species from Portugal (Lepidoptera, Gelechiidae). Version 1.2. CIBIO (Research Center in Biodiversity and Genetic Resources) Portugal. Occurrence dataset https://doi.org/10.15468/au3pbu accessed via GBIF.org on 2023-06-03.

Corley M, Ferreira S, Sousa P, Beja P (2021). The InBIO Barcoding Initiative Database: A taxonomic revision of the Western Palaearctic genus Cacochroa Heinemann, 1870 (Lepidoptera, Depressariidae). Version 1.2. CIBIO (Research Center in Biodiversity and Genetic Resources) Portugal. Occurrence dataset https://doi.org/10.15468/g5hudd accessed via GBIF.org on 2023-06-03.

Corley M, Ferreira S, Sousa P, Beja P (2021). The InBIO Barcoding Initiative Database: DNA Barcoding reveals sexual dimorphism in Isotrias penedana Trematerra, 2013 (Lepidoptera: Tortricidae, Chlidanotinae). Version 1.2. CIBIO (Research Center in Biodiversity and Genetic Resources) Portugal. Occurrence dataset https://doi.org/10.15468/9z8bpm accessed via GBIF.org on 2023-06-03.

Corley M, Ferreira S, Sousa P, Beja P (2021). The InBIO Barcoding Initiative Database: Taxonomic notes on Portuguese Microlepidoptera II. Cochylimorpha punctiferana (Ragonot, 1881). Version 1.2. CIBIO (Research Center in Biodiversity and Genetic Resources) Portugal. Occurrence dataset https://doi.org/10.15468/4u34gh accessed via GBIF.org on 2023-06-03.

Corley M, Ferreira S, Sousa P, Beja P (2022). The InBIO Barcoding Initiative Database: Ypsolopha milfontensis, a new species from the Portuguese coast (Lepidoptera: Ypsolophidae). Version 1.3. CIBIO (Research Center in Biodiversity and Genetic Resources) Portugal. Occurrence dataset https://doi.org/10.15468/92cdan accessed via GBIF.org on 2023-06-03.

Corral Arroyo J C, Sáenz J, López Del Valle R, Alonso Ramírez J, Infante Sánchez M (2023). VIT-Paleonthotheca (The Natural History Museum of Alava). Version 1.4. Natural History Museum of Alava (Museo de Ciencias Naturales de Álava / Arabako Natura Zientzien Museoa). Occurrence dataset https://doi.org/10.15470/scoumt accessed via GBIF.org on 2023-06-03.

Costa H, Salgueiro N, Marques A T, Coelho H, Monteiro B, Brás L, Gonçalves R, Oliveira J, Silva M, Sousa D, Caetano M, Romão F, Santos J, Peixoto M, Múrias T (2020). EDP Foz-Tua: Dragonflies and Damselflies (Odonata) - Ecological Monitoring Program (2011-2017). Version 1.1. EDP - Energias de Portugal. Sampling event dataset https://doi.org/10.15468/ywwyjd accessed via GBIF.org on 2023-06-03.

Costa H, Salgueiro N, Marques A T, Coelho H, Silva M, Caetano M, Romão F, Peixoto M, Múrias T (2021). EDP Foz-Tua: Diurnal Butterflies (Lepidoptera) - Ecological Monitoring Program (2014-2017). Version 1.3. EDP - Energias de Portugal. Sampling event dataset https://doi.org/10.15468/tcxun4 accessed via GBIF.org on 2023-06-03.

Cuesta-Segura A D, Jurado-Angulo P, Jiménez-Ruiz Y, García-París M, felipe (2023). Taxonomy of the Iberian species of Pseudochelidura (Dermaptera: Forficulidae). Plazi.org taxonomic treatments database. Checklist dataset https://doi.org/10.15468/5hsgx9 accessed via GBIF.org on 2023-06-03.

Cumming J M, plazi (2017). Revision of the Nearctic Parathalassius Mik (Diptera: Dolichopodidae: Parathalassiinae), with a review of the world fauna. Plazi.org taxonomic treatments database. Checklist dataset https://doi.org/10.11646/zootaxa.4314.1.1 accessed via GBIF.org on 2023-06-03.

Dalrymple A, Sankar S, Deacon A (2023). Wasps from the UWIZM Land Arthropod collection. The University of the West Indies Zoology Museum (UWIZM). Occurrence dataset https://doi.org/10.15468/r8egbb accessed via GBIF.org on 2023-06-03.

Davranoglou L, Baňař P, Suárez D, Martín S, Naranjo M, plazi (2022). A new cavernicolous assassin bug from the Canary Islands (Hemiptera: Reduviidae: Emesinae: Collartidini). Plazi.org taxonomic treatments database. Checklist dataset https://doi.org/10.15468/qw7exh accessed via GBIF.org on 2023-06-03.

de Meyer M, Heughebaert A (2014). Royal Museum of Central Africa - True Fruit Flies (Diptera, Tephritidae) of the Afrotropical Region (ENBI wp13). Belgian Biodiversity Platform. Occurrence dataset https://doi.org/10.15468/rtycar accessed via GBIF.org on 2023-06-03.

de Olano I, Infante Sánchez M (2023). VIT - Lepidotheca (The Natural History Museum of Álava). Version 1.2. Natural History Museum of Alava (Museo de Ciencias Naturales de Álava / Arabako Natura Zientzien Museoa). Occurrence dataset https://doi.org/10.15470/zfqjri accessed via GBIF.org on 2023-06-03.

de Vries H, Lemmens M (2023). Observation.org, Nature data from around the World. Observation.org. Occurrence dataset https://doi.org/10.15468/5nilie accessed via GBIF.org on 2023-06-03.

Delvare G, Escolà A R, Stojanova A M, Benoit L, Lecomte J, Askew R R, plazi (2019). Exploring insect biodiversity: the parasitic Hymenoptera, chiefly Chalcidoidea, associated with seeds of asphodels (Xanthorrhoeaceae), with the description of nine new species belonging to

Eurytomidae and Torymidae. Plazi.org taxonomic treatments database. Checklist dataset https://doi.org/10.11646/zootaxa.4597.1.1 accessed via GBIF.org on 2023-06-03.

Denver Museum of Nature & Science (2023). Denver Museum of Nature & Science - Entomology. Occurrence dataset https://doi.org/10.15468/3vhkol accessed via GBIF.org on 2023-06-03.

Department of Forest Sciences. European Moth Nights. Occurrence dataset https://doi.org/10.15468/rs5wtk accessed via GBIF.org on 2023-06-03.

Derafshan H A, Olmi M, Moghaddam M G, Nader E, Rakhshani E, felipe (2021). New records of Dryinidae Haliday, 1833 (Hymenoptera, Chrysidoidea) from Iran. Plazi.org taxonomic treatments database. Checklist dataset https://doi.org/10.5252/zoosystema2021v43a23 accessed via GBIF.org on 2023-06-03.

Díaz A, Martínez-Ortí A (2022). Las colecciones del Museu Valencià d'Història Natural. Version 2.5. Museu de Ciències Naturals de Barcelona. Occurrence dataset https://doi.org/10.15470/8oedep accessed via GBIF.org on 2023-06-03.

Dollfuss H, valdenar (2019). The Sphecid Wasps of the genus Cerceris L, 1802 of the " Biologiezentrum Linz " - Collection in Linz, Austria, from the Palearctic Region (part II). (Hymenoptera, Apoidea, Crabronidae). Plazi.org taxonomic treatments database. Checklist dataset https://doi.org/10.5281/zenodo.3738648 accessed via GBIF.org on 2023-06-03.

Edwin Lattke Bravo J, José Barros de Carvalho C (2023). Coleção Entomológica Padre Jesus Santiago Moure (DZUP) - Diptera. Universidade Federal do Paraná. Occurrence dataset https://doi.org/10.15468/phzk44 accessed via GBIF.org on 2023-06-03.

Edwin Lattke Bravo J, Massutti Almeida L (2023). Coleção Entomológica Padre Jesus Santiago Moure (DZUP) - Coleoptera. Universidade Federal do Paraná. Occurrence dataset https://doi.org/10.15468/zsjppb accessed via GBIF.org on 2023-06-03.

Egger C S, valdenar (2019). Two new species of Spider Wasps (Hymenoptera, Pompilidae), Ctenagenia pagliano from Lebanon and Evagetes liane from Canary Islands. Plazi.org taxonomic treatments database. Checklist dataset https://doi.org/10.5281/zenodo.3742968 accessed via GBIF.org on 2023-06-03.

Espadaler X, Klingenberg C (2007). The ants of El Hierro (Canary Islands).. Plazi.org taxonomic treatments database. Checklist dataset https://doi.org/10.15468/9ookiz accessed via GBIF.org on 2023-06-03.

Esser J, valdenar (2019). Anmerkungen zur Gattung Dapsa L, 1829 (Coleoptera, Endomychidae). Plazi.org taxonomic treatments database. Checklist dataset https://doi.org/10.5281/zenodo.3741910 accessed via GBIF.org on 2023-06-03.

Estonian Naturalists’ Society. Estonian Naturalists’ Society. Occurrence dataset https://doi.org/10.15468/bmk3ab accessed via GBIF.org on 2023-06-03.

European Bioinformatics Institute (EMBL-EBI), GBIF Helpdesk (2023). INSDC Host Organism Sequences. Version 1.34. European Nucleotide Archive (EMBL-EBI). Metadata dataset https://doi.org/10.15468/e97kmy accessed via GBIF.org on 2023-06-03.

European Bioinformatics Institute (EMBL-EBI), GBIF Helpdesk (2023). INSDC Sequences. Version 1.34. European Nucleotide Archive (EMBL-EBI). Metadata dataset https://doi.org/10.15468/sbmztx accessed via GBIF.org on 2023-06-03.

Eusko Jaurlaritza - Gobierno Vasco, Iturribarria M (2022). Coleópteros del País Vasco - Sistema de Información de la Naturaleza de Euskadi. Basque Government. Occurrence dataset https://doi.org/10.15468/p4fda2 accessed via GBIF.org on 2023-06-03.

Eusko Jaurlaritza - Gobierno Vasco, Iturribarria M (2022). Detección y distribución de coleópteros saproxílicos de interés comunitario en cuatro espacios de la red Natura 2000 del País Vasco: Arno, Gárate-Santa Bárbara, Sierra Salvada y Urkiola - Sistema de Información de la Naturaleza de Euskadi. Basque Government. Occurrence dataset https://doi.org/10.15468/gn74jr accessed via GBIF.org on 2023-06-03.

Favret C (2020). Ouellet-Robert Entomological Collection (QMOR) - Specimens. Version 12.5. Université de Montréal Biodiversity Centre. Occurrence dataset https://doi.org/10.5886/qwvt63fz accessed via GBIF.org on 2023-06-03.

Fernández Blanco L M (2020). Colecciones Zoológicas de la Universidad de León, Colección de Insectos. Zoological collections of the University of Leon (CZULE), University of Leon. Occurrence dataset https://doi.org/10.15468/y9uegn accessed via GBIF.org on 2023-06-03.

Ferreira S (2023). The InBIO Barcoding Initiative Database: contribution to the knowledge on DNA barcodes of cuckoo wasps, with the description of new species from the Iberian Peninsula (Hymenoptera, Chrysididae). Biodiversity Data Journal. Checklist dataset https://doi.org/10.3897/bdj.11.e98743 accessed via GBIF.org on 2023-06-03.

Ferreira S, Andrade R, Gonçalves A R, Sousa P, Beja P (2021). The InBIO Barcoding Initiative Database: Diptera 01. Version 1.6. CIBIO (Research Center in Biodiversity and Genetic Resources) Portugal. Occurrence dataset https://doi.org/10.15468/q1bvt3 accessed via GBIF.org on 2023-06-03.

Ferreira S, Bohn H, Sousa P, Beja P (2020). The InBIO Barcoding Initiative Database: Blattodea 01. Version 1.2. CIBIO (Research Center in Biodiversity and Genetic Resources) Portugal. Occurrence dataset https://doi.org/10.15468/m7f8sg accessed via GBIF.org on 2023-06-03.

Ferreira S, Oliveira D, Corley M, da Silva L P, Sousa P, Beja P (2021). The InBIO Barcoding Initiative Database: Portuguese Mantises (Mantodea). Version 1.2. CIBIO (Research Center in Biodiversity and Genetic Resources) Portugal. Occurrence dataset https://doi.org/10.15468/ux2yhn accessed via GBIF.org on 2023-06-03.

Ferreira S, Oliveira D, Lopes P B, Sousa P, Beja P (2020). The InBIO Barcoding Initiative Database: Portuguese Earwigs (Dermaptera). Version 1.5. CIBIO (Research Center in Biodiversity and Genetic Resources) Portugal. Occurrence dataset https://doi.org/10.15468/jups46 accessed via GBIF.org on 2023-06-03.

Ferreira S, Oliveira D, Sousa P, Beja P (2021). The InBIO Barcoding Initiative Database: Wesmaelius (Kimninsia) nervosus (Neuroptera, Hemerobiidae): a new species of brown lacewing for the Portuguese fauna. Version 1.2. CIBIO (Research Center in Biodiversity and Genetic Resources) Portugal. Occurrence dataset https://doi.org/10.15468/s36xpq accessed via GBIF.org on 2023-06-03.

Ferreira S, Oosterbroek P, Starý J, Alves Mata V, da Silva L P, Sousa P, Beja P (2021). The InBIO Barcoding Initiative Database: DNA barcodes of Portuguese Diptera 02 - Limoniidae, Pediciidae and Tipulidae. CIBIO (Research Center in Biodiversity and Genetic Resources) Portugal. Occurrence dataset https://doi.org/10.15468/f6256w accessed via GBIF.org on 2023-06-03.

Ferreira S, Oosterbroek P, Starý J, Andrade R, Hancock E G, Mata V A, da Silva L P, Lopes P B, Corley M, Sousa P, Beja P (2021). The InBIO Barcoding Initiative Database: Diptera 03 - Further records on Portuguese Limoniidae, Pediciidae and Tipulidae. CIBIO (Research Center in Biodiversity and Genetic Resources) Portugal. Occurrence dataset https://doi.org/10.15468/759dz2 accessed via GBIF.org on 2023-06-03.

Ferreira S, P. da Silva L, Sousa P, Beja P (2021). The InBIO Barcoding Initiative Database: Portuguese Stag beetles (Coleoptera, Lucanidae). Version 1.5. CIBIO (Research Center in Biodiversity and Genetic Resources) Portugal. Occurrence dataset https://doi.org/10.15468/7559ru accessed via GBIF.org on 2023-06-03.

Ferreira S, Paupério J, Grosso-Silva J M, Sousa P, Beja P (2021). The InBIO Barcoding Initiative Database: DNA barcoding of Sialis sp. (Megaloptera) in Portugal: the missing tool to species identification. Version 1.2. CIBIO (Research Center in Biodiversity and Genetic Resources) Portugal. Occurrence dataset https://doi.org/10.15468/x7yk7m accessed via GBIF.org on 2023-06-03.

Ferreira S, Tierno de Figueroa J M, Martins F M S, Veríssimo J, Quaglietta L, Grosso-Silva J M, Lopes P B, Sousa P, Beja P (2021). The InBIO Barcoding Initiative Database: contribution to the knowledge on DNA barcodes of Iberian Plecoptera. Version 1.6. CIBIO (Research Center in Biodiversity and Genetic Resources) Portugal. Occurrence dataset https://doi.org/10.15468/3vkdr2 accessed via GBIF.org on 2023-06-03.

Finnish Biodiversity Information Facility (2023). Coleoptera Palaearctic (Luomus) (PC). Occurrence dataset https://doi.org/10.15468/vvf3sv accessed via GBIF.org on 2023-06-03.

Finnish Biodiversity Information Facility (2023). Coleoptera World (Luomus) (EC). Occurrence dataset https://doi.org/10.15468/4fnqq9 accessed via GBIF.org on 2023-06-03.

Finnish Biodiversity Information Facility (2023). Diptera Eastern Fennoscandia (MZH/Luomus). Occurrence dataset https://doi.org/10.15468/rrua9e accessed via GBIF.org on 2023-06-03.

Finnish Biodiversity Information Facility (2023). Diptera Holarctic (MZH/Luomus). Occurrence dataset https://doi.org/10.15468/jkhnnu accessed via GBIF.org on 2023-06-03.

Finnish Biodiversity Information Facility (2023). Diptera R. Frey (MZH/Luomus). Occurrence dataset https://doi.org/10.15468/fey985 accessed via GBIF.org on 2023-06-03.

Finnish Biodiversity Information Facility (2023). Entomological slide collection (Luomus). Occurrence dataset https://doi.org/10.15468/5sgp2w accessed via GBIF.org on 2023-06-03.

Finnish Biodiversity Information Facility (2023). Hatikka.fi observations. Occurrence dataset https://doi.org/10.15468/te1t6l accessed via GBIF.org on 2023-06-03.

Finnish Biodiversity Information Facility (2023). Hemiptera Eastern Fennoscandia (Luomus). Occurrence dataset https://doi.org/10.15468/qf5t9a accessed via GBIF.org on 2023-06-03.

Finnish Biodiversity Information Facility (2023). Hemiptera World (Luomus). Occurrence dataset https://doi.org/10.15468/vv7cpn accessed via GBIF.org on 2023-06-03.

Finnish Biodiversity Information Facility (2023). Hymenoptera World (Luomus). Occurrence dataset https://doi.org/10.15468/d5q2vn accessed via GBIF.org on 2023-06-03.

Finnish Biodiversity Information Facility (2023). Invertebrata varia (Luomus). Occurrence dataset https://doi.org/10.15468/h37nwv accessed via GBIF.org on 2023-06-03.

Finnish Biodiversity Information Facility (2023). KUO Lepidoptera collections (world). Occurrence dataset https://doi.org/10.15468/hm6zdb accessed via GBIF.org on 2023-06-03.

Finnish Biodiversity Information Facility (2023). Lajitietokeskus/FinBIF - Notebook, general observations. Occurrence dataset https://doi.org/10.15468/4g56tp accessed via GBIF.org on 2023-06-03.

Finnish Biodiversity Information Facility (2023). Lepidoptera Eastern Fennoscandia (Luomus) (LEPFEN). Occurrence dataset https://doi.org/10.15468/vcddkt accessed via GBIF.org on 2023-06-03.

Finnish Biodiversity Information Facility (2023). Lepidoptera World (Luomus) (LEPW). Occurrence dataset https://doi.org/10.15468/mcarqb accessed via GBIF.org on 2023-06-03.

Finnish Biodiversity Information Facility (2023). Minor insect orders (Luomus). Occurrence dataset https://doi.org/10.15468/6n5efa accessed via GBIF.org on 2023-06-03.

Finnish Biodiversity Information Facility (2023). Regional Museum of Lapland (LMM). Occurrence dataset https://doi.org/10.15468/xzuqg5 accessed via GBIF.org on 2023-06-03.

Finnish Biodiversity Information Facility (2023). ZMAA Insect collections of Åbo Akademi. Occurrence dataset https://doi.org/10.15468/m6dets accessed via GBIF.org on 2023-06-03.

Finnish Biodiversity Information Facility (2023). ZMAA Type specimen collections of Åbo Akademi. Occurrence dataset https://doi.org/10.15468/juqbqr accessed via GBIF.org on 2023-06-03.

Finnish Biodiversity Information Facility (2023). ZMUO Common invertebrate collection. Occurrence dataset https://doi.org/10.15468/8zyyzk accessed via GBIF.org on 2023-06-03.

Fisher B, Fong J (2023). AntWeb. California Academy of Sciences. Occurrence dataset https://doi.org/10.15468/wqmjjt accessed via GBIF.org on 2023-06-03.

Fogh Nielsen O, Skipper L (2023). Danish Lacewings (Neuroptera). Version 1.3. Danish Biodiversity Information Facility. Occurrence dataset https://doi.org/10.15468/nago7r accessed via GBIF.org on 2023-06-03.

Fortuño Estrada P (2020). Efectos del Cambio Ambiental en las comunidades de organismos de los ríos mediterráneos. Version 2.10. Freshwater Ecology, Hydrology and Management (F.E.H.M.) Research Group - Universitat de Barcelona. Sampling event dataset https://doi.org/10.15470/yt2uex accessed via GBIF.org on 2023-06-03.

Gaiani M (2019). Insect Occurrence Data from MIZA. Version 1.1. Universidad Central de Venezuela. Occurrence dataset https://doi.org/10.15468/ah3is7 accessed via GBIF.org on 2023-06-03.

Gall L (2023). Entomology Division, Yale Peabody Museum. Yale University Peabody Museum. Occurrence dataset https://doi.org/10.15468/95waq3 accessed via GBIF.org on 2023-06-03.

Garcia 1 R, Andujarx C, Oromi P, Emerson B, Lopez H, pensoft (2021). Three new subterranean species of Baezia (Curculionidae, Molytinae) for the Canary Islands. Plazi.org taxonomic treatments database. Checklist dataset https://doi.org/10.3897/subtbiol.38.61733 accessed via GBIF.org on 2023-06-03.

García M, Pérez G, Portillo M, plazi (2017). Taxonomic review of the species of Andrenosoma (Diptera: Asilidae) in Spain and description of a new species. Plazi.org taxonomic treatments database. Checklist dataset https://doi.org/10.11646/zootaxa.4299.3.8 accessed via GBIF.org on 2023-06-03.

García-Barros E (2020). Universidad Autónoma de Madrid, Madrid; Lepidópteros. Dep. Biology, Univ. Autónoma de Madrid. Occurrence dataset https://doi.org/10.15468/auzyaa accessed via GBIF.org on 2023-06-03.

Gaspar H, Loureiro J, Castro S (2020). Brown Marmorated Stink Bug (Halyomorpha halys) in Portugal. Version 1.1. CFE - Centre for Functional Ecology, Department of Life Sciences, University of Coimbra. Occurrence dataset https://doi.org/10.15468/s5kqb5 accessed via GBIF.org on 2023-06-03.

Generalitat de Catalunya ., Generalitat de Catalunya (2022). Citacions biodiversitat Espais Naturals Protecció Especial. Generalitat de Catalunya. Occurrence dataset https://doi.org/10.15470/m5ic1c accessed via GBIF.org on 2023-06-03.

Generalitat Valenciana (2023). Banco de Datos de la Biodiversidad de la Comunitat Valenciana. Biodiversity data bank of Generalitat Valenciana. Occurrence dataset https://doi.org/10.15468/b4yqdy accessed via GBIF.org on 2023-06-03.

Germann C, Borovec R, Braunert C, plazi (2015). Four new Entiminae from the Mediterranean region (Coleoptera: Curculionidae: Entiminae: Phyllobiini, Trachyphloeini), with additional data on the distribution of some poorly known species. Plazi.org taxonomic treatments database. Checklist dataset https://doi.org/10.11646/zootaxa.4040.3.6 accessed via GBIF.org on 2023-06-03.

Germann C, carolina (2020). On Anchonidium Bedel, 1884 sensu stricto, with descriptions of two new species from the Iberian peninsula (Coleoptera, Curculionidae: Molytinae). Plazi.org taxonomic treatments database. Checklist dataset https://doi.org/10.15468/vbhvcg accessed via GBIF.org on 2023-06-03.

Gess D S, Ranwashe F (2017). AM: Terrestrial Insect Collections (1896-2000). Version 1.5. South African National Biodiversity Institute. Occurrence dataset https://doi.org/10.15468/nlkj0p accessed via GBIF.org on 2023-06-03.

Gibbs D, plazi (2014). A world revision of the bee fly tribe Usiini (Diptera, Bombyliidae) Part 2: Usia sensu stricto. Plazi.org taxonomic treatments database. Checklist dataset https://doi.org/10.11646/zootaxa.3799.1.1 accessed via GBIF.org on 2023-06-03.

Gil Tapetado D (2019). BV Insectarium Virtual. Version 1.4. Fotografía y Biodiversidad. Occurrence dataset https://doi.org/10.15470/umrxis accessed via GBIF.org on 2023-06-03.

Gnezdilov V M, Bouhachem S, Konstantinov F V, plazi (2019). New records for the genus Issus Fabricius (Hemiptera: Auchenorrhyncha: Fulgoroidea: Issidae) from northern Africa and Spain. Plazi.org taxonomic treatments database. Checklist dataset https://doi.org/10.11646/zootaxa.4613.3.7 accessed via GBIF.org on 2023-06-03.

Gonçalves A R, Grootaert P, Andrade R, Paulo O S, Mengual X, plazi (2021). Revision of the morphology, phylogenetic relationships, behaviour and diversity of the Iberian and Italian ant-like Tachydromia Meigen, 1803 (Diptera: Hybotidae). Plazi.org taxonomic treatments database. Checklist dataset https://doi.org/10.5852/ejt.2021.732.1213 accessed via GBIF.org on 2023-06-03.

Grant S, Webbink K, Turcatel M, Shuman R (2023). Field Museum of Natural History (Zoology) Insect, Arachnid and Myriapod Collection. Version 12.53. Field Museum. Occurrence dataset https://doi.org/10.15468/0ywfpc accessed via GBIF.org on 2023-06-03.

Gratacós Teixidor G, Feo Quer C (2019). Museu Darder de Banyoles. Version 2.1. Darder Natural History Museum of Banyoles. Occurrence dataset https://doi.org/10.15470/wxaleu accessed via GBIF.org on 2023-06-03.

Grégoire Taillefer A, Boucher S (2020). Lyman Entomological Museum (LEMQ). Version 24.6. McGill University. Occurrence dataset https://doi.org/10.5886/q79vhp1e accessed via GBIF.org on 2023-06-03.

Grinter C, Diaz-Bastin R, Fong J (2023). CAS Entomology Type (TYPE). Version 1.190. California Academy of Sciences. Occurrence dataset https://doi.org/10.15468/gak5hc accessed via GBIF.org on 2023-06-03.

Grinter C, Fong J (2023). CAS Entomology (ENT). Version 6.229. California Academy of Sciences. Occurrence dataset https://doi.org/10.15468/3ibpmo accessed via GBIF.org on 2023-06-03.

Grkovic A, Smit J, Radenkovic S, Vujic A, Steenis J V, pensoft (2019). Two new European long-legged hoverfly species of the Eumerusbinominatus species subgroup (Diptera, Syrphidae). Plazi.org taxonomic treatments database. Checklist dataset https://doi.org/10.3897/zookeys.858.34663 accessed via GBIF.org on 2023-06-03.

Gross J, Oboyski P (2023). Essig Museum of Entomology. Version 121.291. Berkeley Natural History Museums. Occurrence dataset https://doi.org/10.15468/0saucj accessed via GBIF.org on 2023-06-03.

Hagiwara K, Uchifune T (2023). Insect Collection of Yokosuka City Museum. National Museum of Nature and Science, Japan. Occurrence dataset https://doi.org/10.15468/24xqf2 accessed via GBIF.org on 2023-06-03.

Hårsaker K, Aspaas A M, Dolmen D, Ekrem T, Stur E, Ødegaard F, Aagaard K, Finstad A G (2023). Terrestrial and limnic invertebrates systematic collection, NTNU University Museum. Version 1.1176. Norwegian University of Science and Technology. Occurrence dataset https://doi.org/10.15468/fsreqb accessed via GBIF.org on 2023-06-03.

Harvard University M, Morris P J (2023). Museum of Comparative Zoology, Harvard University. Version 162.374. Museum of Comparative Zoology, Harvard University. Occurrence dataset https://doi.org/10.15468/p5rupv accessed via GBIF.org on 2023-06-03.

Hashimoto Y (2021). Entomological Specimens of Museum of Nature and Human Activities, Hyogo Pref., Japan. Version 2.3. National Institute of Genetics, ROIS. Occurrence dataset https://doi.org/10.15468/bqkybh accessed via GBIF.org on 2023-06-03.

Haus der Natur Salzburg, Museum für Natur und Technik. Biodiversitätsdatenbank Salzburg. Occurrence dataset https://doi.org/10.15468/3pf855 accessed via GBIF.org on 2023-06-03.

Hernando C, Pérez T, plazi (2023). Tychobythinus antojandro sp. nov., a new troglobitic species of Pselaphinae (Coleoptera: Staphylinidae) from Andalusia, Spain. Plazi.org taxonomic treatments database. Checklist dataset https://doi.org/10.15468/hkne4y accessed via GBIF.org on 2023-06-03.

Hillert O, Arnone M, Král D, Massa B, marcus (2016). The genusBolbelasmus in the western and southern regions of the Mediterranean Basin (Coleoptera: Geotrupidae: Bolboceratinae). Plazi.org taxonomic treatments database. Checklist dataset https://doi.org/10.15468/rstbad accessed via GBIF.org on 2023-06-03.

Holston K (2023). Entomological Collections (NHRS), Swedish Museum of Natural History (NRM). Version 26.827. Swedish Museum of Natural History. Occurrence dataset https://doi.org/10.15468/fpzyjx accessed via GBIF.org on 2023-06-03.

ICoMM MICROBIS https://doi.org/10.15468/wi6v9k accessed via GBIF.org on 2023-06-03.

Ikerd H (2019). Bee Biology and Systematics Laboratory. USDA-ARS Pollinating Insect-Biology, Management, Systematics Research. Occurrence dataset https://doi.org/10.15468/anyror accessed via GBIF.org on 2023-06-03.

iNaturalist contributors, iNaturalist (2023). iNaturalist Research-grade Observations. iNaturalist.org. Occurrence dataset https://doi.org/10.15468/ab3s5x accessed via GBIF.org on 2023-06-03.

Inventaire National du Patrimoine Naturel (2017). REBENT - Réseau national de surveillance des biocénoses benthiques côtières. UMS PatriNat (OFB-CNRS-MNHN), Paris. Occurrence dataset https://doi.org/10.15468/h74fvv accessed via GBIF.org on 2023-06-03.

Inventaire National du Patrimoine Naturel (2018). Données d'occurrences issues des Formulaires standards de données des sites Natura 2000 - Données d'occurrence Espèces issues de la base Natura 2000 : espèces d'intérêt communautaire et autres espèces remarquables renseignées dans les FSD. UMS PatriNat (OFB-CNRS-MNHN), Paris. Occurrence dataset https://doi.org/10.15468/g2ptuw accessed via GBIF.org on 2023-06-03.

Inventaire National du Patrimoine Naturel (2018). Données du Parc national des Pyrénées - Observations occasionnelles Parc national des Pyrénées. UMS PatriNat (OFB-CNRS-MNHN), Paris. Occurrence dataset https://doi.org/10.15468/g3k5au accessed via GBIF.org on 2023-06-03.

Inventaire National du Patrimoine Naturel (2018). Dreal Occitanie- Base Biodiv SINP - Données faune pour le rapportage européen DHFF-DO 2018. UMS PatriNat (OFB-CNRS-MNHN), Paris. Occurrence dataset https://doi.org/10.15468/vn42mk accessed via GBIF.org on 2023-06-03.

Inventaire National du Patrimoine Naturel (2019). CardObs : Observations naturalistes issues de l'outil CardObs - Données liées à des déterminations effectuées par DUSOULIER François. UMS PatriNat (OFB-CNRS-MNHN), Paris. Occurrence dataset https://doi.org/10.15468/jd6c8k accessed via GBIF.org on 2023-06-03.

Inventaire National du Patrimoine Naturel (2020). Appui technique et scientifique auprès de l'Ecole d'Ingénieurs de Purpan (EIP) - Données CEN M-P EIP. UMS PatriNat (OFB-CNRS-MNHN), Paris. Occurrence dataset https://doi.org/10.15468/gdvjng accessed via GBIF.org on 2023-06-03.

Inventaire National du Patrimoine Naturel (2020). Atlas des papillons de jour et des libellules du Languedoc-Roussillon - Données CEN L-R papillons/libellule. UMS PatriNat (OFB-CNRS-MNHN), Paris. Occurrence dataset https://doi.org/10.15468/u8js9d accessed via GBIF.org on 2023-06-03.

Inventaire National du Patrimoine Naturel (2020). Atlas écologique régional des papillons de jour et zygènes (Lépidoptères) de Midi-Pyrénées - Données de l'Atlas écologique régional des papillons de jour et zygènes. UMS PatriNat (OFB-CNRS-MNHN), Paris. Occurrence dataset https://doi.org/10.15468/yw2sr2 accessed via GBIF.org on 2023-06-03.

Inventaire National du Patrimoine Naturel (2020). Données de présence collectées sur les odonates. Version 1.1. UMS PatriNat (OFB-CNRS-MNHN), Paris. Occurrence dataset https://doi.org/10.15468/xgnt9v accessed via GBIF.org on 2023-06-03.

Inventaire National du Patrimoine Naturel (2020). Données de présence collectées sur les rhopalocères. Version 1.1. UMS PatriNat (OFB-CNRS-MNHN), Paris. Occurrence dataset https://doi.org/10.15468/nhbth7 accessed via GBIF.org on 2023-06-03.

Inventaire National du Patrimoine Naturel (2020). Données de rhopalocères récoltées par le CEN Aquitaine en 2011 et 2012. Version 1.1. UMS PatriNat (OFB-CNRS-MNHN), Paris. Occurrence dataset https://doi.org/10.15468/lbhpuc accessed via GBIF.org on 2023-06-03.

Inventaire National du Patrimoine Naturel (2020). Programmes de connaissance sur les forêts d'Occitanie - Données vieilles forêts. UMS PatriNat (OFB-CNRS-MNHN), Paris. Occurrence dataset https://doi.org/10.15468/8ykamg accessed via GBIF.org on 2023-06-03.

Inventaire National du Patrimoine Naturel (2020). Rapportage 2001-2006 au titre de la directive Habitats-Faune-Flore - Données de synthèse Espèces issues du rapportage au titre de la directive Habitats - période 2001-2006. UMS PatriNat (OFB-CNRS-MNHN), Paris. Occurrence dataset https://doi.org/10.15468/j4mp83 accessed via GBIF.org on 2023-06-03.

Inventaire National du Patrimoine Naturel (2020). Rapportage 2007-2012 au titre de la directive Habitats-Faune-Flore - Données de synthèse Espèces issues du rapportage au titre de la directive Habitats - période 2007-2012. UMS PatriNat (OFB-CNRS-MNHN), Paris. Occurrence dataset https://doi.org/10.15468/tugutg accessed via GBIF.org on 2023-06-03.

Inventaire National du Patrimoine Naturel (2020). Rapportage 2013-2018 au titre de la directive Habitats-Faune-Flore - Données de synthèse Espèces issues du rapportage au titre de la directive Habitats - période 2013-2018. UMS PatriNat (OFB-CNRS-MNHN), Paris. Occurrence dataset https://doi.org/10.15468/ubgty4 accessed via GBIF.org on 2023-06-03.

Inventaire National du Patrimoine Naturel (2020). Regroupement de données "papillons menacés" collectées par les réseaux entre 1990 et 2012. Version 1.1. UMS PatriNat (OFB-CNRS-MNHN), Paris. Occurrence dataset https://doi.org/10.15468/tk0qos accessed via GBIF.org on 2023-06-03.

Inventaire National du Patrimoine Naturel (2021). Mutualisation des données naturalistes pour le rapportage Natura2000 en 2017 - Synthèse 10x10km de la faune sauvage des Directives Oiseaux et Habitats-Faune-Flore recensée de 2013 à 2017. UMS PatriNat (OFB-CNRS-MNHN), Paris. Occurrence dataset https://doi.org/10.15468/2vzmn3 accessed via GBIF.org on 2023-06-03.

Inventaire National du Patrimoine Naturel (2021). Saisie de données naturalistes d'observateurs indépendants sur la plateforme de l'Observatoire FAUNA - Données naturalistes de Sylvain BONIFAIT. UMS PatriNat (OFB-CNRS-MNHN), Paris. Occurrence dataset https://doi.org/10.15468/cf2c4d accessed via GBIF.org on 2023-06-03.

Inventaire National du Patrimoine Naturel (2022). Atlas de la Biodiversité Communale - [ABC] - PNP - Inventaire Faune/Flore/Fonge. UMS PatriNat (OFB-CNRS-MNHN), Paris. Occurrence dataset https://doi.org/10.15468/h6zahw accessed via GBIF.org on 2023-06-03.

Inventaire National du Patrimoine Naturel (2022). Autorisation 2019-03 - SHHNH. UMS PatriNat (OFB-CNRS-MNHN), Paris. Occurrence dataset https://doi.org/10.15468/38e5zg accessed via GBIF.org on 2023-06-03.

Inventaire National du Patrimoine Naturel (2022). Données d'observation de la Faune, de la Flore et de la Fonge - [HORS_PROTOCOLE] - Contributions à la connaissance sur la biodiversité du Parc National des Pyrénées. UMS PatriNat (OFB-CNRS-MNHN), Paris. Occurrence dataset https://doi.org/10.15468/u5z6yz accessed via GBIF.org on 2023-06-03.

Inventaire National du Patrimoine Naturel (2022). ONF - Observations et inventaires MOA ONF - ONF - Observations opportunistes et inventaires. UMS PatriNat (OFB-CNRS-MNHN), Paris. Occurrence dataset https://doi.org/10.15468/tgap4x accessed via GBIF.org on 2023-06-03.

Iturribarria M (2018). Programa de seguimiento de mariposas diurnas del País Vasco. Version 2.5. Basque Government. Occurrence dataset https://doi.org/10.15470/7wmd2y accessed via GBIF.org on 2023-06-03.

Jałoszyński P, Struyve T, plazi (2016). A new subterranean Iberian Cephennium with unusually modified metatrochanters (Coleoptera, Staphylinidae, Scydmaeninae). Plazi.org taxonomic treatments database. Checklist dataset https://doi.org/10.11646/zootaxa.4189.2.5 accessed via GBIF.org on 2023-06-03.

Johannessen L E, Johnsen A (2023). NHMO DNA Bank Arthropod collection. Version 1.165. University of Oslo. Occurrence dataset https://doi.org/10.15468/vxhrbz accessed via GBIF.org on 2023-06-03.

Johnson N (2019). Cleveland Museum of Natural History. Museum of Biological Diversity, The Ohio State University. Occurrence dataset https://doi.org/10.15468/bmfgag accessed via GBIF.org on 2023-06-03.

Johnson N, Cora J. C.A. Triplehorn Insect Collection (OSUC), Ohio State University. Museum of Biological Diversity, The Ohio State University. Occurrence dataset https://doi.org/10.15468/efb17f accessed via GBIF.org on 2023-06-03.

Journals P (2011). Western Palaearctic Ectoedemia (Zimmermannia) Hering and Ectoedemia Busck s. str. (Lepidoptera: Nepticulidae): five new species and new data on distribution, hostplants and recognition. ZooKeys. Occurrence dataset https://doi.org/10.15468/nmwhaj accessed via GBIF.org on 2023-06-03.

Julien Bariteaud N, inconnu N, Inventaire National du Patrimoine Naturel (2020). Données d'invertébrés. Version 1.1. UMS PatriNat (OFB-CNRS-MNHN), Paris. Occurrence dataset https://doi.org/10.15468/dpuqh5 accessed via GBIF.org on 2023-06-03.

Karsholt O (2014). Bryotropha (Lepidoptera: Gelechiidae) in Western Palaearctic. Natural History Museum of Denmark. Occurrence dataset https://doi.org/10.15468/a3lljm accessed via GBIF.org on 2023-06-03.

Kasparek M, plazi (2020). Revision of the Palaearctic Trachusa interrupta species complex (Apoidea: Anthidiini) with description of four new species. Plazi.org taxonomic treatments database. Checklist dataset https://doi.org/10.11646/zootaxa.4728.1.1 accessed via GBIF.org on 2023-06-03.

Kasparek M, Wood T, Ferreira S, Benarfa N, plazi (2023). Taxonomic status of the disjunct populations of the resin bee Anthidiellum breviusculum (Pérez, 1890) s. l. in the Mediterranean (Apoidea: Anthidiini). Plazi.org taxonomic treatments database. Checklist dataset https://doi.org/10.15468/7r33cc accessed via GBIF.org on 2023-06-03.

Keller O, Schnepp K E, Ashman K L, Turnbow R H, Skelley P E, plazi (2020). An annotated catalog of the type material of Adephaga and Myxophaga (Coleoptera) deposited in the Florida State Collection of Arthropods in Gainesville, Florida, United States of America. Plazi.org taxonomic treatments database. Checklist dataset https://doi.org/10.11646/zootaxa.4744.1.1 accessed via GBIF.org on 2023-06-03.

Klompen H, Johnson N (2018). Ohio State Acarology Laboratory (OSAL), Ohio State University. Museum of Biological Diversity, The Ohio State University. Occurrence dataset https://doi.org/10.15468/dzwr2y accessed via GBIF.org on 2023-06-03.

Kolcsár L (2015). A new species and new records of Molophilus Curtis, 1833 (Diptera: Limoniidae) from the Western Palaearctic Region. Biodiversity Data Journal. Checklist dataset https://doi.org/10.3897/bdj.3.e5466 accessed via GBIF.org on 2023-06-03.

Kolcsár L (2021). Contribution to the knowledge of Limoniidae (Diptera: Tipuloidea): first records of 244 species from various European countries. Biodiversity Data Journal. Checklist dataset https://doi.org/10.3897/bdj.9.e67085 accessed via GBIF.org on 2023-06-03.

Konvička O, Brustel H, plazi (2021). Description of Marolia alicantina sp. nov. (Coleoptera: Melandryidae) from Spain and new distribution records of Marolia species. Plazi.org taxonomic treatments database. Checklist dataset https://doi.org/10.11646/zootaxa.4920.3.6 accessed via GBIF.org on 2023-06-03.

Korneyev S V, Korneyev V A, plazi (2019). Revision of the Old World species of the genus Tephritis (Diptera, Tephritidae) with a pair of isolated apical spots. Plazi.org taxonomic treatments database. Checklist dataset https://doi.org/10.11646/zootaxa.4584.1.1 accessed via GBIF.org on 2023-06-03.

Kuntsi S, Koskela T (2014). Invertebrate collection of Jyväskylä University Museum. Jyväskylä University Museum - The Section of Natural Sciences. Occurrence dataset https://doi.org/10.15468/55lrhm accessed via GBIF.org on 2023-06-03.

Kurina O. Estonian University of Life Sciences Institute of Agricultural and Environmental Sciences Entomological Collection. Estonian University of Life Sciences. Occurrence dataset https://doi.org/10.15468/qn6223 accessed via GBIF.org on 2023-06-03.

Kvifte G (2016). New records of Trichoceridae (Diptera) from the island of Mallorca . Biodiversity Data Journal. Checklist dataset https://doi.org/10.3897/bdj.4.e7610 accessed via GBIF.org on 2023-06-03.

Lair X, Ropars L, Skevington J H, Kelso S, Geslin B, Minssieux E, Nève G, plazi (2022). Revision of the genus Pelecocera Meigen, 1822 (Diptera: Syrphidae) from France taxonomy, ecology and distribution. Plazi.org taxonomic treatments database. Checklist dataset https://doi.org/10.15468/5b5ctg accessed via GBIF.org on 2023-06-03.

Lamelas-Lopez L, Borges P A V, Lopes D H (2023). Inventory of Arthropod pests in Azorean orchards: The project CUARENTAGRI. Version 1.6. Universidade dos Açores. Sampling event dataset https://doi.org/10.15468/cpergb accessed via GBIF.org on 2023-06-03.

Larrivée M (2020). Insectarium de Montréal (IMQC). Version 2.5. Insectarium de Montréal. Occurrence dataset https://doi.org/10.5886/i6z1vo accessed via GBIF.org on 2023-06-03.

Larrivée M, McFarland K, Zhang X, Prudic K, Solis R, Bunsen M, Kerr J (2023). eButterfly Surveys. Version 1.440. Vermont Center for Ecostudies. Sampling event dataset https://doi.org/10.15468/ykxm8x accessed via GBIF.org on 2023-06-03.

Leibniz Institute for the Analysis of Biodiversity Change (LIB) (2018). ZFMK DORSA. Occurrence dataset https://doi.org/10.15468/iihdbo accessed via GBIF.org on 2023-06-03.

Leibniz Institute for the Analysis of Biodiversity Change (LIB) (2018). ZFMK Sound Laboratory. Occurrence dataset https://doi.org/10.15468/yhye2h accessed via GBIF.org on 2023-06-03.

Leibniz Institute for the Analysis of Biodiversity Change (LIB). ZFMK Blattodea collection. Occurrence dataset https://doi.org/10.15468/f85d26 accessed via GBIF.org on 2023-06-03.

Leibniz Institute for the Analysis of Biodiversity Change (LIB). ZFMK Dermaptera collection. Occurrence dataset https://doi.org/10.15468/4qgqjz accessed via GBIF.org on 2023-06-03.

Leibniz Institute for the Analysis of Biodiversity Change (LIB). ZFMK Diptera Collection. Occurrence dataset https://doi.org/10.15468/8mgh4p accessed via GBIF.org on 2023-06-03.

Leibniz Institute for the Analysis of Biodiversity Change (LIB). ZFMK Heteroptera collection. Occurrence dataset https://doi.org/10.15468/eowwbl accessed via GBIF.org on 2023-06-03.

Leibniz Institute for the Analysis of Biodiversity Change (LIB). ZFMK Homoptera collection. Occurrence dataset https://doi.org/10.15468/qkqha4 accessed via GBIF.org on 2023-06-03.

Leibniz Institute for the Analysis of Biodiversity Change (LIB). ZFMK Hymenoptera collection. Occurrence dataset https://doi.org/10.15468/dsd416 accessed via GBIF.org on 2023-06-03.

Leibniz Institute for the Analysis of Biodiversity Change (LIB). ZFMK Mantodea collection. Occurrence dataset https://doi.org/10.15468/bscmew accessed via GBIF.org on 2023-06-03.

Leibniz Institute for the Analysis of Biodiversity Change (LIB). ZFMK Orthoptera collection. Occurrence dataset https://doi.org/10.15468/t3yz95 accessed via GBIF.org on 2023-06-03.

Leibniz Institute for the Analysis of Biodiversity Change (LIB). ZFMK Phtiraptera collection. Occurrence dataset https://doi.org/10.15468/43wtgh accessed via GBIF.org on 2023-06-03.

Leibniz Institute for the Analysis of Biodiversity Change (LIB). ZFMK Siphonaptera collection. Occurrence dataset https://doi.org/10.15468/4rzt84 accessed via GBIF.org on 2023-06-03.

Lennuk L. Estonian Museum of Natural History Department of Zoology. Estonian Museum of Natural History. Occurrence dataset https://doi.org/10.15468/98cxtc accessed via GBIF.org on 2023-06-03.

Liston A, Prous M, Vårdal H, felipe (2019). A review of West Palaearctic Hoplocampa species, focussing on Sweden (Hymenoptera, Tenthredinidae). Plazi.org taxonomic treatments database. Checklist dataset https://doi.org/10.11646/zootaxa.4615.1.1 accessed via GBIF.org on 2023-06-03.

Liston A, Prous M, Vårdal H, plazi (2019). The West Palaearctic Pseudodineura and Endophytus species (Hymenoptera, Tenthredinidae). Plazi.org taxonomic treatments database. Checklist dataset https://doi.org/10.11646/zootaxa.4614.3.5 accessed via GBIF.org on 2023-06-03.

Liu G, Disney R H L, plazi (2022). Revision of the ant-parasitizing genus Pseudacteon Coquillett (Diptera, Phoridae) from China. Plazi.org taxonomic treatments database. Checklist dataset https://doi.org/10.15468/s6pke8 accessed via GBIF.org on 2023-06-03.

Lobo J M (2020). BANDAPHO, BAse de DAtos sobre Aphodiidae. Museo Nacional de Ciencias Naturales (CSIC). Occurrence dataset https://doi.org/10.15468/03wqft accessed via GBIF.org on 2023-06-03.

Lobo J M (2020). BANDASCA, BAse de DAtos sobre SCArabaeidae. Museo Nacional de Ciencias Naturales (CSIC). Occurrence dataset https://doi.org/10.15468/ayfqiz accessed via GBIF.org on 2023-06-03.

Lopes L F, Venceslau L, Souto P M, Parrinha D, Queirós Neves I, Mameri D (2019). Insect Collection from the Museu Nacional de História Natural e da Ciência, Universidade de Lisboa, Portugal. Version 1.2. Museu Nacional de História Natural e da Ciência. Occurrence dataset https://doi.org/10.15468/en7jvk accessed via GBIF.org on 2023-06-03.

López Llorens J (2022). Colección Orthoptera y Dermaptera Antonio del Cerro (Museo de Zoología Ibérica Antonio Notario) UPM. School of Forestry Engineering. Technical University of Madrid. Occurrence dataset https://doi.org/10.15468/waza9m accessed via GBIF.org on 2023-06-03.

Mally R, Ward S F, Trombik J, Buszko J, Medzihorský V, Liebhold A M (2021). European occurrence records of the Robinia herbivores Parectopa robiniella, Macrosaccus robiniella and Obolodiplosis robiniella compiled from literature records and personal observations. NeoBiota. Occurrence dataset https://doi.org/10.15468/qnrsen accessed via GBIF.org on 2023-06-03.

Marabuto E (2014). Sphodromantis viridis (Forskal, 1775): New for Portugal and new records of the rare and small mantids Apteromantis aptera (Fuente, 1894) and Perlamantis allibertii Guérin-Méneville, 1843 in the country (Mantodea: Mantidae and Amorphoscelidae). Biodiversity Data Journal. Checklist dataset https://doi.org/10.15468/bvowpy accessed via GBIF.org on 2023-06-03.

Marabuto E (2014). The Afrotropical Miomantis caffra Saussure 1871 and M. paykullii Stal 1871: first records of alien mantid species in Portugal and Europe, with an updated checklist of Mantodea in Portugal (Insecta: Mantodea). Biodiversity Data Journal. Checklist dataset https://doi.org/10.15468/pkow4b accessed via GBIF.org on 2023-06-03.

Marcelino J, Borges P A V, Borges I, Soares A (2023). Eden Arthropod Azores Database. Version 1.12. Universidade dos Açores. Sampling event dataset https://doi.org/10.15468/38ccb3 accessed via GBIF.org on 2023-06-03.

Marcos Alonso J M, de Olano I, Infante Sánchez M (2023). VIT-Coleopterotheca (The Natural History Museum of Álava). Version 2.3. Natural History Museum of Alava (Museo de Ciencias Naturales de Álava / Arabako Natura Zientzien Museoa). Occurrence dataset https://doi.org/10.15470/qzikh6 accessed via GBIF.org on 2023-06-03.

Martín L, Ferreira S, Martínez J, González M A, Corley M, Grosso-Silva J M, Quaglietta L, Sousa P, Beja P, Ferreira S (2023). The InBIO Barcoding Initiative Database: DNA barcodes of Iberian Trichoptera 01. CIBIO (Research Center in Biodiversity and Genetic Resources) Portugal. Occurrence dataset https://doi.org/10.15468/hsa3kw accessed via GBIF.org on 2023-06-03.

Martín L, Martínez J, Aguín-Pombo D, González M A, plazi (2017). new endemic Synagapetus species (Trichoptera: Glossosomatidae) from Madeira Island (Portugal). Plazi.org taxonomic treatments database. Checklist dataset https://doi.org/10.11646/zootaxa.4286.2.13 accessed via GBIF.org on 2023-06-03.

Mas-Peinado P, García-París M, Ruiz J L, Buckley D, plazi (2022). The Strait of Gibraltar is an ineffective palaeogeographic barrier for some flightless darkling beetles (Coleoptera: Tenebrionidae: Pimelia). Plazi.org taxonomic treatments database. Checklist dataset https://doi.org/10.15468/8bj9u4 accessed via GBIF.org on 2023-06-03.

McElrath T (2022). Illinois Natural History Survey Insect Collection. Illinois Natural History Survey. Occurrence dataset https://doi.org/10.15468/eol0pe accessed via GBIF.org on 2023-06-03.

Medzihorský V, Trombik J, Mally R, Turčáni M, Liebhold A M (2023). Global occurrence records of Robinia specialist insect herbivores. Czech University of Life Sciences Prague, Department of Forest Protection and Entomology. Occurrence dataset https://doi.org/10.15468/zt8g2r accessed via GBIF.org on 2023-06-03.

Mertz W, Kung G, Xie W (2023). LACM Entomology Collection. Version 5.14. Natural History Museum of Los Angeles County. Occurrence dataset https://doi.org/10.15468/kc9hyp accessed via GBIF.org on 2023-06-03.

Michelsen V, plazi (2019). Macaronesian Muscidae (Diptera). I. The genus Hebecnema Schnabl with description of a new Canarian endemic species and a review of the European fauna. Plazi.org taxonomic treatments database. Checklist dataset https://doi.org/10.11646/zootaxa.4706.2.7 accessed via GBIF.org on 2023-06-03.

Michelsen V, plazi (2021). Macaronesian Muscidae (Diptera). II. The genus Limnophora Robineau-Desvoidy with description of a new Canarian endemic species. Plazi.org taxonomic treatments database. Checklist dataset https://doi.org/10.11646/zootaxa.4952.1.6 accessed via GBIF.org on 2023-06-03.

Michelsen V, plazi (2021). Macaronesian Muscidae (Diptera). III. First record of the genus Spilogona Schnabl based on two new endemic species from the Azores and Madeira. Plazi.org taxonomic treatments database. Checklist dataset https://doi.org/10.11646/zootaxa.4952.1.7 accessed via GBIF.org on 2023-06-03.

Michelsen V, plazi (2023). Macaronesian Muscidae (Diptera). IV. The genus Helina Robineau-Desvoidy, and description of the male of Spilogona maderensis Michelsen. Plazi.org taxonomic treatments database. Checklist dataset https://doi.org/10.15468/5etkkj accessed via GBIF.org on 2023-06-03.

Milotic T, Baltzinger C, Eichberg C, Eycott A, Heurich M, Müller J, Noriega J, Menendez R, Stadler J, Ádám R, Bargmann T, Bilger I, Buse J, Calatayud J, Ciubuc C, Boros G, Jay-Robert P, Kruus M, Merivee E, Miessen G, Must A, Ardali E, Preda E, Rahimi I, Rohwedder D, Slade E, Somay L, Tahmasebi P, Ziani S, Brosens D, Desmet P, Hoffmann M (2017). Dung Beetles of the Western Palaearctic. Version 1.3. Research Institute for Nature and Forest (INBO). Sampling event dataset https://doi.org/10.15468/zbazdy accessed via GBIF.org on 2023-06-03.

Miranda Chueca M Á, Barceló Seguí C (2022). AIMSurv Aedes Invasive Mosquito species harmonized surveillance in Europe. AIM-COST Action. Version 2.3. Universitat de les Illes Balears. Sampling event dataset https://doi.org/10.15470/vs3677 accessed via GBIF.org on 2023-06-03.

MNHN, Chagnoux S (2023). The Coleoptera collection (EC) of the Muséum national d'Histoire naturelle (MNHN - Paris). Version 74.319. MNHN - Museum national d'Histoire naturelle. Occurrence dataset https://doi.org/10.15468/j3leej accessed via GBIF.org on 2023-06-03.

MNHN, Chagnoux S (2023). The Lepidoptera collection (EL) of the Muséum national d'Histoire naturelle (MNHN - Paris). Version 76.319. MNHN - Museum national d'Histoire naturelle. Occurrence dataset https://doi.org/10.15468/ly36kz accessed via GBIF.org on 2023-06-03.

Moghaddam M G, Rakhshani E, Achterberg C V, Mokhtari A, plazi (2018). A study of the Iranian species of Choeras Mason (Hymenoptera: Braconidae: Microgastrinae), with the description of a new species. Plazi.org taxonomic treatments database. Checklist dataset https://doi.org/10.11646/zootaxa.4446.4.3 accessed via GBIF.org on 2023-06-03.

Moghaddam M G, Rakhshani E, Achterberg C V, Mokhtari A, plazi (2019). A taxonomic review of the genus Diolcogaster Ashmead (Hymenoptera, Braconidae, Microgastrinae) in Iran, distribution and morphological variability. Plazi.org taxonomic treatments database. Checklist dataset https://doi.org/10.11646/zootaxa.4590.1.4 accessed via GBIF.org on 2023-06-03.

Molero-Baltanás R, Gaju-Ricart M, Fišer Ž, Bach de Roca C, Mendes L F, felipe (2022). Three new species of European Coletinia Wygodzinsky (Zygentoma, Nicoletiidae), with additional records and an updated identification key. Plazi.org taxonomic treatments database. Checklist dataset https://doi.org/10.15468/8mudg4 accessed via GBIF.org on 2023-06-03.

Mosquito Alert, Escobar A, Južnič-Zonta Ž (2023). Mosquito Alert Dataset. Version 1.13. CREAF - Centre de Recerca Ecològica i Aplicacions Forestals. Occurrence dataset https://doi.org/10.15470/t5a1os accessed via GBIF.org on 2023-06-03.

Müller A, plazi (2020). Palaearctic Osmia bees of the subgenera Hemiosmia, Tergosmia and Erythrosmia (Megachilidae, Osmiini): biology, taxonomy and key to species. Plazi.org taxonomic treatments database. Checklist dataset https://doi.org/10.11646/zootaxa.4778.2.1 accessed via GBIF.org on 2023-06-03.

Muller B, Ranwashe F (2017). NMSA: Arthropod Collections (1900-2012). Version 1.1. South African National Biodiversity Institute. Occurrence dataset https://doi.org/10.15468/a4tpcb accessed via GBIF.org on 2023-06-03.

Müller N, Creuwels J (2023). Natuurmuseum Brabant, Tilburg - Invertebrates. Version 1.6. Natuurmuseum Brabant. Occurrence dataset https://doi.org/10.15468/kmsck7 accessed via GBIF.org on 2023-06-03.

Muséum d'histoire naturelle de la Ville de Genève - MHNG. Swiss Psyllid (Hemiptera) Collections - Geneva. Occurrence dataset https://doi.org/10.15468/ugcnvs accessed via GBIF.org on 2023-06-03.

Museum für Naturkunde Berlin. Animal Sound Archive. Occurrence dataset https://doi.org/10.15468/0bpalr accessed via GBIF.org on 2023-06-03.

Museum für Naturkunde Berlin. Anymals+plants - Citizen Science Data. Occurrence dataset https://doi.org/10.15468/ee6ps6 accessed via GBIF.org on 2023-06-03.

Museum of Zoology, University of Navarra M, Escribano N, Control del Estado de las Masas de Agua Superficiales, Oscoz J (2021). Freshwater samples in MZNA-INV-FRW: Macroinvertebrate samples from the water quality monitoring network along the Ebro Basin. Version 2.9. University of Navarra – Department of Environmental Biology. Sampling event dataset https://doi.org/10.15470/gkiznu accessed via GBIF.org on 2023-06-03.

Museums Victoria (2023). Museums Victoria provider for OZCAM. Occurrence dataset https://doi.org/10.15468/lp1ctu accessed via GBIF.org on 2023-06-03.

Najer T, Papousek I, Adam C, Trnka A, Quach V T, Nguyen C N, Figura R, Literak I, Sychra O, valdenar (2020). New records of Philopterus (Ischnocera: Philopteridae) from Acrocephalidae and Locustellidae, with description of one new species from Regulidae. Plazi.org taxonomic treatments database. Checklist dataset https://doi.org/10.5852/ejt.2020.632 accessed via GBIF.org on 2023-06-03.

Namyatova A A, felipe (2010). Revision of the genus Pachytomella (Heteroptera: Miridae: Orthotylinae: Halticini). Plazi.org taxonomic treatments database. Checklist dataset https://doi.org/10.5281/zenodo.4503680 accessed via GBIF.org on 2023-06-03.

National Museum of Natural History, Luxembourg (2022). Collections and observation data National Museum of Natural History Luxembourg. Occurrence dataset https://doi.org/10.15468/s2iu7d accessed via GBIF.org on 2023-06-03.

National Museum of Natural History, Luxembourg (2022). MnhnL various project records, Recorder-Lux database. Version 1.6. Occurrence dataset https://doi.org/10.15468/vmbgz9 accessed via GBIF.org on 2023-06-03.

Natural History Museum (2023). Natural History Museum (London) Collection Specimens. Occurrence dataset https://doi.org/10.5519/0002965 accessed via GBIF.org on 2023-06-03.

Natural History Museum of Utah (UMNH) (2023). Entomology Collection at the Natural History Museum of Utah. Occurrence dataset https://doi.org/10.15468/iyehoe accessed via GBIF.org on 2023-06-03.

Natural Science Research Laboratory, Museum of Texas Tech University (TTU) (2023). Texas Tech University - Invertebrate Zoology. Occurrence dataset https://doi.org/10.15468/ga4bmd accessed via GBIF.org on 2023-06-03.

naturgucker.de. naturgucker. Occurrence dataset https://doi.org/10.15468/uc1apo accessed via GBIF.org on 2023-06-03.

Naturhistorisches Museum Basel - NMB. Swiss Psyllid (Hemiptera) Collections - Basel. Occurrence dataset https://doi.org/10.15468/37eshs accessed via GBIF.org on 2023-06-03.

Naturhistorisches Museum Mainz (2017). Naturhistorisches Museum Mainz, Schmetterlingssammlung Rodenkirchen. Occurrence dataset https://doi.org/10.15468/4alimw accessed via GBIF.org on 2023-06-03.

Naturhistorisches Museum Mainz. Naturhistorisches Museum Mainz, Zoological Collection. Occurrence dataset https://doi.org/10.15468/aaknqu accessed via GBIF.org on 2023-06-03.

Naturkundemuseum im Ottoneum Kassel. Naturkundemuseum im Ottoneum Kassel, Entomological Collection. Occurrence dataset https://doi.org/10.15468/rdwi4z accessed via GBIF.org on 2023-06-03.

New York State Museum (NYSM) (2023). New York State Museum Arthropod Collection. Occurrence dataset https://doi.org/10.15468/tnjxhn accessed via GBIF.org on 2023-06-03.

Njoroge L, Achieng J, Anale J, Kamau P, Nyambati H, Wangui E (2019). Occurrence data of dragonflies available at the National Museums of Kenya's Natural History Collection, Invertebrates Zoology Section.. Version 1.3. National Museums of Kenya. Occurrence dataset https://doi.org/10.15468/dmvqri accessed via GBIF.org on 2023-06-03.

Noort B (2020). Field Study Group of the Dutch Mammal Society (NL) - 2003 - Mammal Survey Alvao Natural Park, Portugal. Version 10.4. Field Study Group of the Dutch Mammal Society. Occurrence dataset https://doi.org/10.15468/eswvar accessed via GBIF.org on 2023-06-03.

Noort B (2020). Field Study Group of the Dutch Mammal Society (NL) - 2009 - Mammal Survey Serra da Estrela, Portugal. Version 9.4. Field Study Group of the Dutch Mammal Society. Occurrence dataset https://doi.org/10.15468/ff05ok accessed via GBIF.org on 2023-06-03.

Noort B (2020). Field Study Group of the Dutch Mammal Society (NL) - 2012 - Mammal Survey Galicia, Spain. Version 2.4. Field Study Group of the Dutch Mammal Society. Occurrence dataset https://doi.org/10.15468/jcqnp1 accessed via GBIF.org on 2023-06-03.

Nunes J, Cardoso H, Valadares A, Banza P, Tomás J, Valkenburg T (2023). Portuguese Moth Recording Scheme. Version 1.3. GBIF Portugal. Occurrence dataset https://doi.org/10.15468/mbpxnd accessed via GBIF.org on 2023-06-03.

Oliveira D, Corley M, da Silva L P, Mata V A, Lopes P B, Sousa P, Beja P, Ferreira S (2021). The InBIO Barcoding Initiative Database: Lacewings (Neuroptera) 01. Version 1.2. CIBIO (Research Center in Biodiversity and Genetic Resources) Portugal. Occurrence dataset https://doi.org/10.15468/6cyrxd accessed via GBIF.org on 2023-06-03.

Oliveira D, Corley M, Lopes P B, da Silva L P, Sousa P, Beja P, Ferreira S (2021). The InBIO Barcoding Initiative Database: Lacewings (Neuroptera) 02. Version 1.1. CIBIO (Research Center in Biodiversity and Genetic Resources) Portugal. Occurrence dataset https://doi.org/10.15468/9688ju accessed via GBIF.org on 2023-06-03.

Oliveira D, Ferreira S, P. da Silva L, Lopes P B, Sousa P, Beja P (2020). The InBIO Barcoding Initiative Database: Portuguese Scorpionflies (Mecoptera, Panorpidae). Version 1.8. CIBIO (Research Center in Biodiversity and Genetic Resources) Portugal. Occurrence dataset https://doi.org/10.15468/rmj9sz accessed via GBIF.org on 2023-06-03.

Oliveira D, Ferreira S, Sousa P, Beja P (2020). The InBIO Barcoding Initiative Database: Portuguese Snakeflies (Raphidioptera). Version 1.5. CIBIO (Research Center in Biodiversity and Genetic Resources) Portugal. Occurrence dataset https://doi.org/10.15468/p7xzf3 accessed via GBIF.org on 2023-06-03.

Oliveira D, Ferreira S, Sousa P, Beja P (2020). The InBIO Barcoding Initiative Database: Portuguese Stick Insects (Phasmatodea). Version 1.4. CIBIO (Research Center in Biodiversity and Genetic Resources) Portugal. Occurrence dataset https://doi.org/10.15468/wppjdk accessed via GBIF.org on 2023-06-03.

Oromi Masoliver P (2020). Colección del Departamento de Biología Animal (Zoología) de la Universidad de La Laguna. Department of Animal Biology, Faculty of Biology, University of La Laguna. Occurrence dataset https://doi.org/10.15468/yevjxm accessed via GBIF.org on 2023-06-03.

Orrell T, Informatics and Data Science Center, Digital Stewardship Team (2023). NMNH Material Samples (USNM). Version 1.53. National Museum of Natural History, Smithsonian Institution. Occurrence dataset https://doi.org/10.15468/jb9tdf accessed via GBIF.org on 2023-06-03.

Orrell T, Informatics Office (2023). NMNH Extant Specimen Records (USNM, US). Version 1.69. National Museum of Natural History, Smithsonian Institution. Occurrence dataset https://doi.org/10.15468/hnhrg3 accessed via GBIF.org on 2023-06-03.

Ortiz A S (2021). Checklist of the Crambidae of the Region of Murcia (Spain) with new records (Lepidoptera: Pyraloidea, Crambidae). Version 1.3. Animal Biology Section, Zoology and Phisic Antropology Department, Murcia University. Checklist dataset https://doi.org/10.15470/kffxc0 accessed via GBIF.org on 2023-06-03.

Ortiz A S (2021). Checklist of the Pyralidae of the Region of Murcia (Spain) with new records (Lepidoptera: Pyraloidea, Pyralidae). Version 1.1. Animal Biology Section, Zoology and Phisic Antropology Department, Murcia University. Checklist dataset https://doi.org/10.15470/a6fcav accessed via GBIF.org on 2023-06-03.

Ortiz A S (2021). First record and DNA barcoding of Donacaula niloticus (Zeller, 1867) from the Iberian Peninsula (Lepidoptera: Crambidae). Biodiversity Data Journal. Checklist dataset https://doi.org/10.3897/bdj.9.e70193 accessed via GBIF.org on 2023-06-03.

Oteman B (2021). BCE butterfly data central and southern Europe. Version 1.9. Butterfly Conservation Europe. Occurrence dataset https://doi.org/10.15468/ifyqwb accessed via GBIF.org on 2023-06-03.

Oteman B (2021). BCE butterfly data expert holiday data throughout Europe. Version 1.8. Butterfly Conservation Europe. Occurrence dataset https://doi.org/10.15468/6nzceo accessed via GBIF.org on 2023-06-03.

Oteman B (2021). BCE butterfly data Spain. Version 1.7. Butterfly Conservation Europe. Occurrence dataset https://doi.org/10.15468/ry5gjx accessed via GBIF.org on 2023-06-03.

Page R D M, Kraemer M U G, Sinka M E, Duda K A, Mylne A, Shearer F M, Brady O J, Messina J P, Barker C M, Moore C G, Carvalho R G, Coelho G E, Van Bortel W, Hendrickx G, Schaffner F, Wint G R W, Elyazar I R F, Teng H, Hay S I. Global compendium of Aedes albopictus occurrence. Institute of Biodiversity, Animal Health and Comparative Medicine, College of Medical, Veterinary and Life Sciences, University of Glasgow. Occurrence dataset https://doi.org/10.15468/7apj8n accessed via GBIF.org on 2023-06-03.

París M, Blay A, Alonso Zarazaga M Á (2020). Museo Nacional de Ciencias Naturales, Entomología. Museo Nacional de Ciencias Naturales (CSIC). Occurrence dataset https://doi.org/10.15468/pqfubx accessed via GBIF.org on 2023-06-03.

Pauly A, Noël G, Sonet G, Notton D G, Boevé J, plazi (2019). Integrative taxonomy resuscitates two species in the Lasioglossum villosulum complex (Kirby, 1802) (Hymenoptera: Apoidea: Halictidae). Plazi.org taxonomic treatments database. Checklist dataset https://doi.org/10.5852/ejt.2019.541 accessed via GBIF.org on 2023-06-03.

Pauwels O, Samyn Y, Vandenberghe T (2021). RBINS DaRWIN. Royal Belgian Institute of Natural Sciences. Occurrence dataset https://doi.org/10.15468/qxy4mc accessed via GBIF.org on 2023-06-03.

Paz D, Román I, Ramírez L, López D, Andreu A C, Díaz-Delgado R, Bustamante J, Márquez Ferrando R (2023). Long-term monitoring of roller dung beetles (Scarabaeinae) (abundance and distribution) in Doñana 2004-2012. Version 2.3. Estación Biológica de Doñana (CSIC). Sampling event dataset https://doi.org/10.15470/0rfdtn accessed via GBIF.org on 2023-06-03.

Peris-Felipo F J (2014). Aspilota-group (Hymenoptera: Braconidae: Alysiinae) diversity in Mediterranean Natural Parks of Spain. Biodiversity Data Journal. Checklist dataset https://doi.org/10.15468/yasgbi accessed via GBIF.org on 2023-06-03.

Pina S (2017). The Orthoptera of Castro Verde Special Protection Area (Southern Portugal). CIBIO (Research Center in Biodiversity and Genetic Resources) Portugal. Sampling event dataset https://doi.org/10.15468/byd0kt accessed via GBIF.org on 2023-06-03.

Pino Pérez J J (2022). Colección de Artrópodos de la Asociación BIGA para el estudio del patrimonio natural de Galicia: ABIGA. Version 1.10. Colecciones naturales de la Asociación BIGA para el estudio del patrimonio natural de Galicia: FBIGA y ABIGA.. Occurrence dataset https://doi.org/10.15468/nogcsx accessed via GBIF.org on 2023-06-03.

Pinya Fernández S, Lassnig Ballester N, Truyols Henares F, Mascaró Triay M, Canyelles Ferrà X, Perelló Alomar E (2022). Seguimiento y control científico de las capturas de Cerambyx cerdo en montes públicos de Mallorca. Version 1.1. Universitat de les Illes Balears. Sampling event dataset https://doi.org/10.15470/nrpzuo accessed via GBIF.org on 2023-06-03.

Pollet M, Andrade R, Gonçalves A, Álvarez Fidalgo P, Camaño Portela J L, Belin F, Mortelmans J, Brosens D, Stark A (2022). Medetera species with multi-coloured eyes in southern Europe. Research Institute for Nature and Forest (INBO). Occurrence dataset https://doi.org/10.15468/s8c7n9 accessed via GBIF.org on 2023-06-03.

Pollet M, Andrade R, Gonçalves A, Andrade P, Jacinto V, Almeida J, De Braekeleer A, Van Calster H, Brosens D (2022). Dolichopodidae of Portugal - dataset 2009-2018. Version 1.7. Research Institute for Nature and Forest (INBO). Sampling event dataset https://doi.org/10.15468/latzp3 accessed via GBIF.org on 2023-06-03.

Prirodoslovni muzej Slovenije. World flea collection of Slovenian Museum of Natural History (excluding Slovenia). Occurrence dataset https://doi.org/10.15468/th6dfi accessed via GBIF.org on 2023-06-03.

PUIG M A (2020). Centre d'Estudis Avançats de Blanes, Macroinvertebrados Limno. Centro de Estudios Avanzados de Blanes (CSIC). Occurrence dataset https://doi.org/10.15468/3gwhkc accessed via GBIF.org on 2023-06-03.

QUAINTENNE Gwenaël N, BRUGEL Eric N, JOMAT Loïc N, ANDRÉ Mathieu N, CHEVALIER Thomas N, Inventaire National du Patrimoine Naturel (2020). Synthèse 10x10km de la faune sauvage des Directives Oiseaux et Habitats-Faune-Flore recensée via les portails gérés par la LPO de 2013 à 2017. Version 1.1. UMS PatriNat (OFB-CNRS-MNHN), Paris. Occurrence dataset https://doi.org/10.15468/1daa3s accessed via GBIF.org on 2023-06-03.

Questagame (2022). Earth Guardians Weekly Feed. Occurrence dataset https://doi.org/10.15468/slqqt8 accessed via GBIF.org on 2023-06-03.

Quirce Vázquez C, Ricarte Sabater A (2020). Instituto de Investigación CIBIO, Universidad de Alicante: CEUA. Research Institute CIBIO (Centro Iberoamericano de la Biodiversidad), University of Alicante. Occurrence dataset https://doi.org/10.15468/upgeq4 accessed via GBIF.org on 2023-06-03.

Racz G (2023). Harold W. Manter Laboratory of Parasitology Collection (HWML) Parasite Collection (Arctos). Version 1.33. University of Nebraska State Museum. Occurrence dataset https://doi.org/10.15468/kfgfzr accessed via GBIF.org on 2023-06-03.

Raposeiro P, Balibrea A, Riva J, Ritter C, Gonçalves V (2021). Macroinvertebrates distribution in Madeira Island streams (Portugal). Version 1.9. Universidade dos Açores. Occurrence dataset https://doi.org/10.15468/48axjg accessed via GBIF.org on 2023-06-03.

Reboleira A S P S, Fresneda J, Salgado J M, plazi (2017). A new species of Speonemadus from Portugal, with the revision of the escalerai-group (Coleoptera, Leiodidae). Plazi.org taxonomic treatments database. Checklist dataset https://doi.org/10.5852/ejt.2017.261 accessed via GBIF.org on 2023-06-03.

Rey Muñiz X L (2021). SGHN - Biota Galicia. Version 2.3. Sociedade Galega de Historia Natural (SGHN). Occurrence dataset https://doi.org/10.15470/sxfyxw accessed via GBIF.org on 2023-06-03.

Ribera I, Foster G N, plazi (2018). Report of Frank Balfour-Browne’s collecting in Gran Canaria and Madeira (1932 - 1933), with the description of Ochthebius (Cobalius) lanthanus sp. nov. (Coleoptera, Hydraenidae). Plazi.org taxonomic treatments database. Checklist dataset https://doi.org/10.11646/zootaxa.4524.1.4 accessed via GBIF.org on 2023-06-03.

Roca-Cusachs M, Kment P, plazi (2022). Joppeicus paradoxus (Hemiptera: Heteroptera: Joppeicidae): a new alien species in the European Union?. Plazi.org taxonomic treatments database. Checklist dataset https://doi.org/10.15468/n5dqw7 accessed via GBIF.org on 2023-06-03.

Ros Candeira A, Pérez-Luque A J, Suárez Muñoz M, Bonet García F J, Hódar Correa J A (2018). Dataset of occurrence and incidence of pine processionary moth in Andalusia (South Spain). Version 2.4. Sierra Nevada Global Change Observatory. Andalusian Environmental Center, University of Granada, Regional Government of Andalusia. Sampling event dataset https://doi.org/10.15470/s1mxjb accessed via GBIF.org on 2023-06-03.

Rosana ZUCCHELLI N, Inventaire National du Patrimoine Naturel (2020). Données opportunistes rhopalocères 2010/2015 collectées par l'association EcoGIS. Version 1.1. UMS PatriNat (OFB-CNRS-MNHN), Paris. Occurrence dataset https://doi.org/10.15468/nqhnuv accessed via GBIF.org on 2023-06-03.

Runnel V. My naturesounds - nature observations with sound recordings. PlutoF. Occurrence dataset https://doi.org/10.15468/hnheni accessed via GBIF.org on 2023-06-03.

Rutherford M G, Auguste R J (2018). UWIZM International Lepidoptera Collection. Version 1.3. The University of the West Indies Zoology Museum (UWIZM). Occurrence dataset https://doi.org/10.15468/qe6juh accessed via GBIF.org on 2023-06-03.

Ruzzier E (2022). New species and new records of exotic Scolytinae (Coleoptera, Curculionidae) in Europe. Biodiversity Data Journal. Checklist dataset https://doi.org/10.3897/bdj.10.e93995 accessed via GBIF.org on 2023-06-03.

Saarenmaa H (2019). Lepidoptera collection of Hannu Saarenmaa. Department of Forest Sciences. Sampling event dataset https://doi.org/10.15468/rmd2mk accessed via GBIF.org on 2023-06-03.

Sánchez Gea J F (2020). Dpto. de Zoología y Antropología Física, Universidad de Murcia. Colección de Entomología, Coleoptera.. Animal Biology Section, Zoology and Phisic Antropology Department, Murcia University. Occurrence dataset https://doi.org/10.15468/lwzpx5 accessed via GBIF.org on 2023-06-03.

Sandoval Cortés P J, Sánchez Piñero F (2021). Colección de Coleoptera (Tenebrionidae) del sureste de la Península Ibérica de la Universidad de Granada. Version 1.6. Dept. of Zoology, Faculty of Science, University of Granada. Occurrence dataset https://doi.org/10.15468/3qftfc accessed via GBIF.org on 2023-06-03.

Sandoval Cortés P J, Sánchez Piñero F (2022). Colección de Coleoptera (Scarabaeoidea) del sureste de la Península Ibérica de la Universidad de Granada. Version 1.6. Dept. of Zoology, Faculty of Science, University of Granada. Occurrence dataset https://doi.org/10.15468/gbamtm accessed via GBIF.org on 2023-06-03.

Sanglas A, Roman J, Palomares F (2023). Prey identification of free-ranging domestic cats (Felis catus) from rural and natural areas of Spain through scat analysis. Version 1.3. Estación Biológica de Doñana (CSIC). Sampling event dataset https://doi.org/10.15470/yncxe5 accessed via GBIF.org on 2023-06-03.

Scharff N, Pape T, Solodovnikov A, Vilhelmsen L, Enghoff H, Illum A A, Selvantharan S G, Pedersen J (2023). NHMD Entomology Collection. Natural History Museum of Denmark. Occurrence dataset https://doi.org/10.15468/nnobcm accessed via GBIF.org on 2023-06-03.

Schelhaas M, Varis S (2015). Database on Forest Disturbances in Europe. Version 16.2. European Forest Institute. Occurrence dataset https://doi.org/10.15468/b7s6oy accessed via GBIF.org on 2023-06-03.

Schifani E, Alicata A, plazi (2023). Nomenclatural changes on some Mediterranean Aphaenogaster Mayr, 1853 taxa (Hymenoptera, Formicidae). Plazi.org taxonomic treatments database. Checklist dataset https://doi.org/10.15468/9bs9hq accessed via GBIF.org on 2023-06-03.

Seifert B, Schultz R, Agosti D (2009). A taxonomic revision of the Formica rufibarbis Fabricius, 1793 group (Hymenoptera: Formicidae).. Plazi.org taxonomic treatments database. Checklist dataset https://doi.org/10.15468/aep6re accessed via GBIF.org on 2023-06-03.

Selnekovič D (2021). Taxonomic revision of Mordellistena hirtipes species-complex with new distributional records (Insecta, Coleoptera, Mordellidae). Version 1.4. ZooKeys. Occurrence dataset https://doi.org/10.15468/pkhkul accessed via GBIF.org on 2023-06-03.

Senckenberg. Collection Coleoptera SMF. Occurrence dataset https://doi.org/10.15468/kr6iaj accessed via GBIF.org on 2023-06-03.

Senckenberg. Orthoptera - SMF. Occurrence dataset https://doi.org/10.15468/4m5gwk accessed via GBIF.org on 2023-06-03.

Shavrin A V, Zanetti A, plazi (2021). Review of the genus Lesteva Latreille, 1797 (Coleoptera: Staphylinidae: Omaliinae Anthophagini) of the Iberian Peninsula. Plazi.org taxonomic treatments database. Checklist dataset https://doi.org/10.11646/zootaxa.4966.4.1 accessed via GBIF.org on 2023-06-03.

Sikes D (2023). KWP Lepidoptera Collection (Arctos). University of Alaska Museum of the North. Occurrence dataset https://doi.org/10.15468/u4fp2u accessed via GBIF.org on 2023-06-03.

Sikes D (2023). UAM Insect Collection (Arctos). Version 39.75. University of Alaska Museum of the North. Occurrence dataset https://doi.org/10.15468/qs8slz accessed via GBIF.org on 2023-06-03.

Simões P (2014). First record of Tettigettalna mariae Quartau & Boulard, 1995 (Insecta: Hemiptera: Cicadoidea) in Spain. Biodiversity Data Journal. Checklist dataset https://doi.org/10.15468/ldzolb accessed via GBIF.org on 2023-06-03.

Simões P (2014). Tettigettalna josei (Boulard, 1982) (Hemiptera: Cicadoidea): first record in Spain, with notes on the distribution, genetic variation and behaviour of the species. Biodiversity Data Journal. Checklist dataset https://doi.org/10.15468/jzuuqo accessed via GBIF.org on 2023-06-03.

Slieker F J A, van der Es H, Andeweg R, Langeveld B W, Schnörr S (2023). Natural History Museum Rotterdam - Specimens. Version 1.42. Natural History Museum Rotterdam. Occurrence dataset https://doi.org/10.15468/kwqaay accessed via GBIF.org on 2023-06-03.

Soares A O, Borges I, Calado H, Borges P A V (2021). Biodiversity data of ladybeetles (Coleoptera: Coccinellidae) of the Azores archipelago (Portugal). Version 1.5. Universidade dos Açores. Sampling event dataset https://doi.org/10.15468/m74aqv accessed via GBIF.org on 2023-06-03.

Soler J M, Falcó-Garí J V, Herrero B, Aquino D A, plazi (2021). The Anagyrus (Hymenoptera: Encyrtidae) parasitoids of the obscure mealybug Pseudococcus viburni (Hemiptera: Pseudococcidae) in Spain, with description of a new species. Plazi.org taxonomic treatments database. Checklist dataset https://doi.org/10.11646/zootaxa.4980.1.5 accessed via GBIF.org on 2023-06-03.

Song H, Johnson N (2018). Stuart M. Fullerton Collection of Arthropods (UCFC), University of Central Florida. Version 85.36. Museum of Biological Diversity, The Ohio State University. Occurrence dataset https://doi.org/10.15468/kyulwg accessed via GBIF.org on 2023-06-03.

Soon V. University of Tartu Natural History Museum and Botanical Garden Zooloogical Collections. University of Tartu, Natural History Museum and Botanical Garden. Occurrence dataset https://doi.org/10.15468/6hfnux accessed via GBIF.org on 2023-06-03.

Sousa P, Grosso-Silva J M, Andrade R, Beja P, Ferreira S (2021). The InBIO Barcoding Initiative Database: Hemiptera 01. Version 1.2. CIBIO (Research Center in Biodiversity and Genetic Resources) Portugal. Occurrence dataset https://doi.org/10.15468/8hn25y accessed via GBIF.org on 2023-06-03.

Starý J, Oboňa J, plazi (2018). Palaearctic species of Thaumastoptera (s. str.) Mik (Diptera: Limoniidae). Plazi.org taxonomic treatments database. Checklist dataset https://doi.org/10.11646/zootaxa.4394.2.5 accessed via GBIF.org on 2023-06-03.

Steenis J V, plazi (2020). A new species of the genus Myolepta Newman (Diptera: Syrphidae), with short description and key to all species of the M. vara subgroup. Plazi.org taxonomic treatments database. Checklist dataset https://doi.org/10.11646/zootaxa.4750.3.4 accessed via GBIF.org on 2023-06-03.

Stuke J, Barták M, plazi (2019). Records of Carnidae from the collection of Miroslav Barták (Diptera: Carnidae), with the description of five new species. Plazi.org taxonomic treatments database. Checklist dataset https://doi.org/10.11646/zootaxa.4567.2.6 accessed via GBIF.org on 2023-06-03.

Tasmanian Museum and Art Gallery (2023). Tasmanian Museum and Art Gallery provider for OZCAM. Occurrence dataset https://doi.org/10.15468/ijp8p9 accessed via GBIF.org on 2023-06-03.

Teixeira M B, Soares A O, Calvet M, Peñalver Á, Monteiro H, Frias J, Borges P A V, Simões N (2023). Monitoring ground arthropods in maize and pasture fields of São Miguel and São Jorge Islands: IPM-Popillia Project. Version 1.1. Universidade dos Açores. Sampling event dataset https://doi.org/10.15468/4cnhw9 accessed via GBIF.org on 2023-06-03.

Texas A&M University Insect Collection (2023). Texas A&M University Insect Collection. Occurrence dataset https://doi.org/10.15468/caprqh accessed via GBIF.org on 2023-06-03.

The International Barcode of Life Consortium (2023). International Barcode of Life project (iBOL). Occurrence dataset https://doi.org/10.15468/inygc6 accessed via GBIF.org on 2023-06-03.

Thierry Laporte N, Inventaire National du Patrimoine Naturel (2020). Données de rhopalocères collectées en 2011 et 2012 par le CEN Aquitaine / Diagnostic écologique Natura 2000 de Haute Soule et du Barétous. Version 1.1. UMS PatriNat (OFB-CNRS-MNHN), Paris. Occurrence dataset https://doi.org/10.15468/cqet0g accessed via GBIF.org on 2023-06-03.

Thomaes A, Barbalat S, Bardiani M, Bower L, Campanaro A, Soutinho J G, Harvey D, Hawes C, Kadej M, Méndez M, Rink M, Rossi De Gasperis S, Ruyts S, Šerić Jelaska L, Smit J, Smolis A, Vrezec A, Brosens D (2023). European stag beetle monitoring network: transect observations. Version 1.9. Research Institute for Nature and Forest (INBO). Occurrence dataset https://doi.org/10.15468/j69hbu accessed via GBIF.org on 2023-06-03.

Tiroler Landesmuseum Ferdinandeum. Tiroler Landesmuseum Ferdinandeum. Occurrence dataset https://doi.org/10.15468/eepzxm accessed via GBIF.org on 2023-06-03.

Torralba-Burrial A (2018). Iberian Odonata distribution: data of the BOS Arthropod Collection (Univ. Oviedo, Spain). Department of Organisms and Systems Biology. University of Oviedo. Occurrence dataset https://doi.org/10.15468/5chm8b accessed via GBIF.org on 2023-06-03.

Traveset A, Díaz Lorca A, Lazaro Castillo A, Diaz Lorca A (2023). Institut Mediterrani d'Estudis Avançats (CSIC-UIB): IMEDEA-INSECTA. Instituto Mediterráneo de Estudios Avanzados (CSIC). Occurrence dataset https://doi.org/10.15468/cmbvkt accessed via GBIF.org on 2023-06-03.

UMMZ Insects Division Data Group, LSA IT A (2023). University of Michigan Museum of Zoology, Division of Insects. Version 1.58. University of Michigan Museum of Zoology. Occurrence dataset https://doi.org/10.15468/tmxd7n accessed via GBIF.org on 2023-06-03.

University of Alberta Museums, La France D, Becker-Burns A (2023). University of Alberta E. H. Strickland Entomological Museum (UASM). University of Alberta Museums. Occurrence dataset https://doi.org/10.18165/9enbmt accessed via GBIF.org on 2023-06-03.

University of Bergen (2023). Entomological collections, UiB. Version 1.2255. Occurrence dataset https://doi.org/10.15468/irppio accessed via GBIF.org on 2023-06-03.

University of Kentucky (2023). Hymenoptera Institute Collection. Occurrence dataset https://doi.org/10.15468/hg5ph7 accessed via GBIF.org on 2023-06-03.

University of Oslo (2023). Entomology, Oslo (O) UiO. Version 1.2162. Occurrence dataset https://doi.org/10.15468/c3oka6 accessed via GBIF.org on 2023-06-03.

University of Oulu. Invertebrate collection of University of Oulu Zoological Museum. Version 1.0. Occurrence dataset https://doi.org/10.15468/d0nqsj accessed via GBIF.org on 2023-06-03.

University of Tennessee - Chattanooga (UTC) (2023). University of Tennessee at Chattanooga Insect Collection. Occurrence dataset https://doi.org/10.15468/yllp1i accessed via GBIF.org on 2023-06-03.

University of Wisconsin - Madison (2021). Wisconsin Insect Research Collection. Occurrence dataset https://doi.org/10.15468/237bna accessed via GBIF.org on 2023-06-03.

van Nieukerken E J, Sobczyk T, Creuwels J (2021). Stigmella stettinensis (Nepticulidae) specimen data. Version 1.1. Naturalis Biodiversity Center. Occurrence dataset https://doi.org/10.15468/hxqsdw accessed via GBIF.org on 2023-06-03.

van Noort D S, Ranwashe F (2020). IZIKO South Africa Museum Collection (1800-2013). Version 1.4. South African National Biodiversity Institute. Occurrence dataset https://doi.org/10.15468/vf3ko1 accessed via GBIF.org on 2023-06-03.

Vasar M, Davison J, Sepp S, Mucina L, Oja J, Al-Quraishy S, Anslan S, Bahram M, Bueno C G, Cantero J J, Decocq G, Fraser L, Hiiesalu I, Hozzein W N, Koorem K, Meng Y, Moora M, Onipchenko V, Öpik M, Pärtel M, Vahter T, Tedersoo L, Zobel M (2022). Global soil microbiomes: A new frontline of biome-ecology research. PlutoF. Occurrence dataset https://doi.org/10.1111/geb.13487 accessed via GBIF.org on 2023-06-03.

Vellinga W P (2023). Xeno-canto - Wildlife sounds from around the world. Xeno-canto Foundation for Nature Sounds. Occurrence dataset https://doi.org/10.15468/qv0ksn accessed via GBIF.org on 2023-06-03.

Venceslau L, Lopes L F, Loureiro M, Mameri D, Mendes L (2019). IICT Tabanidae Collection. Instituto de Investigação Científica Tropical. Occurrence dataset https://doi.org/10.15468/rwohkc accessed via GBIF.org on 2023-06-03.

Vieira V, Oliveira L, Soares A O, Borges P A V, Borges I, Tavares J (2022). Diversity of Lepidoptera recorded in a forest nursery of Nordeste county on São Miguel Island (Azores). Version 1.8. Universidade dos Açores. Sampling event dataset https://doi.org/10.15468/965r3p accessed via GBIF.org on 2023-06-03.

Villares Muyo J M, Ruiz Franco B (2020). Ministerio de Medio Ambiente, y Medio Rural y Marino. Dirección General de Medio Natural y Política Forestal. Inventario Nacional de Biodiversidad 2007, Invertebrados. Spanish Ministry for Ecological Transition and Demographic Challenge. Occurrence dataset https://doi.org/10.15468/397ezp accessed via GBIF.org on 2023-06-03.

Virginia Tech Insect Collection (2023). Virginia Polytechnic Institute and State University Insect Collection. Occurrence dataset https://doi.org/10.15468/m2htqa accessed via GBIF.org on 2023-06-03.

Vorst O, Creuwels J (2023). Naturalis Biodiversity Center (NL) - Coleoptera. Naturalis Biodiversity Center. Occurrence dataset https://doi.org/10.15468/jrjojf accessed via GBIF.org on 2023-06-03.

Wagner R, Andrade R, Gonçalves A, plazi (2022). Moth flies (Diptera, Psychodidae) from Portugal with descriptions of a new genus new species and additions to the fauna of the Iberian Peninsula. Plazi.org taxonomic treatments database. Checklist dataset https://doi.org/10.15468/8xpxqu accessed via GBIF.org on 2023-06-03.

Wagner R, Withers P, plazi (2020). The West-Palearctic species of the genus Tonnoiriella Vaillant, 1971 (Diptera: Psychodidae, Psychodinae). Plazi.org taxonomic treatments database. Checklist dataset https://doi.org/10.11646/zootaxa.4728.2.2 accessed via GBIF.org on 2023-06-03.

Wallon S, Elias R B, Borges P A V (2023). Monitoring grassland's arthropods in a in situ climate change experimentation (Terceira, Azores, Portugal). Version 1.4. Universidade dos Açores. Sampling event dataset https://doi.org/10.15468/u2xh5g accessed via GBIF.org on 2023-06-03.

Wanat M (2017). Collection of Coleoptera. Wrocław University, Museum of Natural History. Occurrence dataset https://doi.org/10.15468/mwv2b0 accessed via GBIF.org on 2023-06-03.

Wanat M (2017). Collection of Hymenoptera. Wrocław University, Museum of Natural History. Occurrence dataset https://doi.org/10.15468/yhgaj8 accessed via GBIF.org on 2023-06-03.

Wild A (2023). University of Texas, Biodiversity Center, Entomology Collection (UTIC). Version 1.157. University of Texas at Austin, Biodiversity Collections. Occurrence dataset https://doi.org/10.15468/sanyq7 accessed via GBIF.org on 2023-06-03.

Wilhelmsen Å I, Bachmann L, Rindal E (2023). Crustacea collection (Cru) at UiO NHM, Oslo. Version 1.6. University of Oslo. Occurrence dataset https://doi.org/10.15468/efw98v accessed via GBIF.org on 2023-06-03.

Williams K, Ranwashe F (2016). DNSM: Durban Natural Science Museum Insect Collection (1900-2011). Version 1.2. South African National Biodiversity Institute. Occurrence dataset https://doi.org/10.15468/bsnpad accessed via GBIF.org on 2023-06-03.

Williams P H, Altanchimeg D, Byvaltsev A, Jonghe R D, Jaffar S, Japoshvili G, Kahono S, Liang H, Mei M, Monfared A, Nidup T, Raina R, Ren Z, Thanoosing C, Zhao Y, Orr M C, valdenar (2020). Widespread polytypic species or complexes of local species? Revising bumblebees of the subgenus Melanobombus world-wide (Hymenoptera, Apidae, Bombus). Plazi.org taxonomic treatments database. Checklist dataset https://doi.org/10.5852/ejt.2020.719.1107 accessed via GBIF.org on 2023-06-03.

Wood T J, Cross I, Baldock D W, plazi (2020). Updates to the bee fauna of Portugal with the description of three new Iberian Andrena species (Hymenoptera: Apoidea: Anthophila). Plazi.org taxonomic treatments database. Checklist dataset https://doi.org/10.11646/zootaxa.4790.2.1 accessed via GBIF.org on 2023-06-03.

Wood T J, Ghisbain G, Michez D, Praz C J, felipe (2021). Revisions to the faunas of Andrena of the Iberian Peninsula and Morocco with the descriptions of four new species (Hymenoptera: Andrenidae). Plazi.org taxonomic treatments database. Checklist dataset https://doi.org/10.5852/ejt.2021.758.1431 accessed via GBIF.org on 2023-06-03.

WoRMS Editorial Board (2021). Type locality distributions from the World Register of Marine Species. Available from http://www.marinespecies.org at VLIZ. Accessed on YYYY-MM-DD. https://doi.org/10.15468/nmcdsv accessed via GBIF.org on 2023-06-03.

Wunderle V A P, carolina (2008). On the Alevonota species of the Western Palaearctic region (Coleoptera: Staphylinidae: Aleocharinae: Athetini). Plazi.org taxonomic treatments database. Checklist dataset https://doi.org/10.21248/contrib.entomol.58.1.145-189 accessed via GBIF.org on 2023-06-03.

Zamisa S, Midgley J (2022). KwaZulu-Natal Museum-Entomology-collection. Version 1.1. KwaZulu-Natal Museum. Occurrence dataset https://doi.org/10.15468/vf6as7 accessed via GBIF.org on 2023-06-03.

Zhuang V (2023). UTEP Insects (Arctos). Version 1.68. University of Texas at El Paso Biodiversity Collections. Occurrence dataset https://doi.org/10.15468/cfddho accessed via GBIF.org on 2023-06-03.

Zlatkov B, Huemer P, carolina (2017). Allopatric cryptic diversity in the alpine species complex Phtheochroa frigidana s. lat. (Lepidoptera: Tortricidae). Plazi.org taxonomic treatments database. Checklist dataset https://doi.org/10.5852/ejt.2017.368 accessed via GBIF.org on 2023-06-03.

Zoologische Staatssammlung München/Staatliche Naturwissenschaftliche Sammlungen Bayerns. Zoologische Staatssammlung Muenchen - International Barcode of Life (iBOL) - Barcode of Life Project Specimen Data. Occurrence dataset https://doi.org/10.15468/tfpnkp accessed via GBIF.org on 2023-06-03.
